# Green RP-UPLC Method for Simultaneous Determination of Cyclopentolate and Organic Impurities Using DoE and Sustainability Metrics

**DOI:** 10.1155/ianc/8827373

**Published:** 2025-09-16

**Authors:** Bandar R. Alsehli, Abdullah H. Alluhayb, Lateefa A. Al-Khateeb, Sayed M. Saleh, Ammena Y. Binsaleh, Mahmoud A. Mohamed

**Affiliations:** ^1^Department of Chemistry, Faculty of Science, Taibah University, Al-Madinah Al-Munawarah 30002, Saudi Arabia; ^2^Department of Chemistry, College of Science, Qassim University, Buraidah 51452, Saudi Arabia; ^3^Chemistry Department, Faculty of Science, King Abdulaziz University, P.O. Box 80200, Jeddah 21589, Saudi Arabia; ^4^Department of Pharmacy Practice, College of Pharmacy, Princess Nourah bint Abdulrahman University, P.O. Box 84428, Riyadh 11671, Saudi Arabia; ^5^Hikma Pharmaceutical Company, Beni-Suef 62511, Egypt

**Keywords:** Box–Behnken, cyclopentolate, organic impurities, RP-UPLC, sustainability assessment tools

## Abstract

A significant improvement in sustainability and efficiency is achievable through green and white chemistry. As part of this study, sustainability assessment tools were used to assess the environmental impact and practicality of an innovative, straightforward RP-UPLC method to analyze cyclopentolate (CLO) and its organic impurities simultaneously in pure and ophthalmic solutions at the same time. An optimization strategy based on Box–Behnken design was employed to minimize experimental runs while optimizing chromatographic conditions. Using this design, four critical variables were evaluated comprehensively—ethanol percentage in the mobile phase, pH, column temperature, and flow rate—on chromatographic responses such as retention time, resolution between CLO and impurity, and theoretical plate count. As a result of desirable and overlay plots, an optimal condition was selected: 65:25, v/v, ethanol and buffer, pH 4.25, 0.3 mL/min flow rate, and 4°C and 25°C sample and column oven temperatures, respectively, and the main peak retained for a little more than 3 min. The calibration curves for CLO and impurities at concentrations from 5 to 50 μg/mL and 1 to 20 μg/mL showed a correlation value of 0.9998. Recoveries are ±15% of the actual amounts, which is acceptable. RP-UPLC has been extensively designed for the coincidental estimation of anticholinergic drugs and their impurities. A combination of white and green tools was used to assess the method's environmental impact. ICH guidelines have been followed to validate the suggested strategy. This approach offers a reliable, fast, and eco-friendly solution for routine pharmaceutical quality control of anticholinergic agents.

## 1. Introduction

It is crucial to identify and control impurities in pharmaceutical products to ensure their safety and effectiveness. Impurities, including pharmaceutical formulation, can adversely affect a medication's potency, safety, and efficacy [1, 2]. Pharmaceutical impurities are strictly regulated by FDA and EMA regulations. Therefore, ensuring organic impurities can be accurately assessed is imperative for the quality control of pharmaceuticals, ensuring that impurities are within acceptable thresholds and safety [3, 4].

Chromatography practices associated with environmental awareness are sensational. A rising trend in the use of ethanol over other solvents is due to its safety and sustainability. There is increasing evidence that substitute reagents can produce similar results to conventional solvents [5, 6]. Science research can benefit significantly from sustainable alternatives. Soon, this field will see several exciting developments. Many factors can affect pharmaceuticals, including degradation and impurities. The safety, effectiveness, and quality of medicines must consider environmental pollutants. Overdosing on medications can lead to their ineffectiveness. It is imperative that pharmaceuticals are free of harmful contaminants so that patients can remain safe [7, 8].

Despite its versatility and reliability, HPLC is widely applied in various fields. Due to its unique precision, responsiveness, and superior accuracy, this technology has acquired a noteworthy reputation for detecting diverse elements across various substrates. Due to its minimal solvent quantity requirements, speedy analysis time, and efficacy, in pharmaceutical operations, HPLC is widely used for evaluating APIs, related substances, and for quality control of finished products [9].

Among the biggest challenges facing the pharmaceutical industry today is developing methods which are not only effective and environmentally friendly, but are also operationally feasible. By adopting green analytical chemistry (GAC), which minimizes environmental impact by using safer solvents, reducing waste generation, and adopting energy-efficient procedures, this shift has led to a shift toward GAC principles. Providing a complement to this approach is the concept of white analytical chemistry (WAC), which emphasizes the simplicity, cost-effectiveness, and robustness of analytical methods, ensuring that GAC and WAC are both sustainable and suitable for routine use [10]. Sustainable initiatives have incorporated GAC and WAC. A wide range of environmental impact metrics have been developed over the last 10 years to assess the impact of new analytical procedures. As the chemical industry faces an uncertain future, green and white chemistry are becoming increasingly important. GAC and WAC play a crucial role in the development of sustainable practices and the implementation of sustainable chemistry programs. During the past two decades, GAC and WAC have contributed significantly to the advancement of sustainable chemistry [11, 12]. The chemical industry is poised to transform because of manufacturing initiatives that promote sustainability. Green chemistry principles have played a significant role in promoting sustainable design. Several criteria of green chemistry must be adhered to by chemists to realize these advancements [13]. During the next several decades, green chemistry will play an even more critical role in preventing waste and fostering sustainable development. Method validation parameters can provide insight into how green an analysis technique is [14]. As the ecological of its methods is verified, analytical chemistry will be more accepted by the community. Bringing green chemistry initiatives into the future is a key to a sustainable future. As a result of recent efforts to develop greener quantitative analysis methods, a growing movement has emerged. Safety, health, and environmental improvements need to be made to analytical processes. There is a growing trend among pharmaceutical companies to adopt eco-friendly analytical methods, and environmental responsibility is becoming increasingly crucial to the analytical community. Solvents and waste production should be considered carefully when developing new strategies. Though analytical chemistry has progressed towards sustainability, there is still a lot more to be done [15].

**Figure 1:**
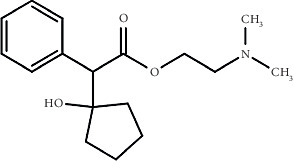
Chemical structure of CLO.

There is a possibility that CLO (Figure 1) eye drops may lead to addiction because CLO produce a euphoric effect. As a result of its relaxing and energizing effect, the medication can trigger a desire to repeat procedures when used in large quantities. The frequent use of eye drops may lead to dependency if the individual feels the drops are necessary to achieve the desired results. This addiction can cause adverse effects due to financial pressure, relationship issues, and physical and mental health issues. It is also possible to become addicted to CLO eye drops and suffer serious health problems as a result. Using eye drops excessively and for a prolonged period can lead to eye irritation, blurred vision, and increased sensitivity to light. Severe cornea damage can result in permanent vision loss. Misuse of this medication can also cause heart rate and blood pressure rise, difficulty breathing, and other health problems. A medical professional should monitor the use of drugs to avoid adverse side effects. The use of CLO eye drops should be accompanied by an understanding that CLO may lead to addiction and misuse. The symptoms of CLO eye drop addiction should be addressed, and support sought to overcome the problem and maintain a positive state of mind.

Modern analytical methods, such as UPLC, are extensively employed to analyze multiple drugs in a short time frame [16, 17]. An experiment design strategy based on Box–Behnken design (BBD) was chosen to optimize the chromatographic conditions efficiently. BBD provides a balanced and economical way of evaluating quadratic response surfaces, requiring fewer experiments than central composite design (CCD). A crucial feature of BBD is that it avoids extreme factor levels, which can pose potential risks such as column damage or unstable baselines in chromatographic systems. All experimental points will remain within the practical operating range, thus ensuring a safer and more reliable experiment, which is particularly useful for developing UPLC methods that require precision and system integrity [18–20].

For simultaneous quantification, we established a robust RP-HPLC method that indicates stability. The purpose of this method is to ensure product quality and compliance with regulatory requirements when it comes to assessing drug stability in pharmaceutical formulations. Our proposed HPLC approach has been successfully validated for finding CLO and organic impurities. Temperature, acidity, oxidation, alkalinity, photolysis, neutralization, and humidity all play a role in the process. The goal of this study is to investigate the integration of GAC principles with BBD approaches in order to develop environmentally friendly, efficient, and flexible UPLC methods. Integrating these methods ensures high analytical performance while enhancing their sustainability, particularly when analyzing drugs along with their degradation products simultaneously. Modified green star area (MoGSA), blue applicability grade index (BAGI), and modified green analytical procedure index (GAPI) (MoGAPI) tools were developed to assess the technique's ecological footprint and whiteness. MoGSA is an assessment tool based on work by Mansour et al. (2024) [21], designed to assess chemical reactions and analytical methods' greenness. Through the addition of more detailed criteria relevant to laboratory practices, it modifies the traditional Green Star approach. MoGSA assists in the development of routine pharmaceutical methods by evaluating factors like solvent toxicity and energy consumption. By incorporating WAC principles in addition to the concept of greenness, BAGI extends the concept of greenness. BAGI is therefore particularly relevant for pharmaceutical quality control, in which methods must be not just environmentally friendly, but robust, cost-effective, and suitable for routine use as well [22]. The MoGAPI tool offers a more comprehensive and software-supported evaluation of method greenness than the original GAPI tool, as described by Mansour, Potka-Wasylka, and Locatelli [23]. As it addresses the entire analytical workflow, from sample preparation to detection, it is particularly useful in pharmaceutical laboratories where compliance with regulatory requirements and environmental responsibility are paramount. Every step in the method is visually communicated with a color-coded pictogram in MoGAPI. Compared to previous methods [18–20], our method is more sustainable. Through the substitution of acetonitrile for more sustainable ethanol, the run time has been shortened to 4.0 min, and the power consumption has been reduced. Traditional approaches have been shown to be less cost-effective and less dependable than newly developed technologies (Table S1).

The purpose of this study is to develop a sustainable and efficient RP-UPLC method for detecting CLO, as well as its organic impurities in pharmaceutical formulations, simultaneously. Environmental and regulatory standards for impurity profiling have become increasingly important. Hence, this development is a rationalization for the use of environmentally responsible analytical techniques. The study's aim is to improve method performance and minimize environmental impact by integrating principles of GAC and WAC. In addition to improving operational feasibility, the proposed approach ensures high analytical precision and reduced solvent usage. Ecological footprints and practical applicability are evaluated using tools such as MoGAPI, MoGSA, and BAGI. By using eco-friendly pharmaceutical analysis in routine quality control, this work will contribute to the advancement of green chemistry in pharmaceutical analysis.

## 2. Experimental Section

### 2.1. Chemicals and Reagents

Fisher (Pittsburgh, USA) provided phosphoric acid, bi-distilled water, and ethanol. CLO with 100% potency was obtained from Bioxera Pharma Pvt Ltd. The company is in Mumbai, India. Swixolate 1% eye drops were made by Chemipharm (Cairo, Egypt).

### 2.2. Instruments and Software

AQUITY Arc Systems by Waters provides pressures up to 9500 psi, pH ranges from 1 to 12.5, and flow rates up to 5 mL/minute. The sample paths are stainless steel, carryover is low, there are quaternary pumps, and the PDA detector options are controlled via Empower software.

Design-Expert software (version 13) developed by Stat-Ease, Inc., Minneapolis, MN, USA, was used to analyze BBD data.

### 2.3. Diluent Preparation

Purified water.

### 2.4. Chromatographic System

Chromatography was performed using RP-UPLC in isocratic mode. CORTECS C18 column (50 mm × 2.1 mm, 1.6 μm) was used. Samples were heated to 4°C and columns to 25°C, respectively. Acidic water and ethanol were mixed in a ratio of 65:25, v/v, to prepare the mobile phase. The RP-UPLC method had a 7-min runtime, an injection volume of 0.8 μL, a flow rate of 0.3 mL/min, and UV detection at 254 nm.

### 2.5. Standard Stock Solutions

The concentration of CLO was determined in 50-mL volumetric flasks by preparing standard stock solutions (500 μg/mL). The final volume of the mixture was diluted to approximately 70% by diluent. In addition to sonication, the solutions were diluted with additional diluent for 10 min.

### 2.6. Working Standard Solutions

Transfer 2.0 mL of the stock solution into a 100-mL volumetric flask, add 70 mL of purified water and sonicate until dissolved. Then, use water to make up the volume.

### 2.7. Test Solution

A sample solution for eye drops has been prepared by mixing five bottles. After sonicating the mixture until it dissolves, add enough water to make up the remaining volume. A flask containing 100 μL of sample was filled with 70 mL water, and the rest of the volume was filled with water.

### 2.8. Establishing of Calibration Curves

Diluting the drug stock solutions with purified water to 1–20 μg/mL was performed serially. This range was used to construct the CLO calibration curve.

### 2.9. Procedure

#### 2.9.1. Blank Baseline Subtraction

**Figure 2:**
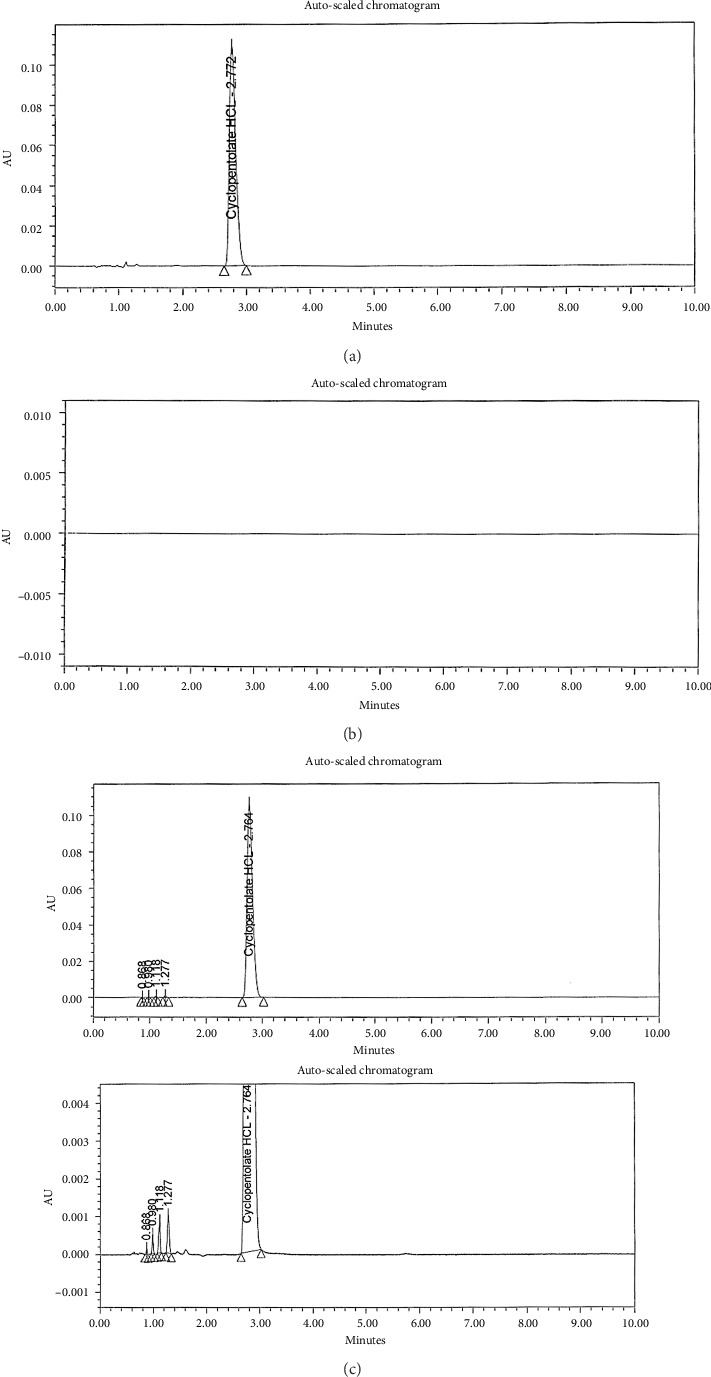
UPLC charts of (a) CLO standard, (b) blank baseline subtraction, and (c) unknown impurities in Swixolate 1% eye drops at 254 nm.

Whether the following types of issues exist with our chromatography should be taken into consideration before proceeding with Empower 3, 3D blank baseline subtraction software: A blank chromatogram includes characteristics that should be removed (such as small noise peaks) because it cannot adequately integrate the standard or sample due to small noise peaks and a drifting noisy baseline. Three-dimensional blank baseline subtraction does not improve the signal-to-noise ratio of the signal despite not changing the blank chromatogram between runs. As a result of blank baseline subtraction, only the background signal is removed, which increases the noise level [24], as described in Figure 2(b).

##### 2.9.1.1. Computations

Calculate any peaks for solvent and placebo based on the processing method to be excluded.

Chromatograms with peaks other than solvent, placebo, and CLO are considered to have unknown impurities.

Based on the following equation, one can calculate how much each impurity is in the CLO portion of the eye drops.

(1) $$\begin{array}{c} \text{Result}=\text{Result}=\left(\frac{r_{U}}{r_{T}}\right)\times 100, \end{array}$$

*r*_*U*_ = peak response of each impurity from the sample solution, *r*_*T*_ = sum of the responses of all of the peaks, excluding the solvent peak, from the sample solution.

## 3. Results and Discussion

### 3.1. Methods Development and Optimization

**Figure 3:**
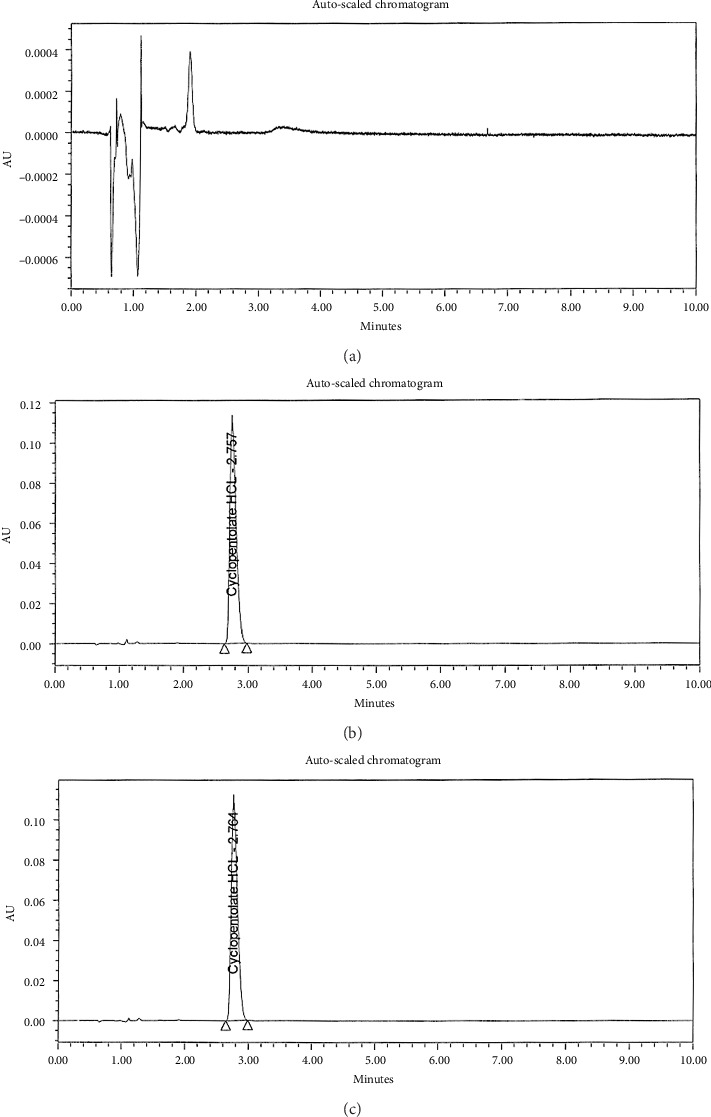
UPLC charts of (a) diluent, (b) CLO standard, and (c) CLO in Swixolate 1% eye drops at 254 nm.

The RP-UPLC method was developed to measure CLO in pure and pharmaceutical formulations by varying the mobile phase composition. A first round of tests was conducted to determine the best conditions for developing an effective pharmaceutical analysis process. For maximum separation and precision, changes were made to the detector wavelength, column temperature, and mobile phase composition. It was possible to manipulate the composition of the mobile phase experimentally through several experiments. In the experiment, water and acetonitrile were diluted 50:50. Following those experiments, a different strategy was devised by mixing acetonitrile with acidic water 50:50. Analyte retention durations were affected by flow rates, pH levels, and mobile phase compositions. Sharp peaks in acidic water, pH 4.25, had well-defined and symmetrical retention durations. It was optimal to mix acidic water and ethanol water and have a flow rate of 0.3 mL/min. As part of the investigation, columns of various lengths and packing materials were utilized. There were three types of columns: C8, C18, and cyano; their lengths ranged from 10 to 50 mm. In this case, CORTECS Premier C18 columns (50 mm × 2.1 mm, 1.6 m) were the best option. The column was heated at 25°C–50°C. Separating drugs requires elevated temperatures. Thus, 4°C and 25°C were recommended for the sample and column oven temperatures, respectively, for optimal separation. A wavelength scan was also carried out between 200 and 400 nm. The following chromatographic parameters were assessed to provide clear, discrete peaks and high resolution: For RP-UPLC, CORTECS Premier C18 columns (50 mm × 2.1 mm, 1.6 m) were utilized. It was adjusted to 4°C for the sample oven and 25°C for the column oven. An RP-UPLC procedure used ethanol and pH 4.25-adjusted acidic water with a duration of 4 minutes, 0.8 μL of injection volume, and 0.3 mL/min flow rate at 254 nm, as shown in Figures 2(a), 2(c), 3.

### 3.2. Risk Assessment

As part of AQbD-based RP-UPLC method development for CLO, Ishikawa (Fishbone) diagrams have been developed as a structured visual tool to identify and categorize potential sources of variance. There are six main causes represented in the diagram: Method Parameters, Instrumentation, Environment, Materials, Analyst, and Measurement. Important factors under Method Parameters are the composition of the mobile phase, the flow rate, the pH of the buffer, the injection volume, and the column temperature of the solution. The risks associated with instrumentation include fluctuations in pump pressure, drift in detectors, column degradation, and software problems. Environmental factors such as temperature fluctuations, humidity, vibrations, and air quality fall under this category. A material's purity includes solvents, buffers, samples, and reagents. Analysts are required to show adherence to protocols, training, experience, and data handling. A measurement error may also be caused by inaccuracies in the preparation of standards and samples or by inconsistencies in integration. As shown in this diagram, the method's robustness is clarified and proactive risk mitigation is encouraged in line with AQbD principles (Figure S1).

### 3.3. RP-UPLC Procedure Optimization by Design of Experiments

As a result of the preliminary screening study, the parameters that influence chromatographic separation were identified. Based on BBD coupled with response surface methodology (RSM), three independent variables at three levels were used to optimize resolution, theoretical plate, and retention time for the two target drugs: pH (4–4.5), ethanol percentage in the mobile phase (60%–70%), column temperature (20°C–30°C), and flow rate (0.25–0.35 mL/min), see Table S2.

The experiment consisted of 29 runs, with retention time, tailing factor, and theoretical plate count as the primary response variables. The system was modeled accurately using a second-order polynomial equation based on the data presented in Table 1. Moreover, three-dimensional response surface plots were generated to visualize interaction between independent variables (Figures S2 and S3). Overlay plots enabled prediction of the most favorable method performance conditions with the help of the desirability function (Figures S4 and S5).

The RSM procedure is a powerful tool for optimizing chromatographic methods. BBD is an innovative design of experiments (DoE) approach that identifies optimal chromatographic parameters efficiently and effectively. Using this approach, one can generate second-order polynomial models to describe system behavior by highlighting the influence of key variables.

BBD is especially useful for method optimization, as it reduces the number of experimental trials significantly. A critical point to note is that BBD does not include experimental points at extreme corners. Consequently, it may not capture the effects of variables at their highest or lowest levels, potentially reducing its predictive accuracy. Even though BBD remains an effective tool for developing methods, it is not suitable for examining responses with extreme values of independent variables [25–28].

### 3.4. Analyses of Statistical Data

An analysis of the statistical data shows a significance of the model and its terms if the *p*-value is less than 0.05. R-squared and adjusted R-squared values of the regression models demonstrated acceptable R-squared and adjusted R-squared values (*R* > 0.8), indicating a good fit to the polynomial equation and supporting the model's predictive capability. This suggests that the experimental data align well with the proposed model.

Further, the model's high R-squared values show its strong forecasting accuracy for the future. It was necessary to perform a lack-of-fit test to fully evaluate the model's suitability. It is important that *p*-values for lack-of-fit should be nonsignificant (*p* > 0.05) to indicate that there is no statistical significance to the deviations between the model and the experimental data.

As shown in Tables 2, 3, and 4, the statistical significance of the model (*p* < 0.05) combined with a nonsignificant lack-of-fit confirms the model's reliability and suitability for predicting the system's behavior.

### 3.5. Variables and Their Effects

#### 3.5.1. Effects of Variables on Resolution

ANOVA tables provide a statistical analysis of the regression model used to predict CLO resolutions. Based on the *p*-value and F-value of 6.54, the regression model is statistically significant, indicating that it reliably explains variation in resolution (Table 2). There is a significant relationship between the ethanol concentration in the mobile phase (A) and the flow rate (B). This study indicates that the amount of ethanol has an effect on chromatographic resolution by a *p*-value of 0.0007 and an *F*-value of 18.62, whereas the flow rate has an even greater impact, with a *p*-value < 0.0001 and an F-value of 65.80. These results highlight the critical role of these two parameters in optimizing chromatographic resolution.

The higher *p*-values (0.4027 and 0.8654, respectively) of column temperature (C) and pH (D) demonstrate that these factors do not significantly affect resolution. Additionally, all interaction terms with *p*-values above 0.05 are statistically nonsignificant, indicating that combinations of variables do not meaningfully influence resolution. In this case, the residual mean square indicates a good fit of the model to the data (0.0280).

(2) $$\begin{array}{c} \text{Resolution}=5.00-0.2083A-0.3917B-0.0417C+0.0083D+0.0750\text{AB}+0.0250\text{AC}+0.0750\text{AD}+0.0500\text{BC}-0.0500\text{BD}-0.0500\text{CD}-0.0750A^{2}-0.1000B^{2}-0.0250C2-0.0750D^{2}. \end{array}$$

In this regression model, various chromatographic parameters are described and how factors interact to influence the resolution of CLO and its impurities. Assuming that all variables are centrally located, the intercept value of 5.00 represents the baseline resolution.

As far as the major effects are concerned, flow rate (B) reduces resolution most pronouncedly (−0.3917) followed by ethanol percentage (A) with a coefficient of −0.2083, and column temperature (C) with a smaller negative impact (−0.0417). Increases in these parameters individually tend to reduce resolution, as indicated by these negative coefficients. However, pH (D) seems to have very little influence on resolution within the studied range (+0.0083).

Variable combinations affect resolution through interaction terms. These combinations produce modest enhancements in resolution when ethanol % is multiplied by flow rate (AB) and by pH (AD) (both +0.0750). A positive interaction can also be observed between ethanol % and temperature (AC) and flow rate and temperature (BC), but their effects are smaller. As opposed to flow rate + pH (BD), temperature + pH (CD) show negative interactions (−0.0500 each), indicating reduced resolution when these factors are increased together.

Quadratic terms (A^2^, B^2^, C^2^, D^2^) all have negative coefficients, with flow rate squared (B^2^) showing the strongest curvature effect (−0.1000). Accordingly, extreme values of flow rate reduce resolution significantly, and optimal performance is likely to be achieved at moderate levels. It appears that very high or very low values of ethanol percentage, temperature, and pH can also negatively impact resolution, though less so.

**Figure 4:**
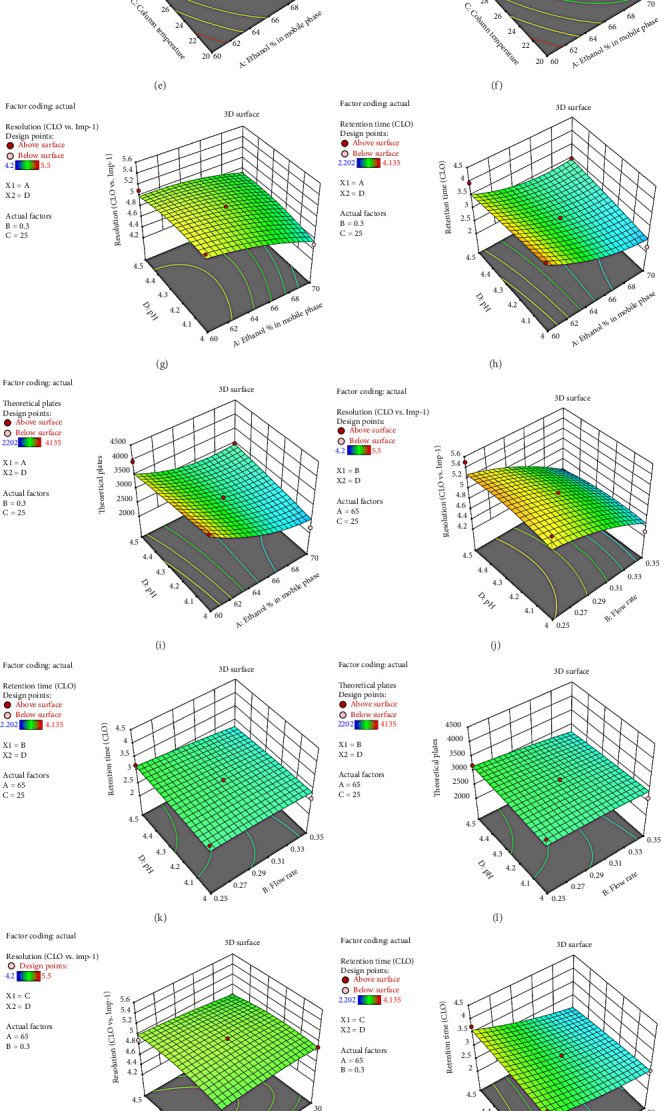
Combined 3D response illustrating the effects of ethanol ratio, flow rate, pH, and column temperature on chromatographic resolution, retention time, and theoretical plates (a–r).

As a result, flow rate and ethanol concentration are the two most influential individual factors, both negatively affecting resolution. Some interactions, especially those involving ethanol percentage, can slightly improve resolution, while others may reduce it. Maintaining optimal resolution also involves avoiding extreme values of the variables (Figures 4(a), 4(b), 4(c), 4(d), 4(e), 4(f), 4(g), 4(h), 4(i), 4(j), 4(k), 4(l), 4(m), 4(n), 4(o), 4(p), 4(q), 4(r)).

#### 3.5.2. Effects of Variables on Retention Time

Analyzing the regression model used to predict CLO retention time is presented in the ANOVA table. Statistical significance is indicated by a model *p*-value of 0.0018 and an F-value of 5.27, indicating that the model can explain CLO retention time variation. As far as the individual factors are concerned, the highest influence is exerted by the ethanol percentage in the mobile phase (A) and the column temperature (C). As a result of ethanol %, there is a highly significant effect (*p* 0.0001, *F* = 35.74), suggesting that ethanol plays a major role in reducing retention periods. In addition to column temperature, *F* = 24.51 is significant (*p* = 0.0002). Higher temperatures may contribute to faster elution (Table 3).

Flow rate (B) and pH (D) have no significant effects on retention time on their own, with *p*-values of 0.1357 and 0.8510, respectively. There is no statistical significance to any interaction term (e.g., AB, AC, AD, etc.), as all have *p*-values well above 0.05, meaning that combinations of variables are not meaningfully influencing retention time.

The quadratic term ethanol % squared (A^2^) is the only statistically significant one (*p* = 0.0334), suggesting an increase in retention time caused by ethanol levels at extreme levels. It is evident that curvature effects on flow rate, temperature, and pH are minimal, as evidenced by their insignificant squared terms.

A low residual mean square (0.0805) indicates a good fit between the model and data.

Overall, ethanol percentage and column temperature influence retention time primarily, with ethanol percentage showing a quadratic effect. As a result of its statistical soundness, the model may be used to predict retention times under different chromatographic conditions.

(3) $$\begin{array}{c} \text{Retention Time }\left(\text{CLO}\right)=74.6947-2.1217A+60.8160B-0.0766C-3.0371D-0.4610\text{AB}+0.0036\text{AC}+0.1660\text{AD}-0.0532\text{BC}-6.2800\text{BD}-0.0972\text{CD}+0.0105A^{2}-9.0400B^{2}+0.0038C^{2}-0.3972D^{2}. \end{array}$$

CLO retention time is predicted by using a regression model that demonstrates how each chromatographic parameter influences the elution behavior. At their central level, all variables have an intercept of 3.00. As a result of increased elution strength, ethanol percentage (A) has the greatest negative influence (−0.4896), suggesting that increasing ethanol content in the mobile phase reduces retention time significantly. The temperature of the column (C) also exhibits a moderately negative effect (−0.4054), suggesting that higher temperatures may slightly speed up the elution process. As for flow rate (B), it has a smaller negative effect (−0.1297), while pH (D) has a minimal positive effect (+0.0157), which suggests that pH has little effect on retention time.

By using interaction terms, the study aims to investigate a deeper understanding of how variables interact to affect retention. A negative coefficient indicates that adding ethanol % to flow rate (AB) results in a slight reduction in retention time (−0.1152). The combination of ethanol % and column temperature (AC) and ethanol % and pH (AD) showed positive effects (+0.0900 and + 0.2075, respectively), suggesting that factors may increase retention time slightly. Other interactions, such as flow rate × pH (BD) and column temperature × pH (CD), also have negative coefficients (−0.0785 and −0.1215), which indicates that these interactions tend to decrease retention time.

In quadratic terms, the response surface exhibits curvature. This finding suggests that extreme ethanol levels may increase retention time as indicated by ethanol % squared (A^2^). On the other hand, flow rate squared (B^2^) and pH squared (D^2^) have a small negative effect (−0.0226 and −0.0248, respectively), indicating a slight decrease in retention time. At higher and lower temperatures, column temperature squared (C^2^) shows a slight positive effect (+0.0953), indicating a mild increase in retention time.

The coefficient −2.1217A indicates that the retention time decreases with an increase in ethanol percentage. Increasing the ethanol content of the mobile phase reduces its polarity, increasing its elution strength, and therefore accelerating the elution of analytes. Moreover, the positive quadratic term +0.0105A2 may suggest a slight nonlinearity, suggesting that the rate of reduction in retention time could taper off at very high ethanol concentrations.

Retention time is most significantly affected by ethanol percentage and column temperature, while interactions and quadratic effects have a more subtle effect. Optimal chromatographic performance can be achieved by carefully balancing these parameters (Figures 4(a), 4(b), 4(c), 4(d), 4(e), 4(f), 4(g), 4(h), 4(i), 4(j), 4(k), 4(l), 4(m), 4(n), 4(o), 4(p), 4(q), 4(r)).

#### 3.5.3. Effects of Variables on Theoretical Plates

Based on the ANOVA table, the regression model used to predict a response variable of theoretical plates was statistically significant, as indicated by the *p*-value of 0.0019 and the F-value of 5.24, demonstrating the effectiveness of the regression model in explaining response variability. There are two independent factors that have the greatest influence: ethanol concentration in the mobile phase (A) and column temperature (C). Among the factors that affected the response, ethanol percentage shows a highly significant (*p* 0.0001, *F* = 35.60). Additionally, column temperature has a significant effect (*p* = 0.0002, *F* = 24.28), suggesting it plays a crucial role in the behavior of the system (Table 4).

A *p*-value of 0.1394 indicates minimal individual influence within the tested range, while 0.8513 indicates minimal individual influence for flow rate (B) and pH (D). There is no significance (*p* > 0.05) for all interactions (e.g., AB, AC, AD), suggesting that the interaction terms do not affect the outcome meaningfully.

The only statistically significant quadratic term is ethanol % squared (A^2^) (*p* = 0.0334), which indicates a curvature effect—extreme values of ethanol % may cause responses to change nonlinearly. In the studied range, flow rate, temperature, and pH have no significant squared terms, suggesting linear effects.

Accordingly, the residual mean square is relatively low (80,801.70), indicating that the model fits the data reasonably well.

Ultimately, the model shows statistical validity, with the most critical factors being the ethanol percentage and column temperature. It is important to control this parameter carefully for optimizing the response since ethanol % has a significant quadratic effect. Variables and interactions appear to have limited influence on the results.

(4) $$\begin{array}{c} \text{Theoretical Plates}=3000.00-489.58A-128.58B-404.33C+15.67D-115.25\text{AB}+90.00\text{AC}+207.50\text{AD}-16.50\text{BC}-78.50\text{BD}-121.50\text{CD}+263.33A^{2}-23.67B2+94.21C^{2}-24.29D^{2}. \end{array}$$

A regression model describes the relationship between various chromatographic parameters and how factors interact to determine the theoretical plate number, a key indicator of the efficiency of a column. As the central level of all variables is at 3000.00, the intercept value represents the baseline number of theoretical plates.

A significant negative impact has been observed for ethanol content (A) (−489.58), showing that increasing ethanol content significantly reduces column efficiency. A strong negative correlation exists between column temperature (C) and separation performance (−404.33), indicating that higher temperatures may lead to poorer separation efficiency. Flow rate (B) is less negative (−128.58), while pH (D) is slightly positive (+15.67), indicating minimal benefits for theoretical plates.

Relationships are revealed through interaction terms. With the highest interaction (+207.50), ethanol percentage together with pH (AD) can dramatically improve column efficiency. There is also a positive interaction between ethanol percent and temperature (AC) and ethanol percent and pH (AD). The relationship between ethanol percentage and flow rate (AB), flow rate and pH (BD), and temperature and pH (CD), however, has negative coefficients, which implies that these combinations reduce theoretical plate numbers.

As a result of the quadratic terms, the response surface is curvy. There is a pronounced positive trend in ethanol % squared (A^2^), suggesting that extreme ethanol levels may actually improve efficiency after a certain point. In the same way, column temperature squared (C2) shows a similar trend (+94.21). There is, however, a negative relationship between flow rate (B^2^) and pH (D^2^), indicating that these variables can reduce column efficiency when their values are either very high or very low.

A combination of ethanol percentage and column temperature appears to be the most influential individual factor, both having a negative effect on theoretical plates. It is important to note, however, that their squared terms and certain interactions (particularly with pH) have the potential to offset these effects under certain circumstances. As a result of this model, it becomes evident that balancing these parameters is crucial to optimizing chromatographic performance (Figures 4(a), 4(b), 4(c), 4(d), 4(e), 4(f), 4(g), 4(h), 4(i), 4(j), 4(k), 4(l), 4(m), 4(n), 4(o), 4(p), 4(q), 4(r)).

### 3.6. MoGSA Tool

Using the MoGSA index, chemical processes are evaluated according to their ecological consequences, including the use of solvents, reagents, energy, and waste. An analysis of the differences between different methods was carried out using MoGSA. Green star area indexes (GSAIs) often lack clear boundaries between environmentally friendly and nonsustainable practices, and the 12 green chemistry tenets apply differently in different environments. Open-source software can be found at https://bit.ly/MOGSA. Metrics related to green chemistry fall into three categories: mass metric, environmental health hazards, and computation tools. The metrics are calculated and visualized using software and spreadsheets, assisting in a more precise assessment of green chemistry. There are several comprehensive metrics available, but GSAI and EcoScale are the two most popular. In EcoScale, penalty points are used to calculate a numerical score between 0 and 100. Subtract 100 from the EcoScale score after assigning penalties to each element [21]. GSAI represents greenness through a green star whose area is proportional to how well it complies with green chemistry principles. In addition, it assesses how well the 12 principles of green chemistry have been implemented. Every GSAI principle has a point that represents how much the principle has been adhered to, with the length of the point indicating the extent of compliance. Health hazards, environmental risks, recyclability of resources, and product degradation all play a role in measuring this metric. Green areas indicate higher ecological impact, while larger green areas indicate higher greenness. Chemists can compare different methods quickly and easily with GSAI, allowing improvements towards more sustainable practices to be made more quickly. In addition to being particularly useful in educational settings, it promotes the use of green chemistry principles in a wide range of industrial settings.

**Figure 5:**
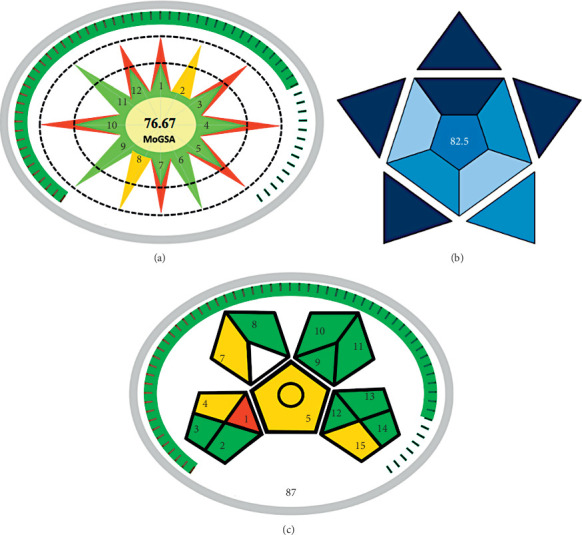
(a) MoGSA, (b) BAGI, and (c) MoGAPI tools for appraisal sustainability of UPLC technique.

As shown in Figure 5(a), this method scored 76.67 on the MoGSA index, indicating an essential degree of compatibility with the environment. This study also demonstrated a correlation between GSA scores and the nature of materials (Figure S6a).

### 3.7. BAGI Tool

BAGI is a metric for estimating a chromatography method's “blueness” or practicality based on its analytical “blueness.” Free software is available at http://mostwiedzy.pl/pl/justyna-plotka-wasylka,647762-1/BAG I. Rather than being an alternative to green metrics, the BAGI metric takes inspiration from the RGB model. By appraisal the technique utilizing BAGI, an asteroid chart is produced with a number around the center. Pictogram scales reflect how closely a method adheres to designated criteria based on their hue. A color-coding system is used for BAGI, where dark blue indicates high compliance, blue indicates medium compliance, light blue indicates low compliance, and white indicates noncompliance. Approximately 25–100 points are given to the analytical method in the pictogram's center (Figure S6b). One hundred points are awarded to methods with excellent application, and 25 points are awarded to methods with poor application. Practical techniques are those whose BAGI score is at least 60 [22]. The inner part of the pictogram contains five criteria, which represent the stages of analytical evaluation and sample preparation. A group of criteria 6–10 is placed at the end of the outer part to cover both stages. A shade representing the average shade is applied to the result field based on all BAGI criteria.

Using BAGI, analytical procedures are evaluated based on 10 practical attributes. A summary of the results of the BAGI assessment is shown in Figure 5(b). Among the six assessed methods, six received high scores, exceeding 60, which indicates their practicality. This assessment gave the highest score of 82.5.

### 3.8. MoGAPI Tool

Analytic methodology steps can be evaluated on their greenness using the GAPI. Each phase of the chemical analysis process is represented by a pentagram divided into subsections labeled green, yellow, or red depending on its greenness. With this GAPI tool, you can quickly determine how stressful the procedure is for the environment and how safe it is. The GAPI metric, however, cannot be used to calculate total scores to allow comparisons between methods. The current GAPI metric has limitations, which have been addressed by the development of a MoGAPI and software. Greenness can be assessed more precisely with the presented tool, while its use can be simplified and accelerated with the software. Also, it integrates the advantages of the Eco-Scale and GAPI metrics in a single model (Figure S6c). The current study was successfully evaluated using this tool to demonstrate its applicability. Furthermore, the MoGAPI tool's software is available free of charge (open source) at http://bit.ly/MoGAPI for comparing methods and applications. Aside from providing researchers with a reliable and easy way to evaluate analytical techniques, this tool and its software also represent a significant advance in greenness assessment [23]. Based on the proposed and developed evolution in the current GAPI scale, these methods can be compared as well as classified into excellent, acceptable, and inadequate green, like the score used in the analytical Eco-Scale. In this MoGAPI assessment, the total score is displayed on the chart, and the color around the pentagrams indicates how the method is ranked overall. In Figure 5, the presented method achieves a total score of 87, with a greater number of green sections.

### 3.9. Greenness and Sustainability Assessment

Sustainable metrics such as MoGAPI, MoGSA, and BAGI have gained traction as comprehensive tools for evaluating environmental impact and practical functionality of analytical methods in recent years. Even though these tools are relatively new, their quantitative frameworks have been validated across multiple analytical platforms, including UPLC, as demonstrated by benchmark studies [21, 23]. In these studies, the metrics are reproducible and can be used to distinguish between greener and less sustainable methods. By integrating GAPI with the Analytical Eco-Scale, for instance, MOGAPI provides a more nuanced method of scoring, while BAGI evaluates method feasibility to complement greenness assessments [22]. Through MoGSA, selective applications of green chemistry principles can be made, enhancing flexibility and relevance across a wide range of applications. While further harmonization is needed to ensure universal adoption, these tools provide a robust and reproducible framework for sustainability assessment, aligning with current best practices in GAC. An evaluation of the environmental and practical performance of the developed RP-UPLC method was conducted using three structured sustainability assessment tools: MoGAPI, MoGSA, and BAGI [21–23]. Each tool provides a unique framework for assessing analytical methods. Using GAPI and the Analytical Eco-Scale, MoGAPI produces a score from 0 to 100, with values of 75 indicating excellent greenness, 50–74 acceptable, and 50 inadequate. A score of > 85 is considered highly green, 60–85 moderately green, and 60 lowly green by MoGSA. With BAGI, method practicality is assessed across 10 weighted attributes, including automation, sample throughput, and reagent use, on a scale of 0–100, where scores of 75 indicate high applicability. Combined, these tools provided a multidimensional assessment of the sustainability of the method.  Table S3 presents a comparison of the scoring systems, thresholds, and references of these methods to facilitate comparisons.

### 3.10. Limitations and Future Directions of the Study

Although the RP-UPLC method was found to be highly selective in distinguishing CLO from its organic impurities, a comprehensive forced degradation study was not carried out in this study. Due to this, its capability to resolve potential degradants under stress conditions has yet to be evaluated. As a result, future studies should be planned to include forced degradation experiments to further validate the stability-indicating potential of the method and ensure its robustness at various stages of degradation. This study used Empower's 3D blank baseline subtraction to improve chromatographic clarity and reduce background interference. In addition to introducing variability in signal-to-noise ratios, this approach may also reduce quantification sensitivity. Moreover, due to the lack of certified impurity standards, area normalization was used to quantify impurities without applying relative response factor (RRF) corrections. The present study has limitations in these areas. In spite of the fact that the BBD used in this study was effective in optimizing the RP-UPLC method, and statistically significant models for resolution, retention time, and theoretical plates were generated, differences in the effect of certain variables were observed. Overall, flow rate (B) and pH (D) had relatively minor effects on chromatographic performance. As an example, pH coefficients for the resolution model were minimal (+0.0083) and for the theoretical plates model were minimal (+15.67), indicating limited impact under the conditions tested. A narrow experimental range or inherent stability of the analyte under slight pH and flow rate variations may explain this. Due to the limitations of the current design, these results highlight a limitation in its capability to capture all of the potential influences of these parameters. In future studies, pH and flow rate could be expanded or additional factors could be incorporated, such as buffer type, column dimensions, or gradient elution. The method's robustness and applicability could be further enhanced by incorporating more complex matrix effects or samples with real-world variability, in addition to the four variables currently considered. In addition to enhancing the generalizability of the method, exploring these aspects can provide deeper insights into its effectiveness under a wide range of analysis conditions. In future studies, alternative solvents could be explored as well as extending the application of this approach to other anticholinergic or ophthalmic medications to enhance the method's green profile. Developing this method in this way would enhance its utility and contribute to the advancement of sustainable pharmaceutical practice in the future.

## 4. Method Validation

Optimization and validation of the UPLC method were based on ICH standards [29].

### 4.1. Linearity and Range

Analyte concentrations are directly correlated with the data obtained using the linear method. The peak area and concentrated form of CLO have significant correlations.  Table 5 provides detailed information about the characterization of calibration curves.

### 4.2. LOD and LOQ

The verified sheet uses equations (3.3*σ*/S) and (10*σ*/S), respectively, to estimate LOD and LOQ. Calculating LOD and LOQ by using the intercept's standard deviation and the calibration curve's slope is highly sensitive.  Table 5 shows lower values for LOQ and LOD, which results in higher effectiveness for the suggested approaches.

### 4.3. Precision

#### 4.3.1. Repeatability

To test repeatability, six replicates of a known concentration were tested for each drug. Across six replicates, the relative standard deviation (RSD) for the system was less than 2.0% (Table 5).

#### 4.3.2. Reproducibility

The ruggedness of a laboratory is evident under different laboratory conditions, on different days, with different analysts and equipment.  Table S4 shows that the results were good.

### 4.4. Accuracy and Recovery

The study focuses on the evaluation of the accuracy of the method using a regression equation using percent recoveries at three concentrations crossing the linearity curve. Based on Table 6, testing drug samples according to the suggested procedures yields results between 98% and 102%.

### 4.5. Robustness

A small, deliberate variation in method parameters such as flow rate, pH, column temperature, and ethanol percentage was assessed on retention time and peak area. As a result of the method's consistent performance, %RSD values remained below 2%, proving its reliability under a number of different analytical circumstances, see Table 7 and Table S5.

### 4.6. Standard Solution Stability

As a result of the experiments, it is evident that the standard solution is of high quality. In comparison to the previously prepared solution, the recently prepared solution showed excellent agreement after 72 h at room temperature and in the refrigerator. The previously stored standard also fits well with the newly made one, with a range of 98%–102% and an RSD of less than 2.0. It is clear from these findings that the standard solution has excellent stability and reliability, making it an excellent choice for any analytical application, as outlined in Table S5.

### 4.7. System Suitability

This technique is used to determine the system's suitability by deciding parameters. This includes tailing factors, peak resolutions, retention periods, and theoretical plate counts. The analytical techniques used in these innovative tests meet the requirements for system appropriateness as outlined by the ICH and USP. At least 2000 theoretical plates must be used to measure column efficiency. In addition, a minimum resolution of 1.5 is required between CLO and IMP. As shown in Table 8, a tailing factor of more than 2 is not recommended. Compliance with these factors is crucial in determining whether a system is appropriate for its intended purpose.

### 4.8. Specificity

#### 4.8.1. Selectivity

As the retention times of the target compounds were observed, no interference was observed between the analytes and excipients. This enhances the method's reliability and suitability for routine quality control. An analysis of the interactions between excipients, coating agents, and solvent was needed to assess the specificity of the approach. Neither active component is significantly different when diluted with diluents, placebos, or other compounds, as shown in Figure 3(a). Selectivity can be achieved with these techniques.

### 4.9. Assay of Dosage Form and Related Test

Three samples were analyzed. Swixolate 1% eye drops was successfully tested for CLO and impurities after their duplicate preparation. This is seated in Table S6. Based on Student's t-tests and F-tests at a 95% confidence level, there were no significant differences between this method and one previously reported. When evaluating precision, the *t*-test compares variances, while the F-test compares means. The results indicate that the proposed method can accurately and precisely analyze these drugs in their dosage form (Table S6).

## 5. Conclusion

The study concludes by demonstrating that green and white chemistry can significantly improve sustainability and efficiency. RP-UPLC was evaluated using three eco-friendly assessment tools including MoGAPI, MoGSA, and BAGI for its environmental impact and practicality. This method was developed for the analysis of CLO and its organic impurities. These tools included MoGAPI, MoGSA, and BAGI. CORTECS premier C18 columns were used in isocratic mode with ethanol-buffer mobile phase and specific temperature settings as part of this procedure. Using this method, anticholinergic drugs and their impurities can be quantified simultaneously. A blank subtraction method uses PDA software to calculate impurities faster, thus reducing wasted time. By following ICH guidelines, this approach had been validated to be environmentally sustainable using green and white metrics.

## Figures and Tables

**Table 1:** Critical factors and analytical responses in the Box–Behnken design of a UPLC method.

Std	Run	Factor 1	Factor 2	Factor 3	Factor 4	Response 1	Response 2	Response 3
		A: ethanol % in mobile phase	B: flow rate	C: column temperature	D: pH	Resolution (CLO vs. Imp-1)	Retention time (CLO)	Theoretical plates
								
18	1	70	0.3	20	4.25	4.8	3.21	3210
15	2	65	0.25	30	4.25	5.1	2.953	2953
6	3	65	0.3	30	4	4.9	2.764	2764
1	4	60	0.25	25	4.25	5.3	3.542	3542
26	5	65	0.3	25	4.25	5	3	3000
12	6	70	0.3	25	4.5	4.7	2.988	2988
14	7	65	0.35	20	4.25	4.6	3.316	3316
13	8	65	0.25	20	4.25	5.2	3.4208	3408
3	9	60	0.35	25	4.25	4.5	3.871	3871
4	10	70	0.35	25	4.25	4.4	2.831	2831
16	11	65	0.35	30	4.25	4.7	2.795	2795
27	12	65	0.3	25	4.25	5	3	3000
5	13	65	0.3	20	4	4.8	3.61	3610
28	14	65	0.3	25	4.25	5	3	3000
19	15	60	0.3	30	4.25	5	3.106	3106
7	16	65	0.3	20	4.5	4.9	3.743	3743
11	17	60	0.3	25	4.5	5.1	3.956	3956
22	18	65	0.35	25	4	4.3	2.632	2632
25	19	65	0.3	25	4.25	5	3	3000
9	20	60	0.3	25	4	5.2	4	4000
20	21	70	0.3	30	4.25	4.6	2.541	2541
8	22	65	0.3	30	4.5	4.8	2.411	2411
29	23	65	0.3	25	4.25	5	3	3000
21	24	65	0.25	25	4	5.4	3.22	3220
24	25	65	0.35	25	4.5	4.2	2.308	2308
17	26	60	0.3	20	4.25	5.3	4.135	4135
10	27	70	0.3	25	4	4.5	2.202	2202
23	28	65	0.25	25	4.5	5.5	3.21	3210
2	29	70	0.25	25	4.25	4.9	2.963	2963

**Table 2:** Statistical evaluation of resolution (CLO vs. Imp) using quadratic ANOVA.

**Source**	**Sum of squares**	**df**	**Mean square**	***F*-value**	**p** **value**	

Model	2.56	14	0.1831	6.54	0.0006	Significant
A—ethanol % in mobile phase	0.5208	1	0.5208	18.62	0.0007	
B—flow rate	1.84	1	1.84	65.80	< 0.0001	
C—column temperature	0.0208	1	0.0208	0.7447	0.4027	
D—pH	0.0008	1	0.0008	0.0298	0.8654	
AB	0.0225	1	0.0225	0.8043	0.3850	
AC	0.0025	1	0.0025	0.0894	0.7694	
AD	0.0225	1	0.0225	0.8043	0.3850	
BC	0.0100	1	0.0100	0.3574	0.5595	
BD	0.0100	1	0.0100	0.3574	0.5595	
CD	0.0100	1	0.0100	0.3574	0.5595	
A^2^	0.0365	1	0.0365	1.30	0.2726	
B^2^	0.0649	1	0.0649	2.32	0.1501	
C^2^	0.0041	1	0.0041	0.1449	0.7092	
D^2^	0.0365	1	0.0365	1.30	0.2726	
Residual	0.3917	14	0.0280			
Lack of fit	0.3917	10	0.0392			
Pure error	0.0000	4	0.0000			
Cor total	2.95	28				

**Table 3:** Analysis of variance for retention time response in UPLC method optimization.

**Source**	**Sum of squares**	**df**	**Mean square**	***F*-value**	**p** **value**	

Model	5.94	14	0.4240	5.27	0.0018	Significant
A—ethanol % in mobile phase	2.88	1	2.88	35.74	< 0.0001	
B—flow rate	0.2017	1	0.2017	2.51	0.1357	
C—column temperature	1.97	1	1.97	24.51	0.0002	
D—pH	0.0029	1	0.0029	0.0366	0.8510	
AB	0.0531	1	0.0531	0.6602	0.4301	
AC	0.0324	1	0.0324	0.4026	0.5360	
AD	0.1722	1	0.1722	2.14	0.1656	
BC	0.0007	1	0.0007	0.0088	0.9266	
BD	0.0246	1	0.0246	0.3063	0.5887	
CD	0.0590	1	0.0590	0.7337	0.4061	
A^2^	0.4480	1	0.4480	5.57	0.0334	
B^2^	0.0033	1	0.0033	0.0412	0.8421	
C^2^	0.0589	1	0.0589	0.7316	0.4068	
D^2^	0.0040	1	0.0040	0.0497	0.8269	
Residual	1.13	14	0.0805			
Lack of fit	1.13	10	0.1127			
Pure error	0.0000	4	0.0000			
Cor total	7.06	28				

**Table 4:** Evaluating resolution response using ANOVA in a quadratic UPLC model.

**Source**	**Sum of squares**	**df**	**Mean square**	***F*-value**	**p** **value**	

Model	5.924E + 06	14	4.232E + 05	5.24	0.0019	Significant
A—ethanol % in mobile phase	2.876E + 06	1	2.876E + 06	35.60	< 0.0001	
B—flow rate	1.984E + 05	1	1.984E + 05	2.46	0.1394	
C—column temperature	1.962E + 06	1	1.962E + 06	24.28	0.0002	
D—pH	2945.33	1	2945.33	0.0365	0.8513	
AB	53,130.25	1	53,130.25	0.6575	0.4310	
AC	32,400.00	1	32,400.00	0.4010	0.5368	
AD	1.722E + 05	1	1.722E + 05	2.13	0.1664	
BC	1089.00	1	1089.00	0.0135	0.9092	
BD	24,649.00	1	24,649.00	0.3051	0.5894	
CD	59,049.00	1	59,049.00	0.7308	0.4070	
A^2^	4.498E + 05	1	4.498E + 05	5.57	0.0334	
B^2^	3633.15	1	3633.15	0.0450	0.8351	
C^2^	57,568.93	1	57,568.93	0.7125	0.4128	
D^2^	3827.58	1	3827.58	0.0474	0.8308	
Residual	1.131E + 06	14	80,801.70			
Lack of fit	1.131E + 06	10	1.131E + 05			
Pure error	0.0000	4	0.0000			
Cor total	7.056E + 06	28				

**Table 5:** The regression statistics of CLO employing UPLC approach.

Parameter	Drug
	CLO
Wavelength	254 nm
Range (μg/mL)	2–20
Coefficients of determination (R^2^)	0.9999
Slope (b)	3660.6972
RSD of the slope (Sb%)	0.08
Intercept (a)	−45.8099
S_a_	44.7824
S_b_	3.5293
S_y/x_	43.9684
Significance F	6.7 × 10^−13^
LOD^a^	0.04 μg/mL
LOQ^a^	0.12 μg/mL

^a^Limit of detection (3.3 × σ/slope) and a limit of quantitation (10 × σ/slope).

**Table 6:** Accuracy and recovery results of the suggested UPLC method.

CLO		
Test (%)	St add.n (mL) to 10-mL flask	Recovery (%)
40%	4	100.20
60%	6	99.33
80%	8	99.88
100%	10	100.47
160%	16	99.56
Minimum		99.33
Maximum		100.47
Average		99.89
SD		0.46
RSD%		0.46

**Table 7:** Evaluation of the suggested method to multiple variations.

Analyte	Chromatographic parameters	Wavelength (nm)		Ethanol ratio		Flow rate	
CLO		252	256	67.00%	69.00%	0.2 min/mL	0.4 min/mL
	Assay %	102.4%	104.7%	99.2%	100.1%	100.7%	101.3%
	Retention time (Rt)	2.72	2.77	2.73	2.70	2.82	2.65
	Tailing factor	1.02	1.03	1.01	1.07	1.06	1.08
	Resolution	2.80	2.85	2.78	2.83	2.75	2.62

**Table 8:** System suitability items of the recommended UPLC approach.

Parameters	Results	Limit
Precision	0.2%	RSD ≤ 2.0%
USP tailing factors	1.1	NMT 2.0%
Retention time variability	0.7%	≤ ±10%
Theoretical plates (plate count)	4603	≥ 2000
Resolution	4.2	≥ 1.5

## Data Availability

The data that support the findings of this study are available in the supporting information of this article.

## Supporting Information

Figure S1: Ishikawa (Fishbone) diagram for risk assessment in AQbD-based RP-UPLC method development for CLO.

Figure S2: Combined response surface plots and diagnostic visualizations illustrating the effects of ethanol ratio, flow rate, pH, and column temperature on chromatographic resolution, retention time, and theoretical plates.

Figure S3: A 3D plot with response surfaces annotated with optimal points.

Figure S4: Predicting optimal method conditions using desirability functions (a–f).

Figure S5: Integrating overlay plots and desirability functions for method performance optimization (a–g).

Figure S6: Appraisal tools of (a) MoGSA, (b) BAGI, and (c) MoGAPI for the recommended UPLC method.

Table S1: Analysis of CLO drugs using the proposed method in comparison with the existing methods.

Table S2: Transformation of raw data to coded levels.

Table S3: Comparative overview of sustainability assessment tools.

Table S4: Results of intraday and interday precision for CLO in dosage form.

Table S5: Robustness evaluation of the developed RP-UPLC method for CLO under small deliberate variations in analytical parameters.

Table S6: Statistical analysis of the suggested UPLC and the reported technique for CLO quantification in its dosage form.
