# Supplementary material for: Green RP-UPLC Method for Simultaneous Determination of Cyclopentolate and Organic Impurities Using DoE and Sustainability Metrics
Source: Int J Anal Chem. 2025 Sep 16;2025:8827373. doi: 10.1155/ianc/8827373 (PMC12457066; doi:10.1155/ianc/8827373)
Supplement: Supporting Information — Additional supporting information can be found online in the Supporting Information section. [file 8827373.f1.docx]

**Green RP-UPLC Method for Simultaneous Determination of Cyclopentolate and Organic Impurities Using DoE and Sustainability Metrics**

**Bandar R. Alsehli ^1^, Abdullah H. Alluhayb ^2^, Lateefa A. Al-Khateeb^3^, Sayed M. Saleh ^2^, Ammena Y. Binsaleh ^4^**, **and Mahmoud A. Mohamed ^5*^**

^1^ Department of Chemistry, Faculty of Science, Taibah University, Al-Madinah Almunawrah 30002, Saudi Arabia; [bshle@taibahu.edu.sa](mailto:bshle@taibahu.edu.sa)

^2^ Department of Chemistry, College of Science, Qassim University, Buraidah 51452, Saudi Arabia; [ah.alluhayb@qu.edu.sa](mailto:ah.alluhayb@qu.edu.sa) ; [e.saleh@qu.edu.sa](mailto:e.saleh@qu.edu.sa)

^3^ Chemistry Department, Faculty of Science, King Abdulaziz University, P.O Box 80200-Jeddah 21589, Saudi Arabia; [laalkhatib@kau.edu.sa](mailto:laalkhatib@kau.edu.sa)

**^4^** Department of Pharmacy Practice, College of Pharmacy, Princess Nourah bint Abdulrahman University, P.O. Box 84428, Riyadh 11671, Saudi Arabia; [Aysaleh@pnu.edu.sa](mailto:Aysaleh@pnu.edu.sa)

^*5^ Hikma Pharmaceutical Company, Beni-Suef 62511, Egypt.

^c^*Corresponding author E-mail: [ch.mahmoud88@gmail.com](mailto:ch.mahmoud88@gmail.com), [mmabdelfatah@hikma.com](mailto:mmabdelfatah@hikma.com), Tel. +2 01124767625

The ORCID of the corresponding author: 0000-0003-3946-5465

**Abstract**

A significant improvement in sustainability and efficiency is achievable through green and white chemistry. As part of this study, sustainability assessment tools were used to assess the environmental impact and practicality of an innovative, straightforward RP-UPLC method to analyze cyclopentolate (CLO) and its organic impurities simultaneously in pure and ophthalmic solutions at the same time. An optimization strategy based on Box-Benken was employed to minimize experimental runs while optimizing chromatographic conditions. Using this design, four critical variables were evaluated comprehensively—ethanol percentage in the mobile phase, pH, column temperature, and flow rate—on chromatographic responses such as retention time, resolution between CLO and impurity, and theoretical plate count. As a result of desirable and overlay plots, an optimal condition was selected: 65:25, v/v, ethanol and buffer, pH 4.25, 0.3 mL/min flow rate, and 4°C and 25°C sample and column oven temperatures, and the main peak retained for a little more than three minutes. The calibration curves for CLO and impurities at concentrations from 5-50 µg/mL and 1-20 µg/mL showed a correlation value of 0.9998. Recoveries are ±15% of the actual amounts, which is acceptable. RP-UPLC has been extensively designed for the coincidental estimation of anticholinergic drugs and their impurities. A combination of white and green tools was used to assess the method's environmental impact. ICH guidelines have been followed to validate the suggested strategy. This approach offers a reliable, fast, and eco-friendly solution for routine pharmaceutical quality control of anticholinergic agents.

**Keywords:** Cyclopentolate; RP-UPLC; Organic Impurities; Box-Benken; Sustainability assessment tools.

**Figure S1.** Ishikawa (Fishbone) Diagram for risk assessment in AQbD-based RP-UPLC method development for CLO.


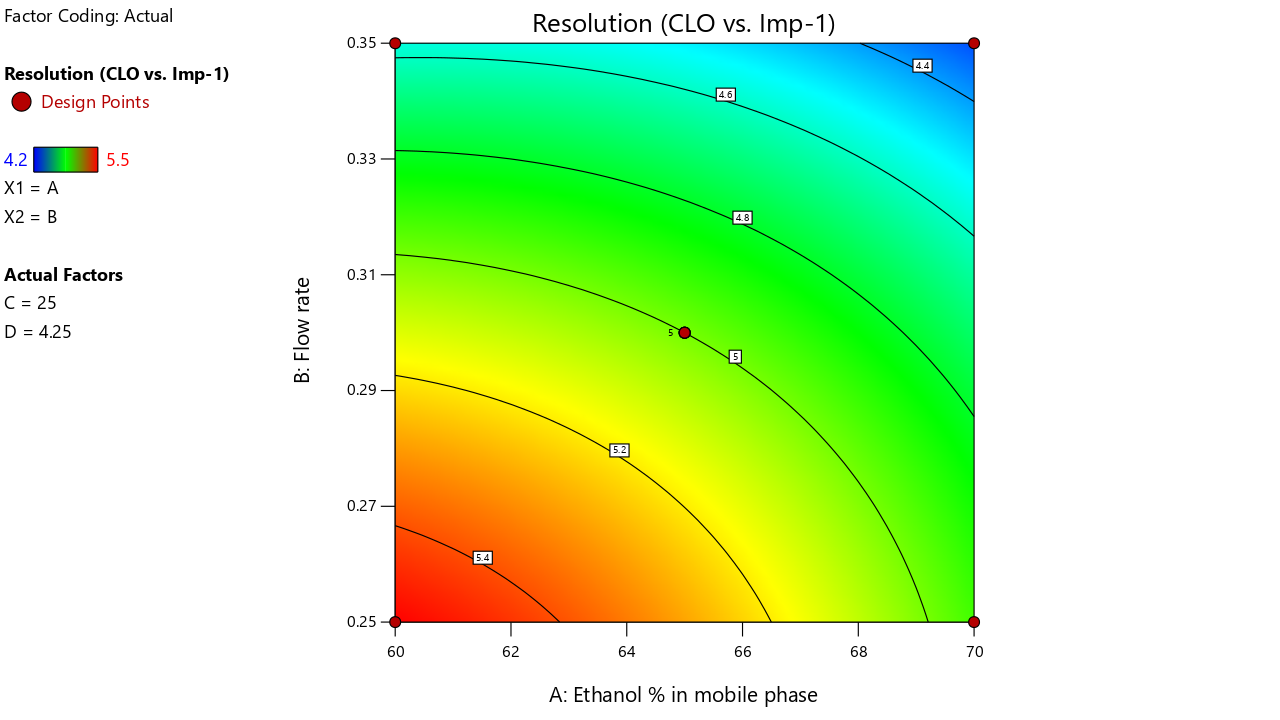


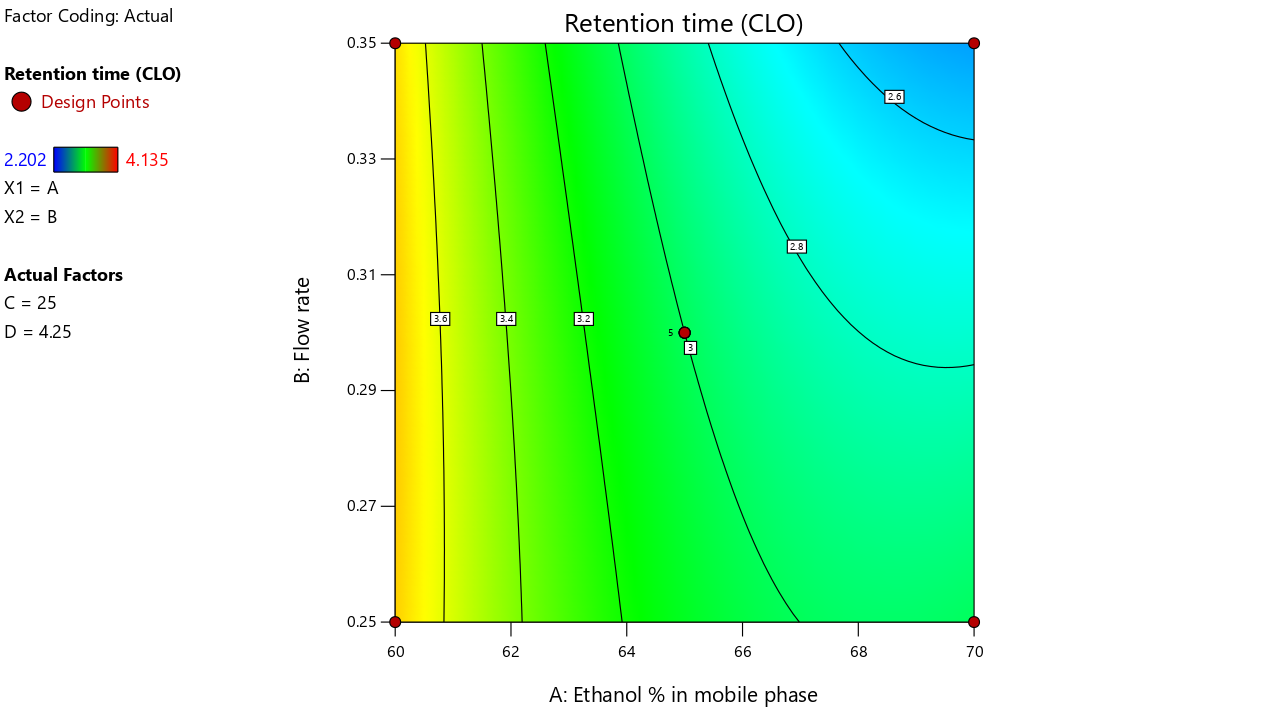


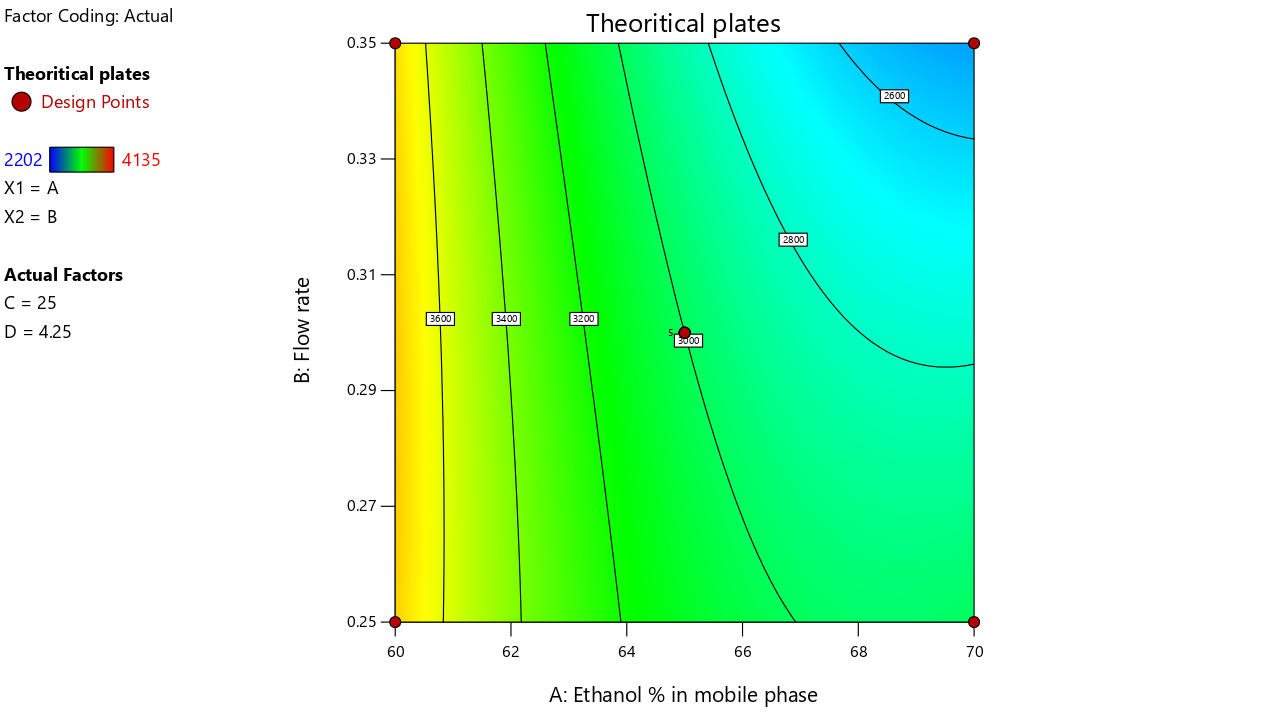


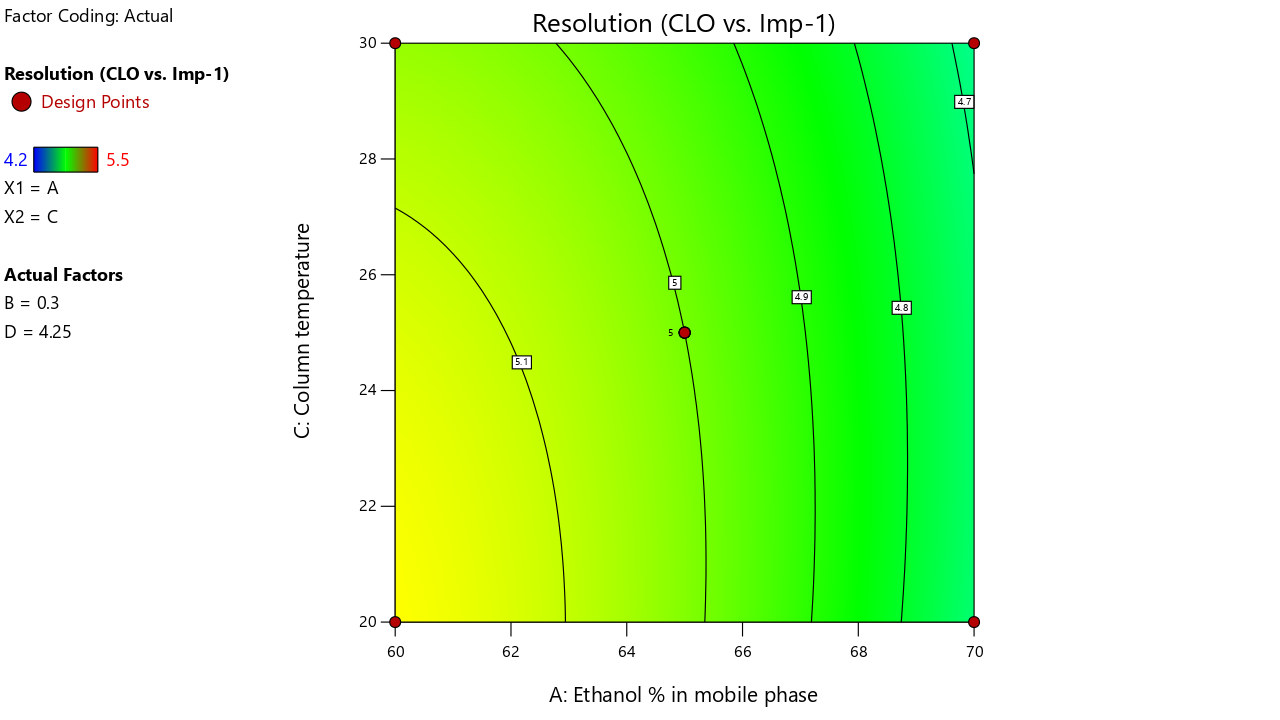


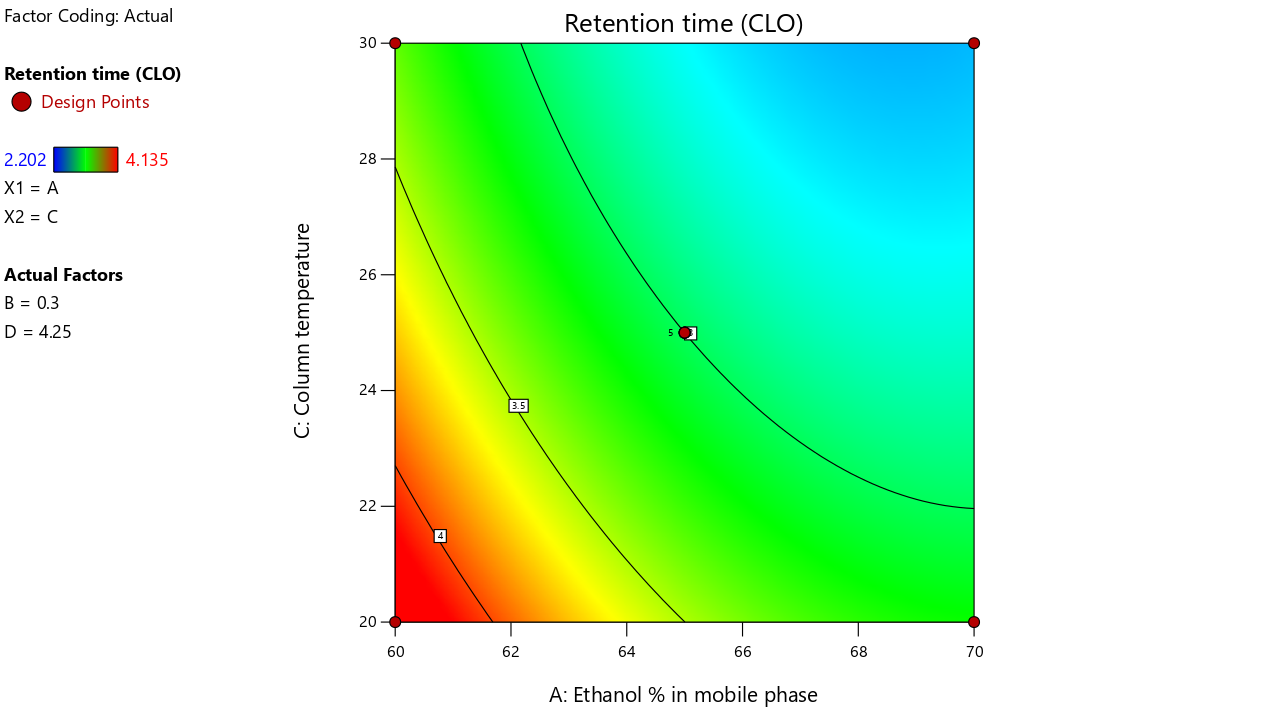


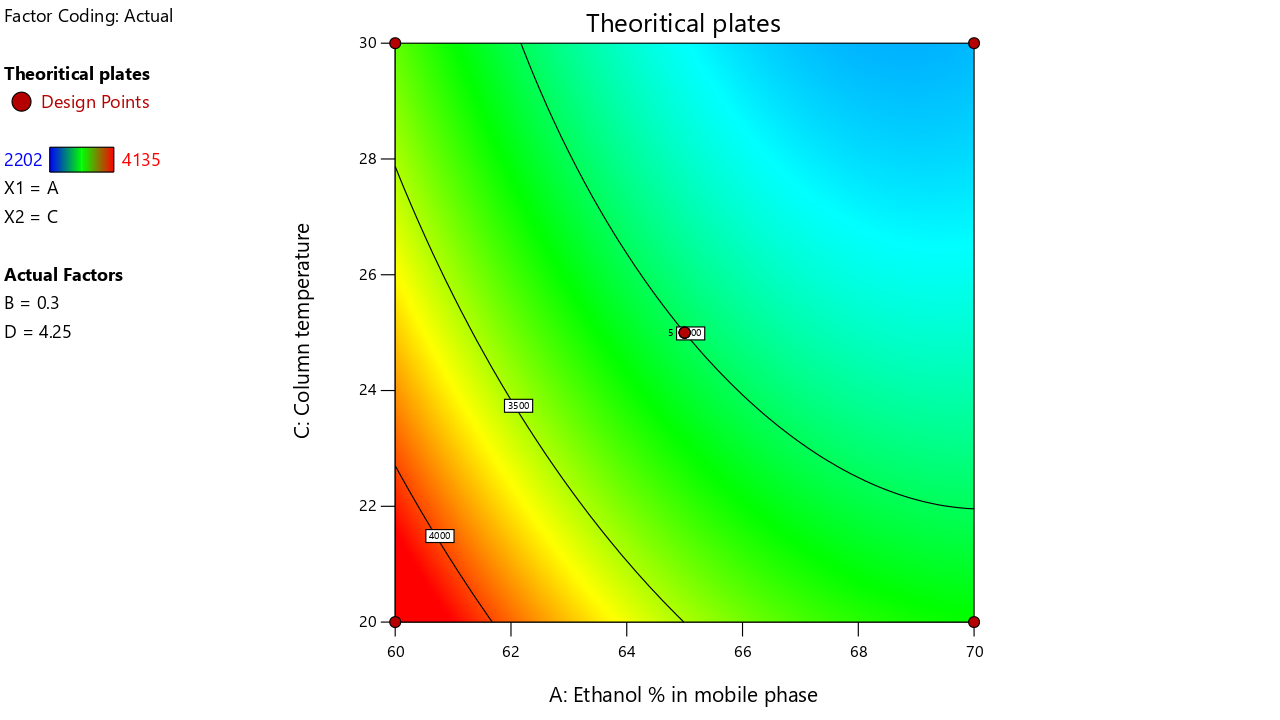


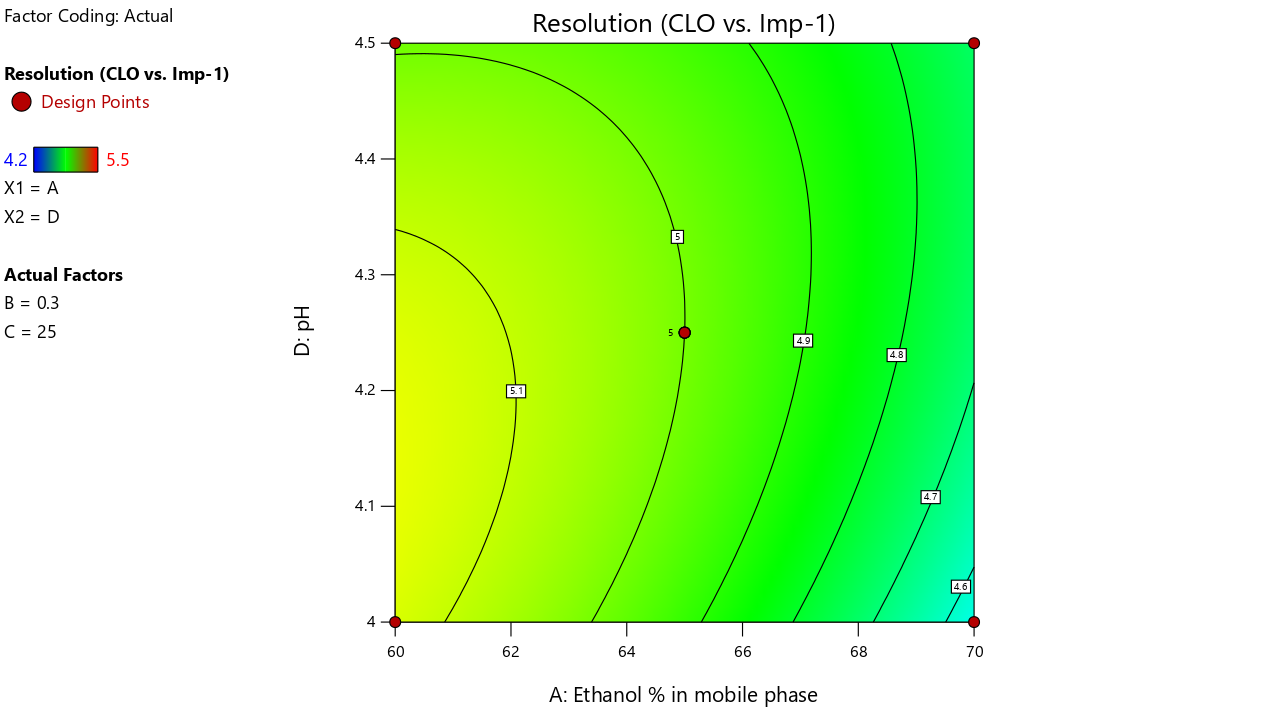


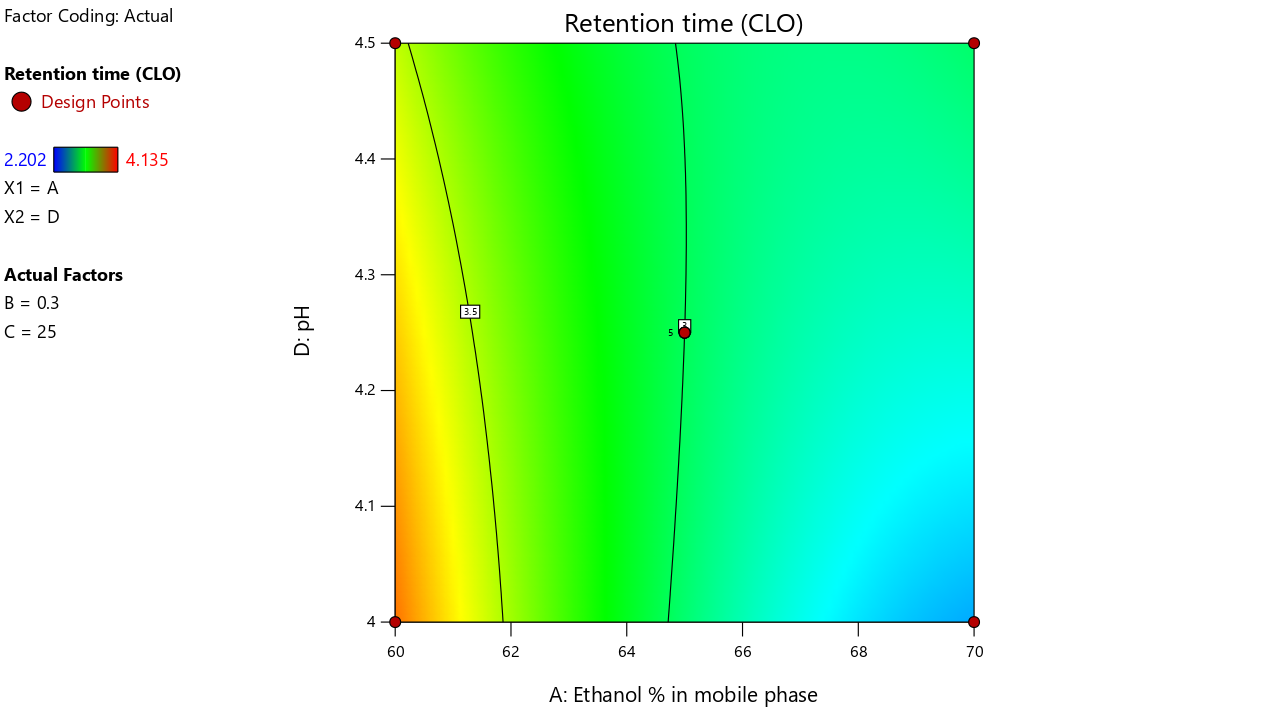


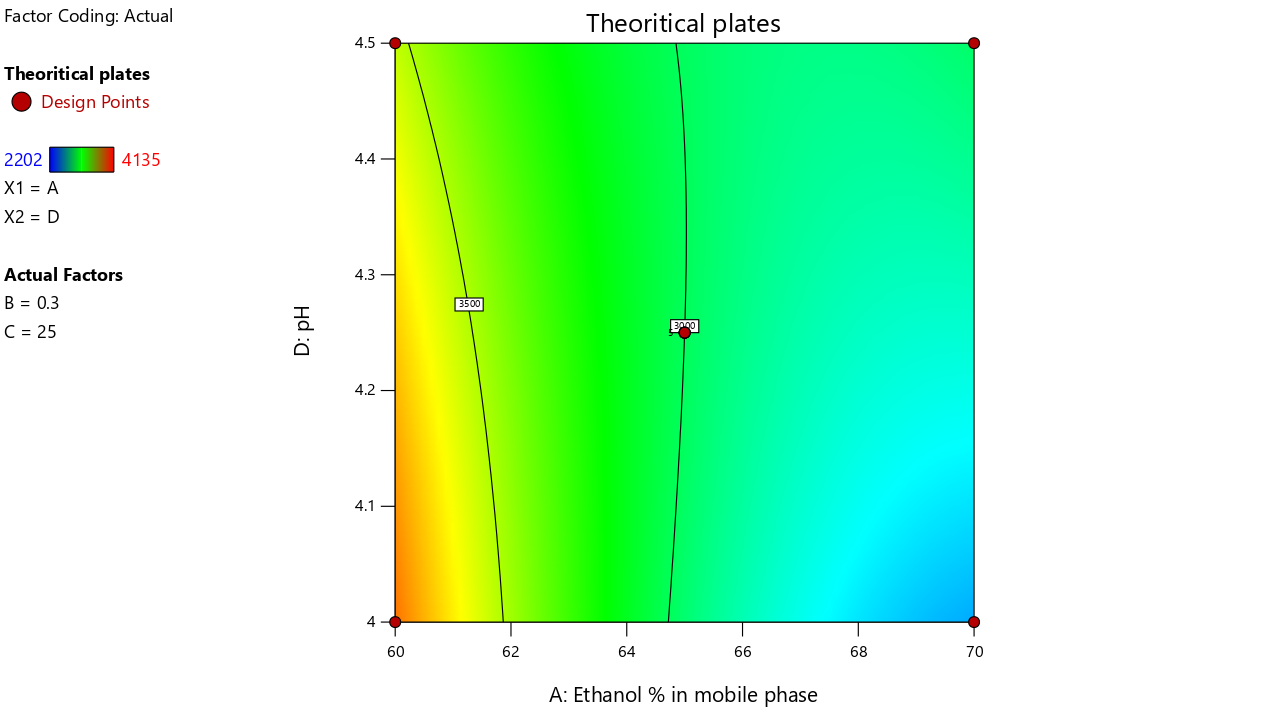


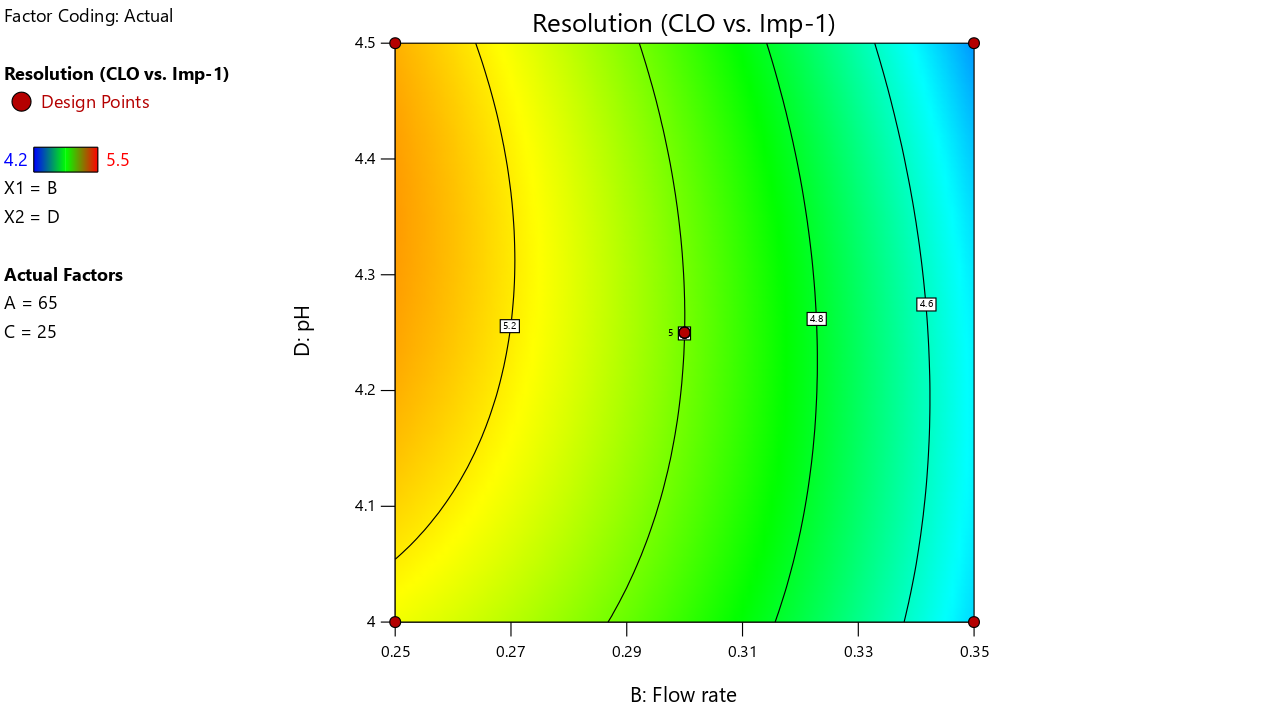


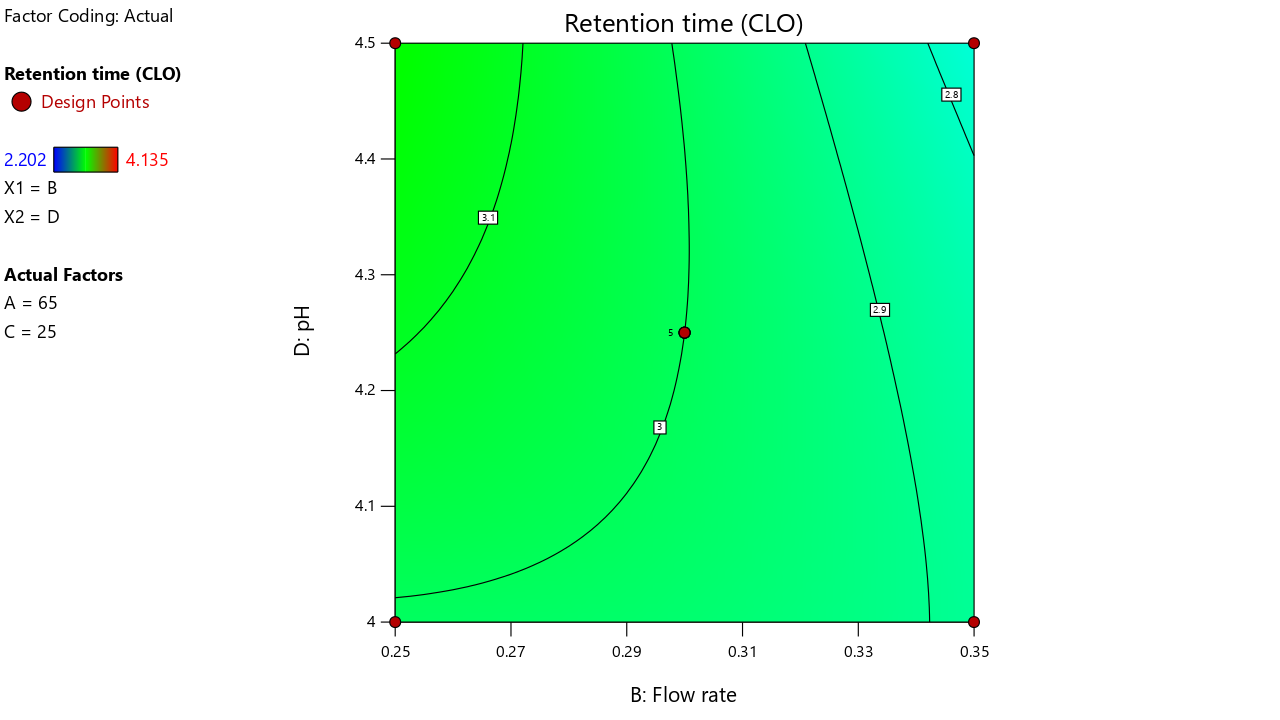


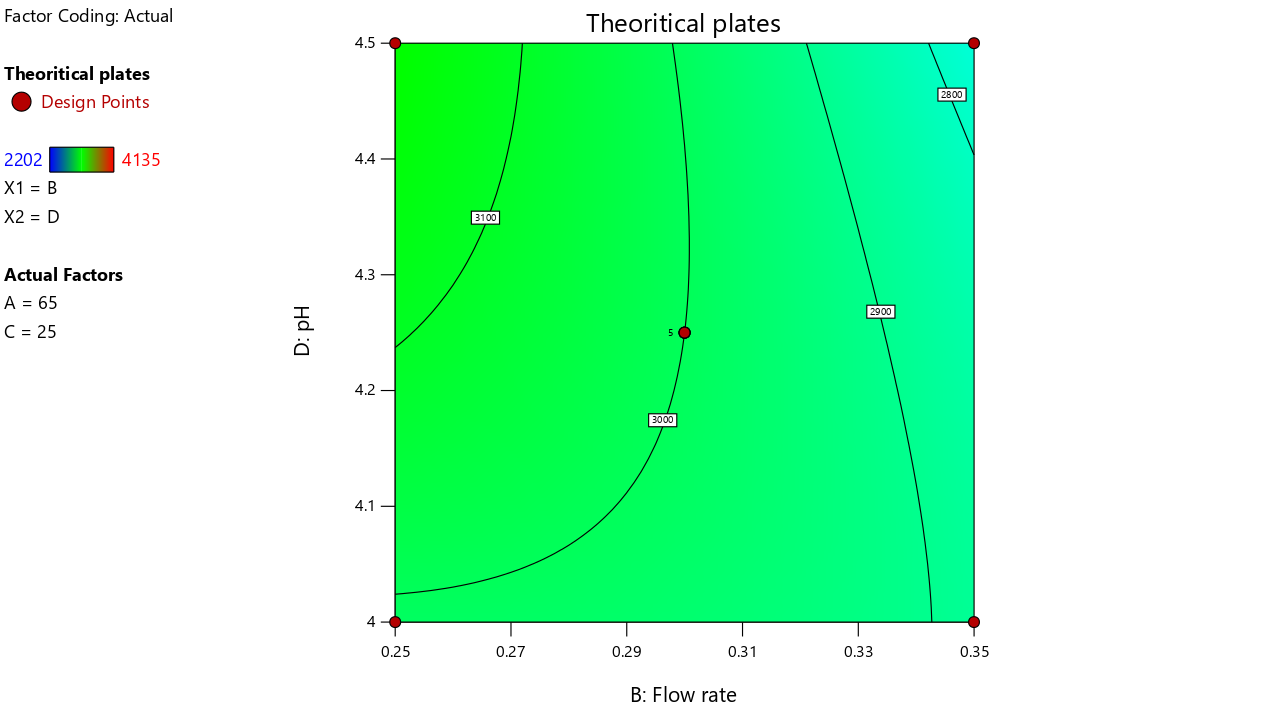


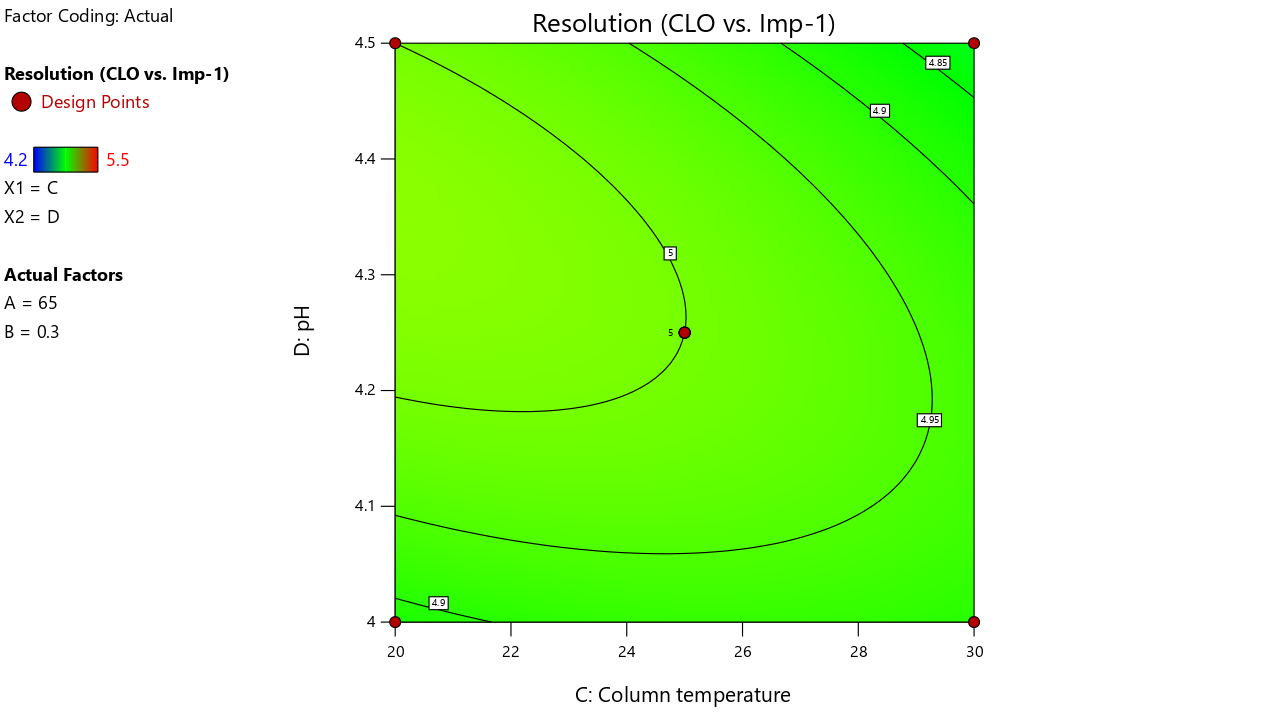


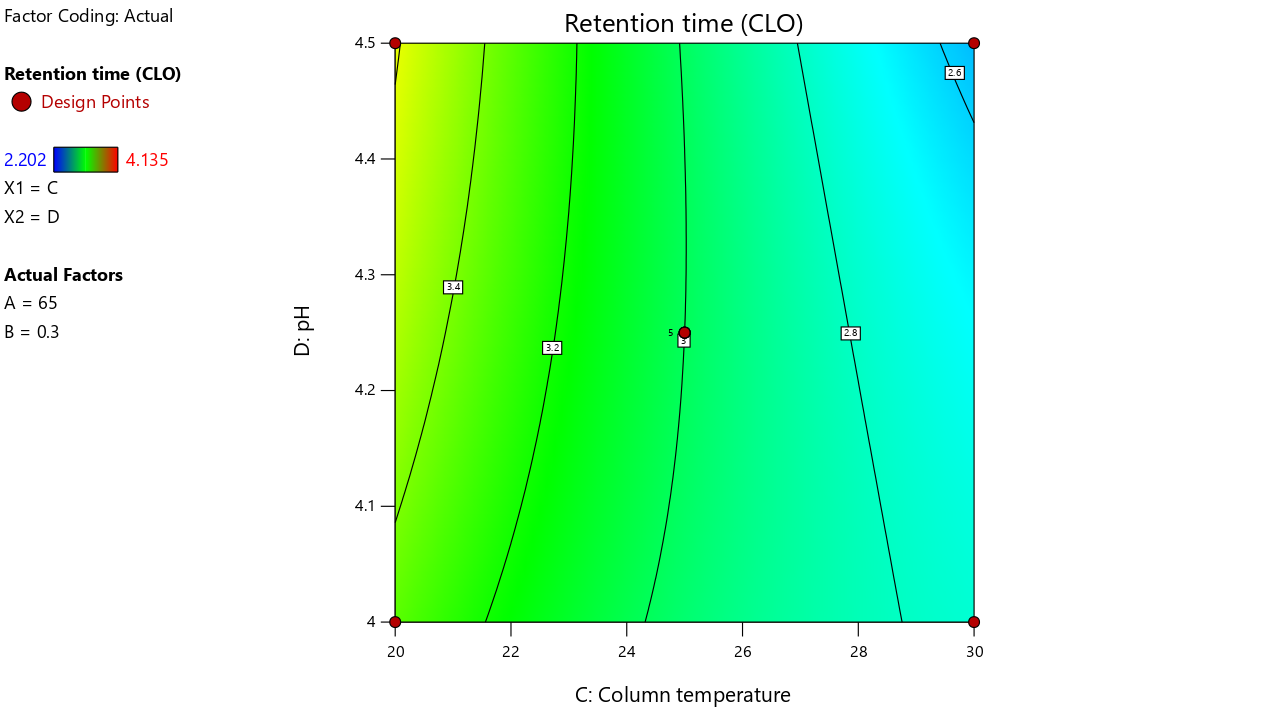


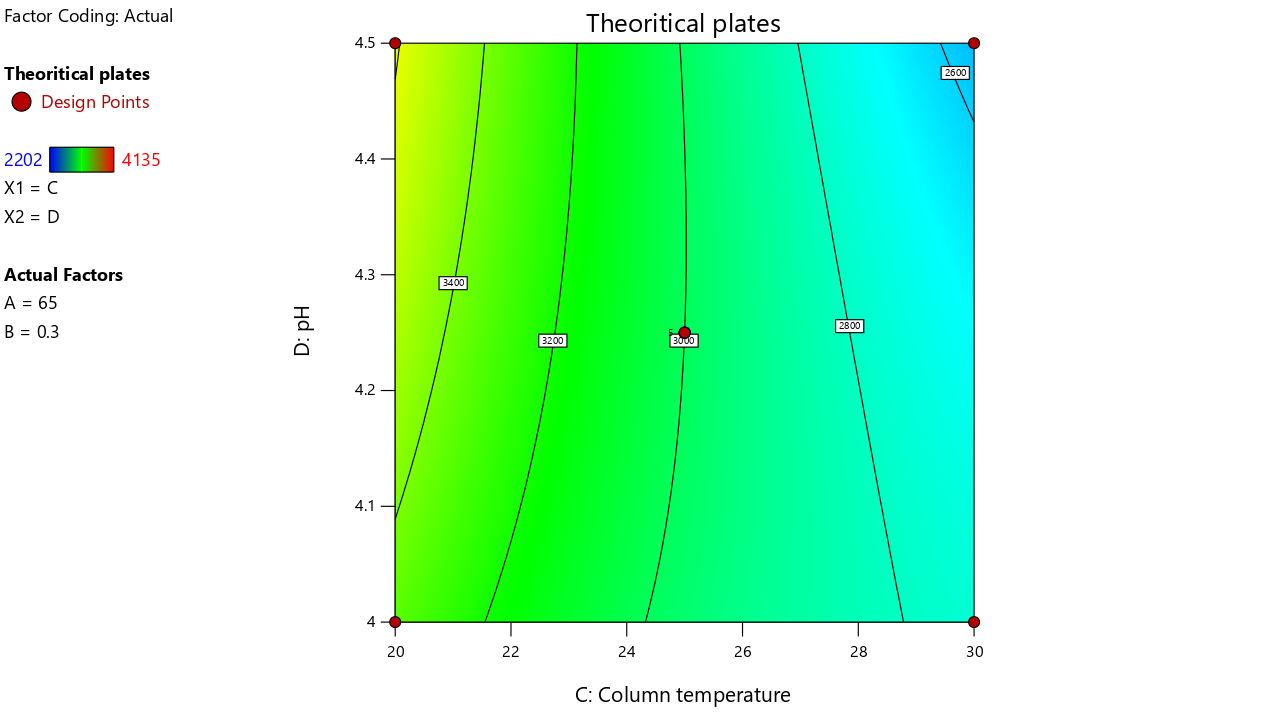


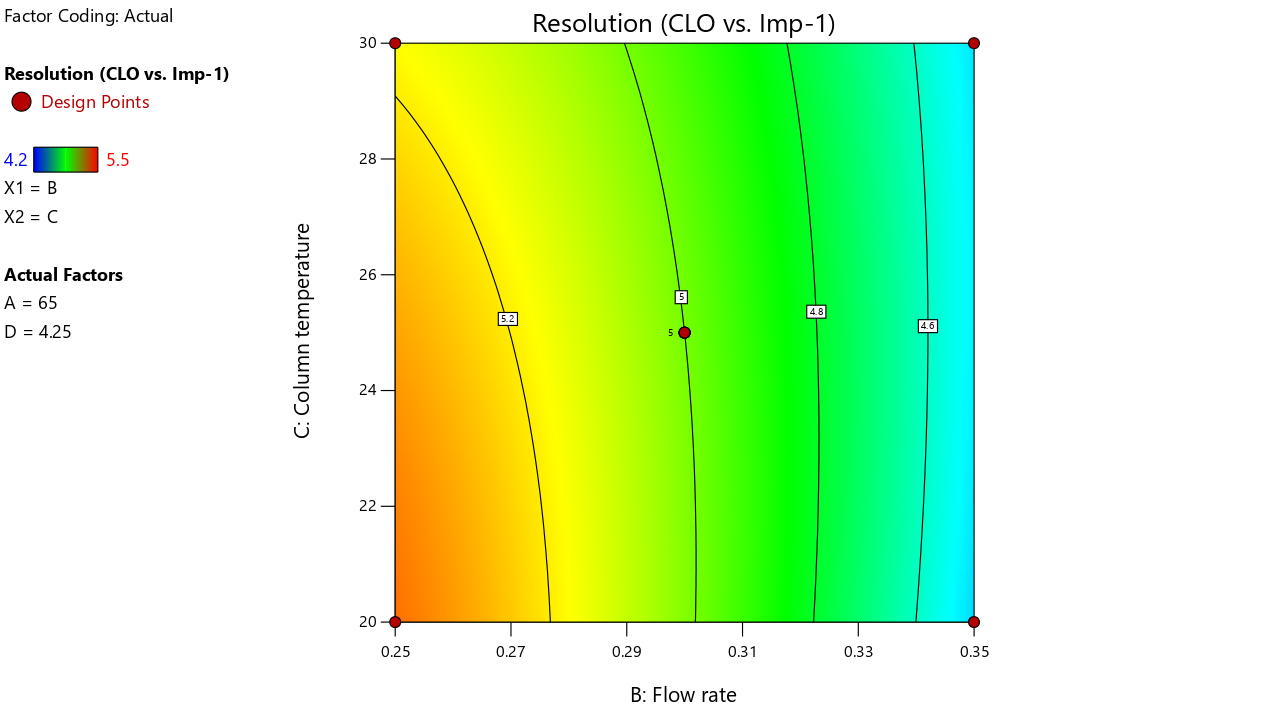


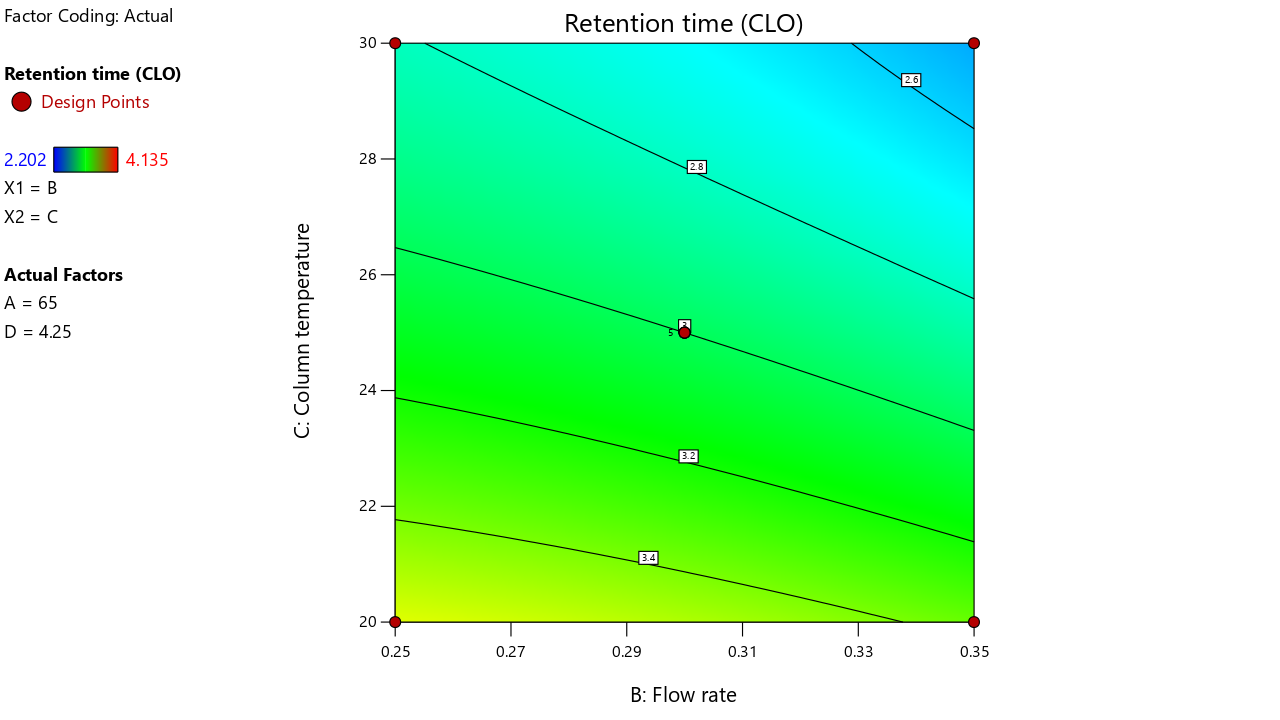


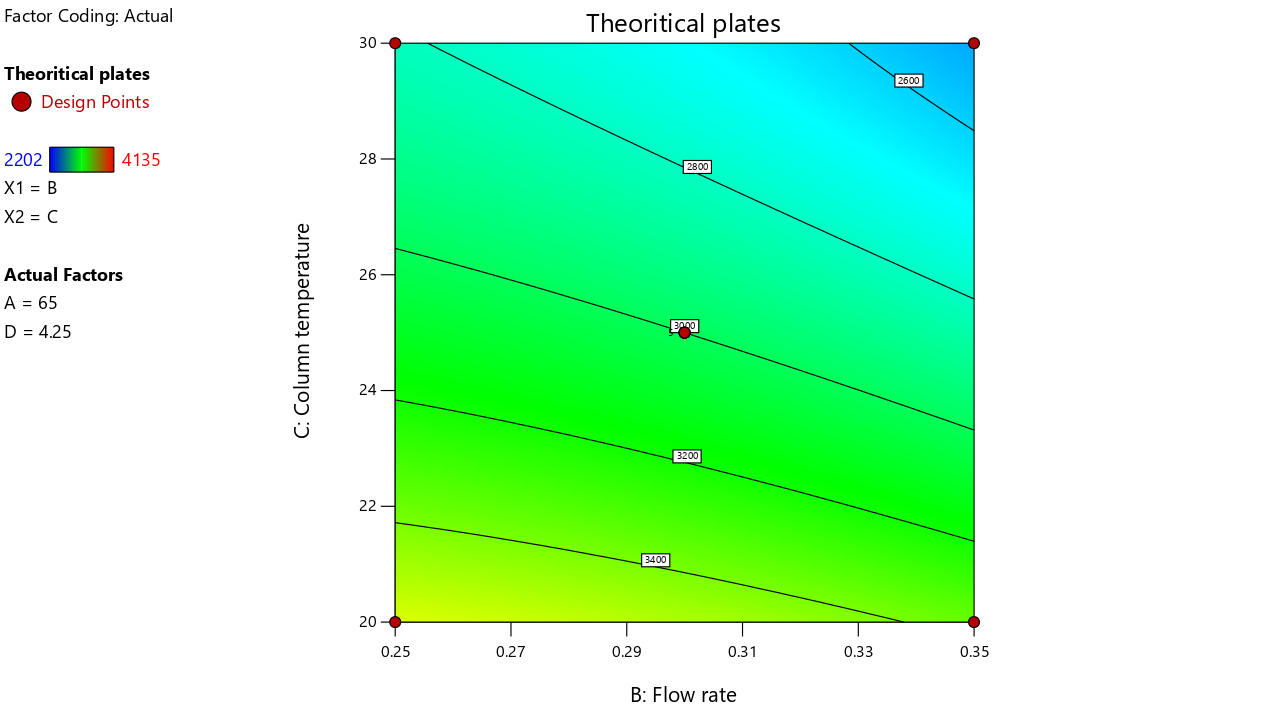


**Figure S2.** Combined response surface plots and diagnostic visualizations illustrating the effects of ethanol ratio, flow rate, pH, and column temperature on chromatographic resolution, retention time, and theoretical plates.


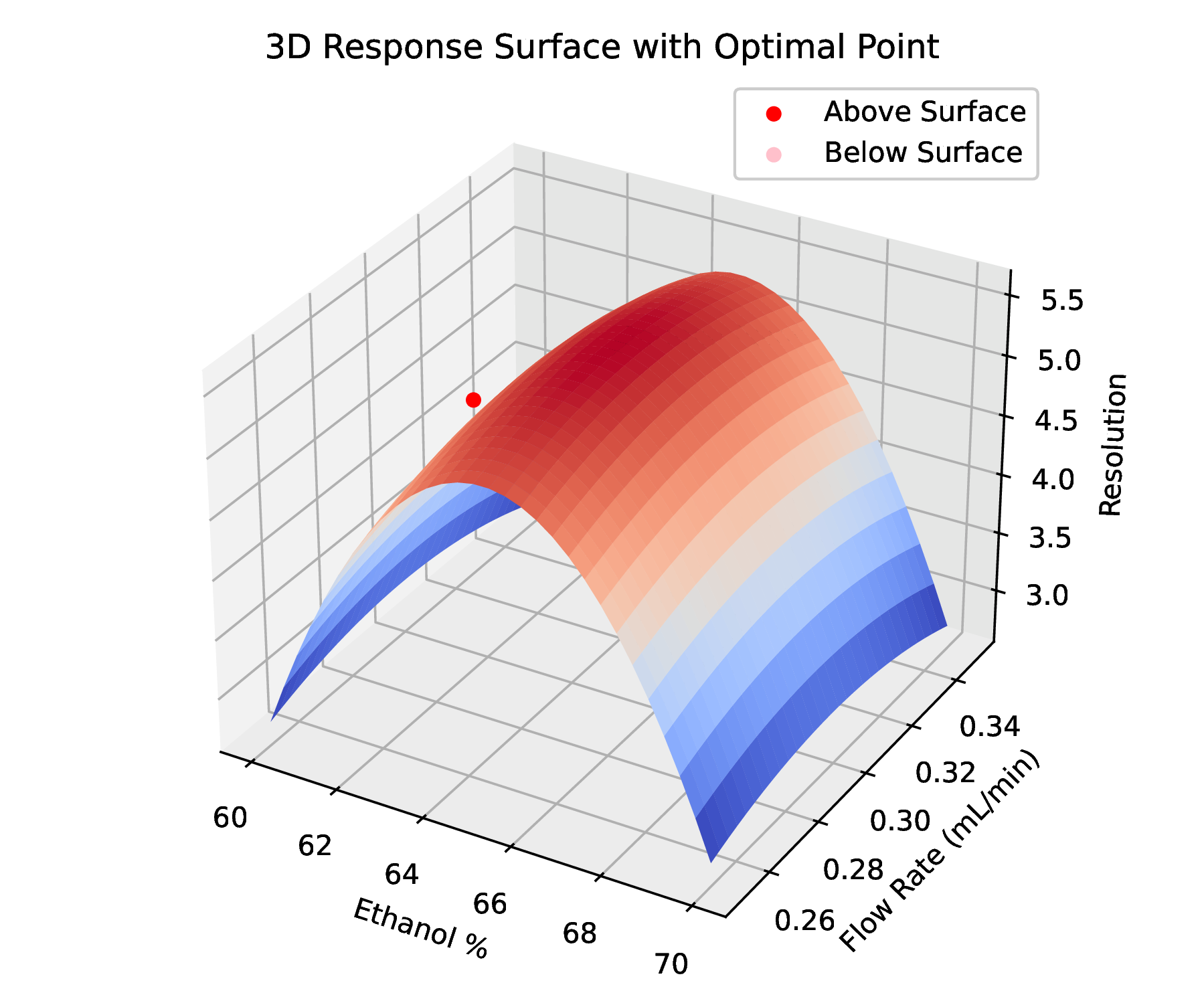


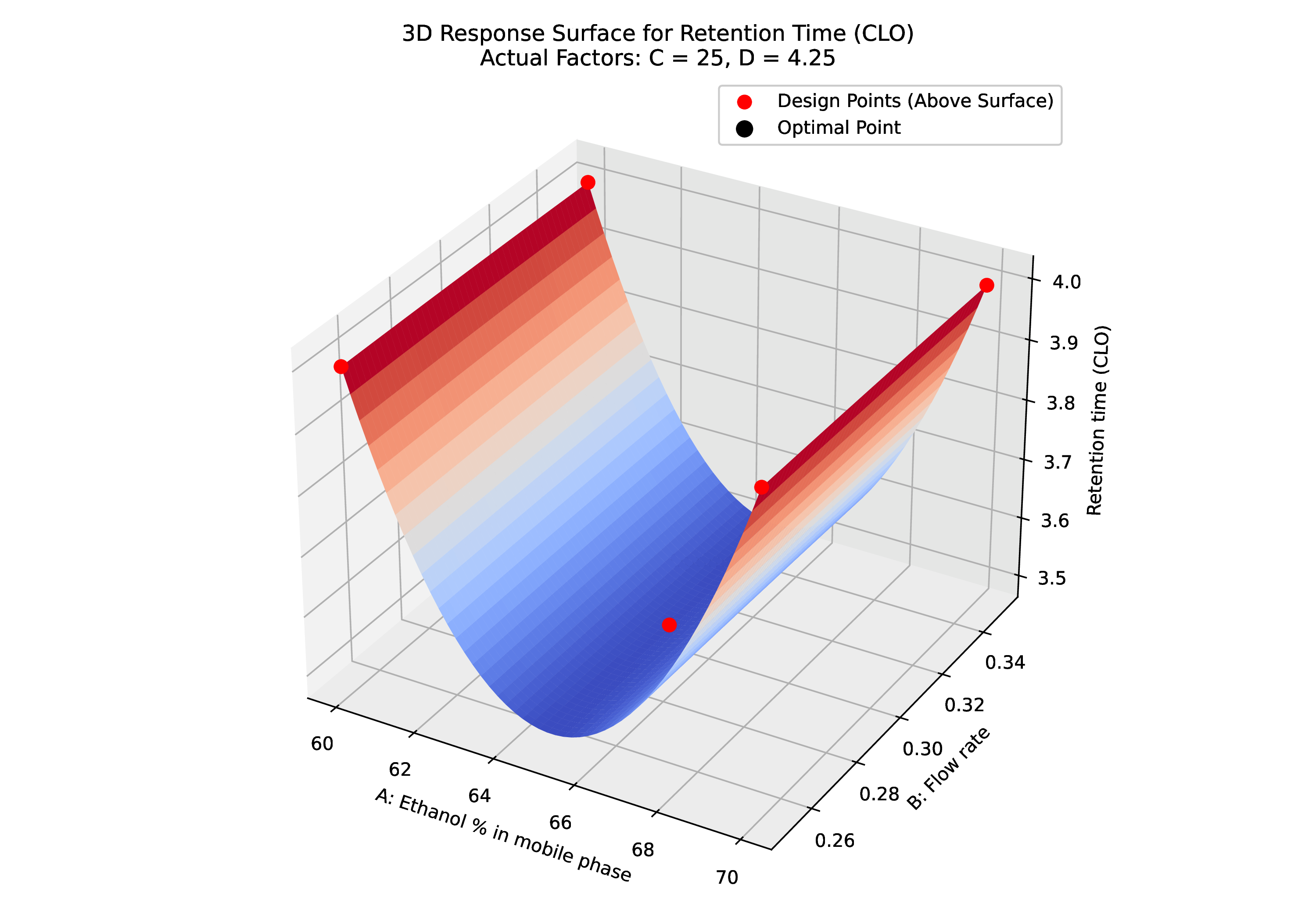


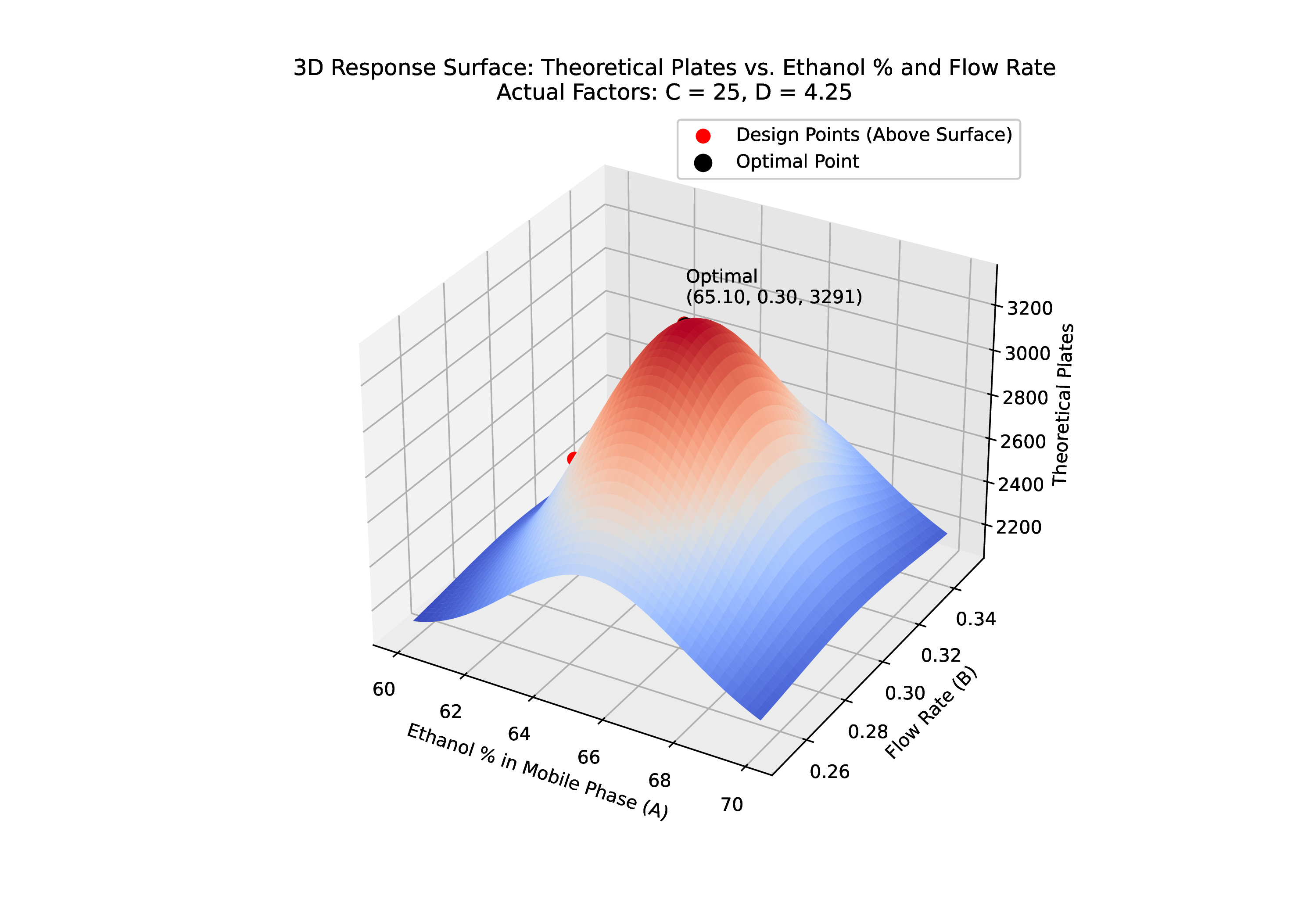


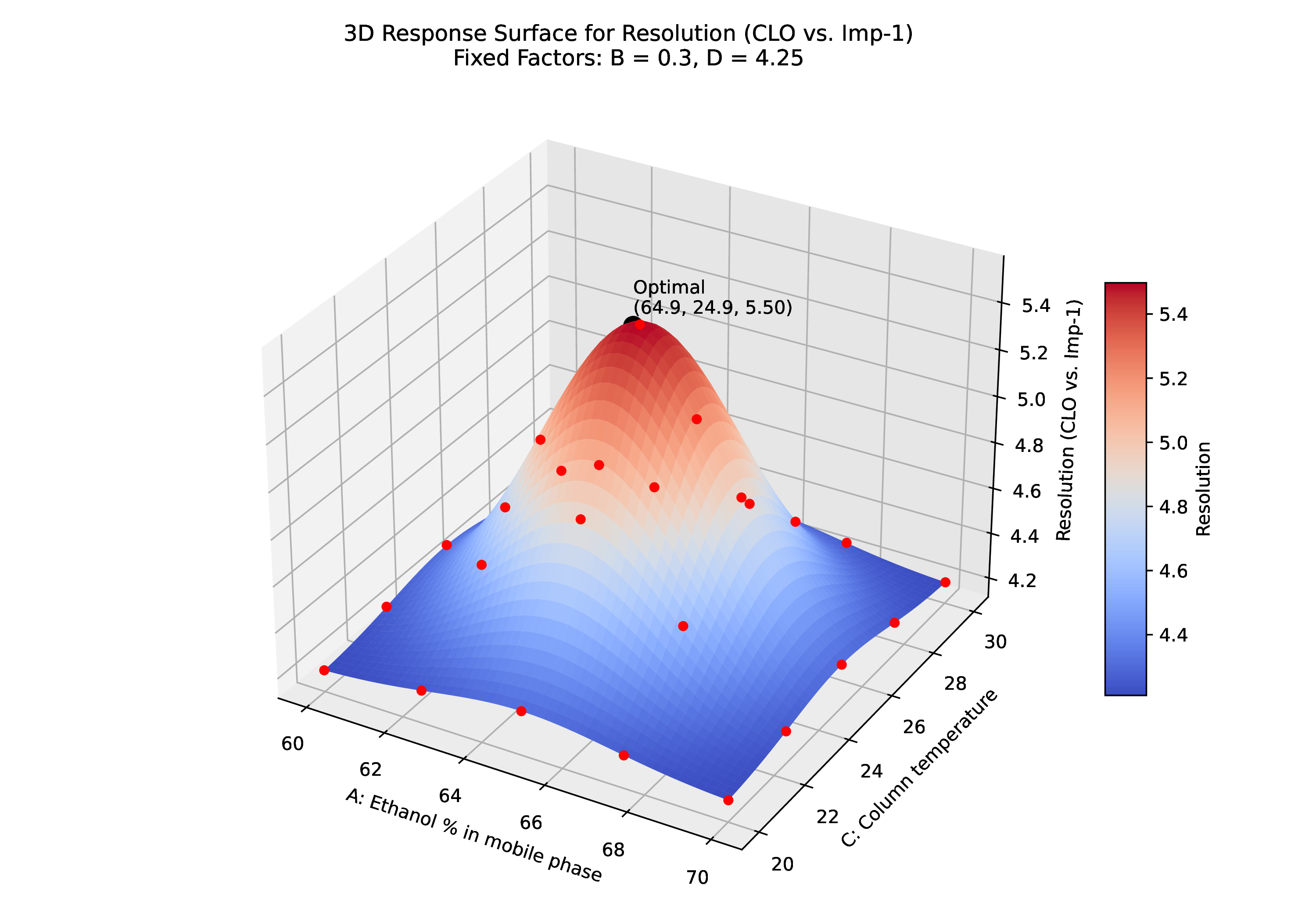


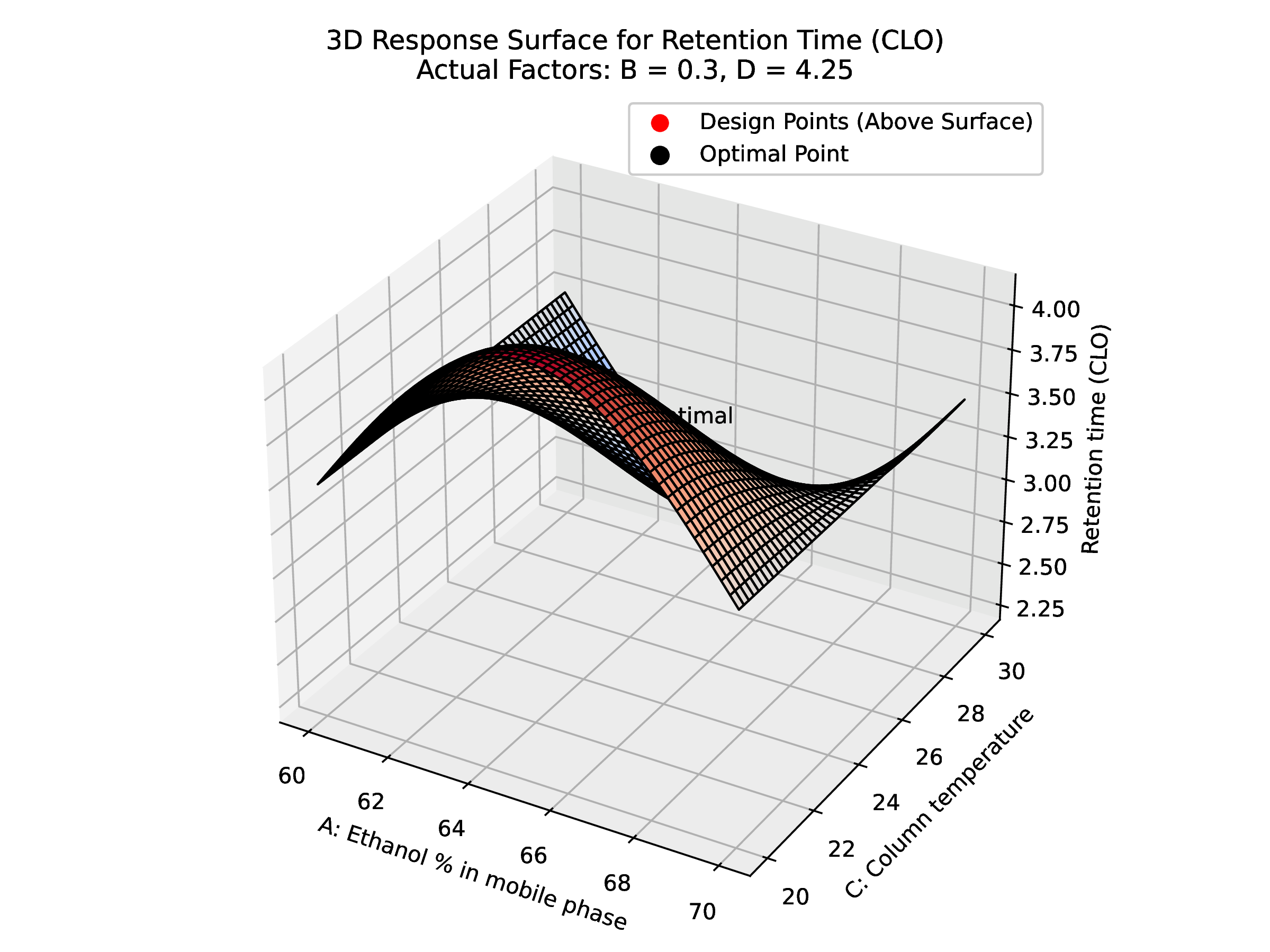


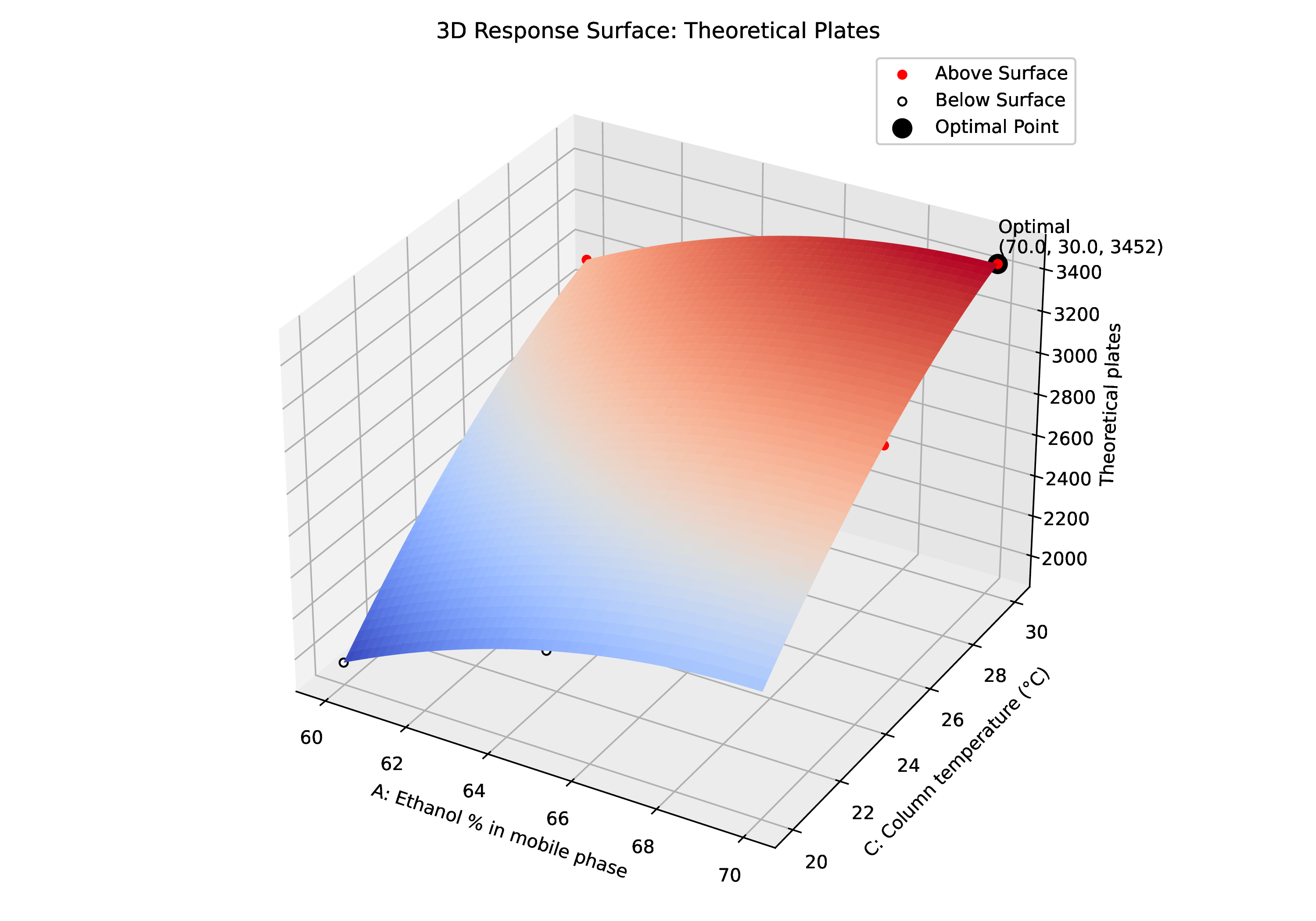


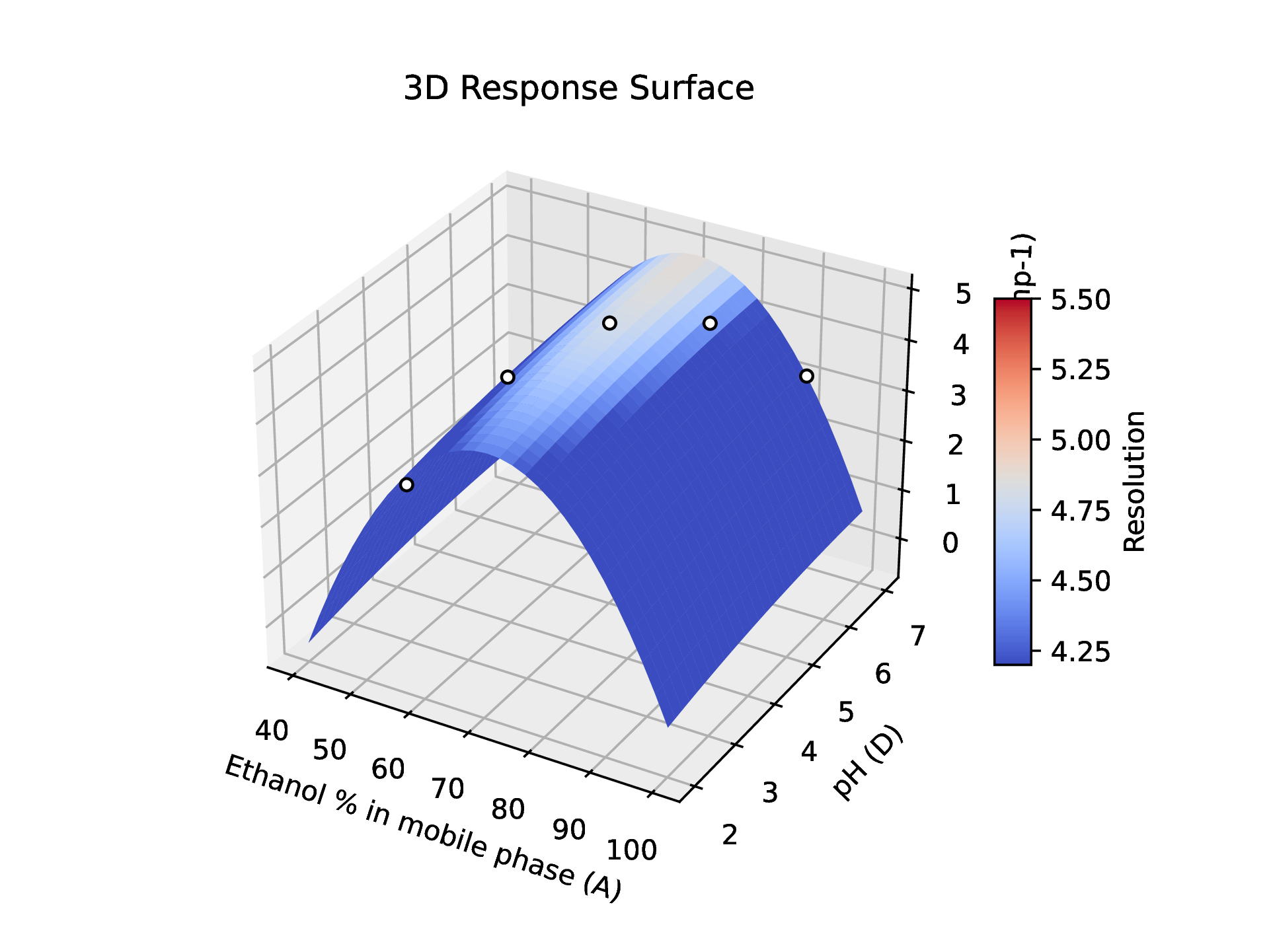


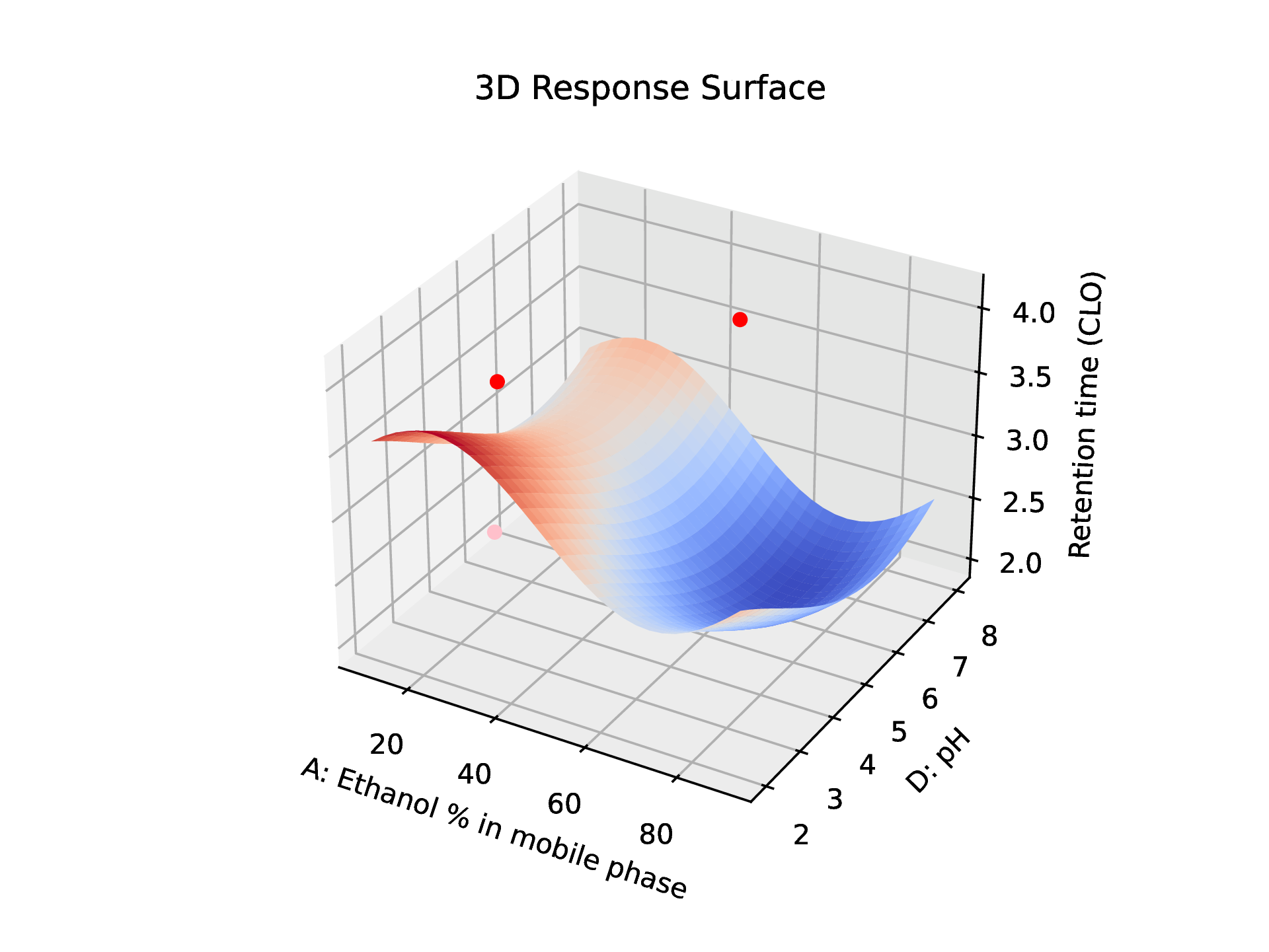

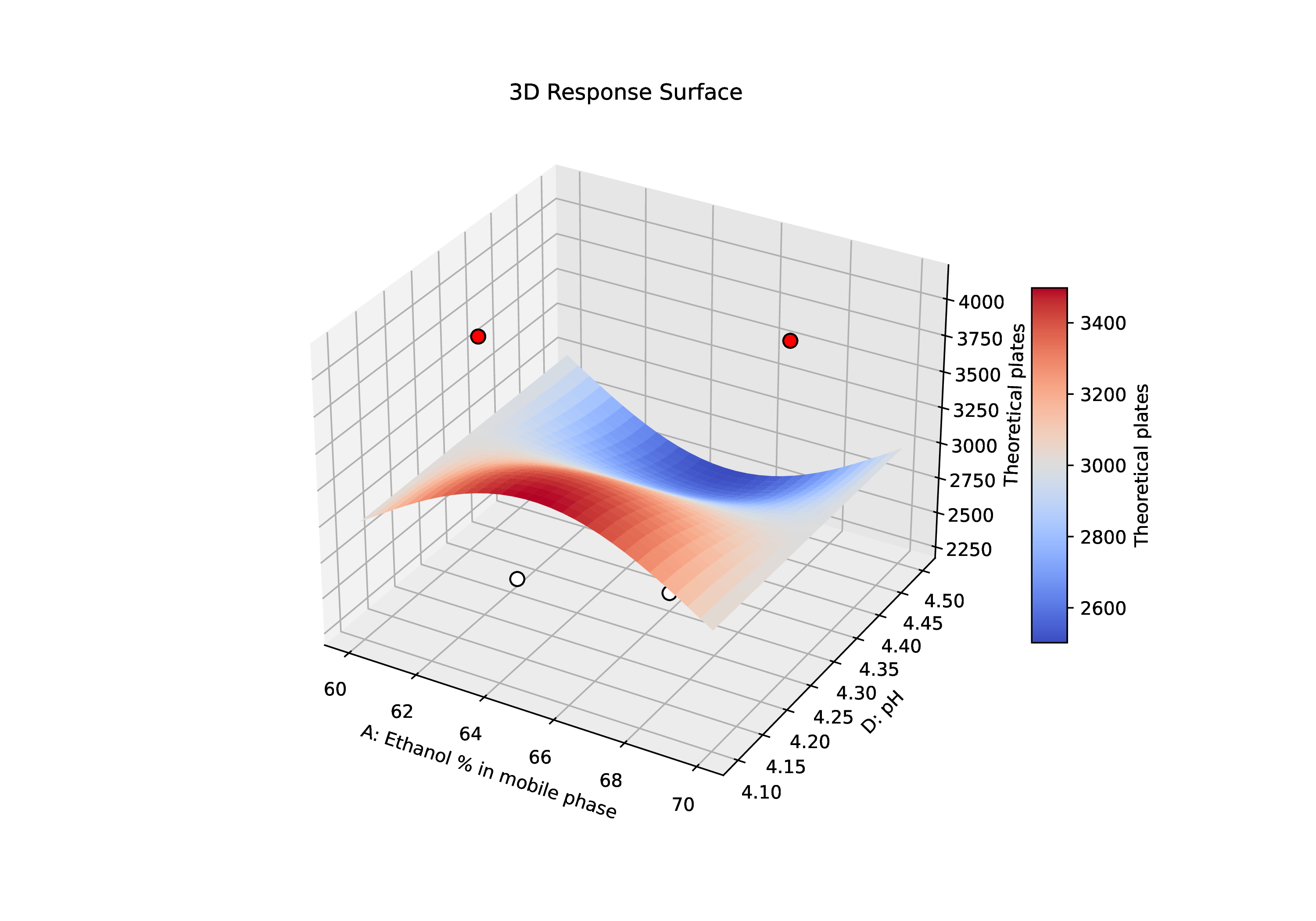

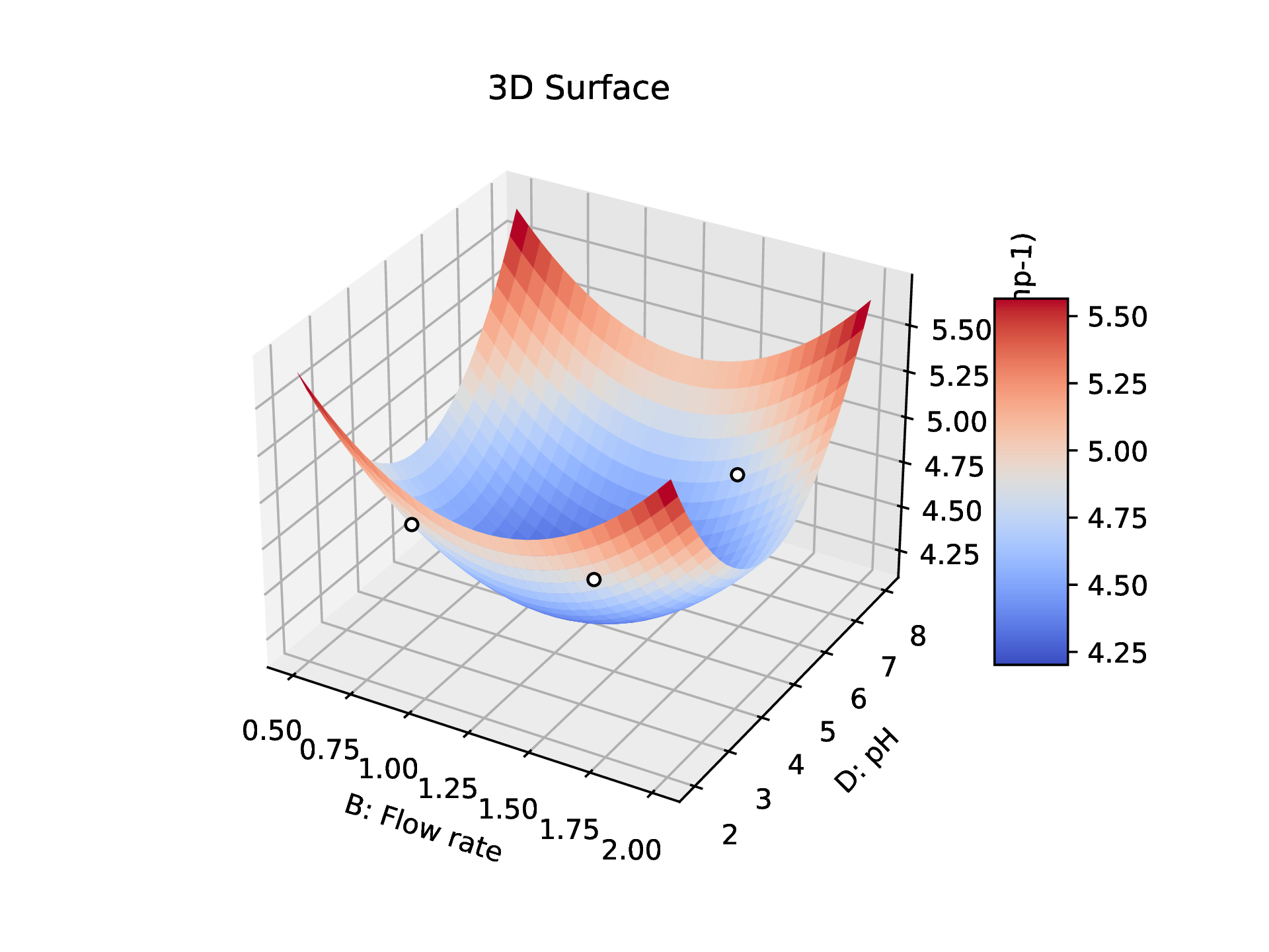

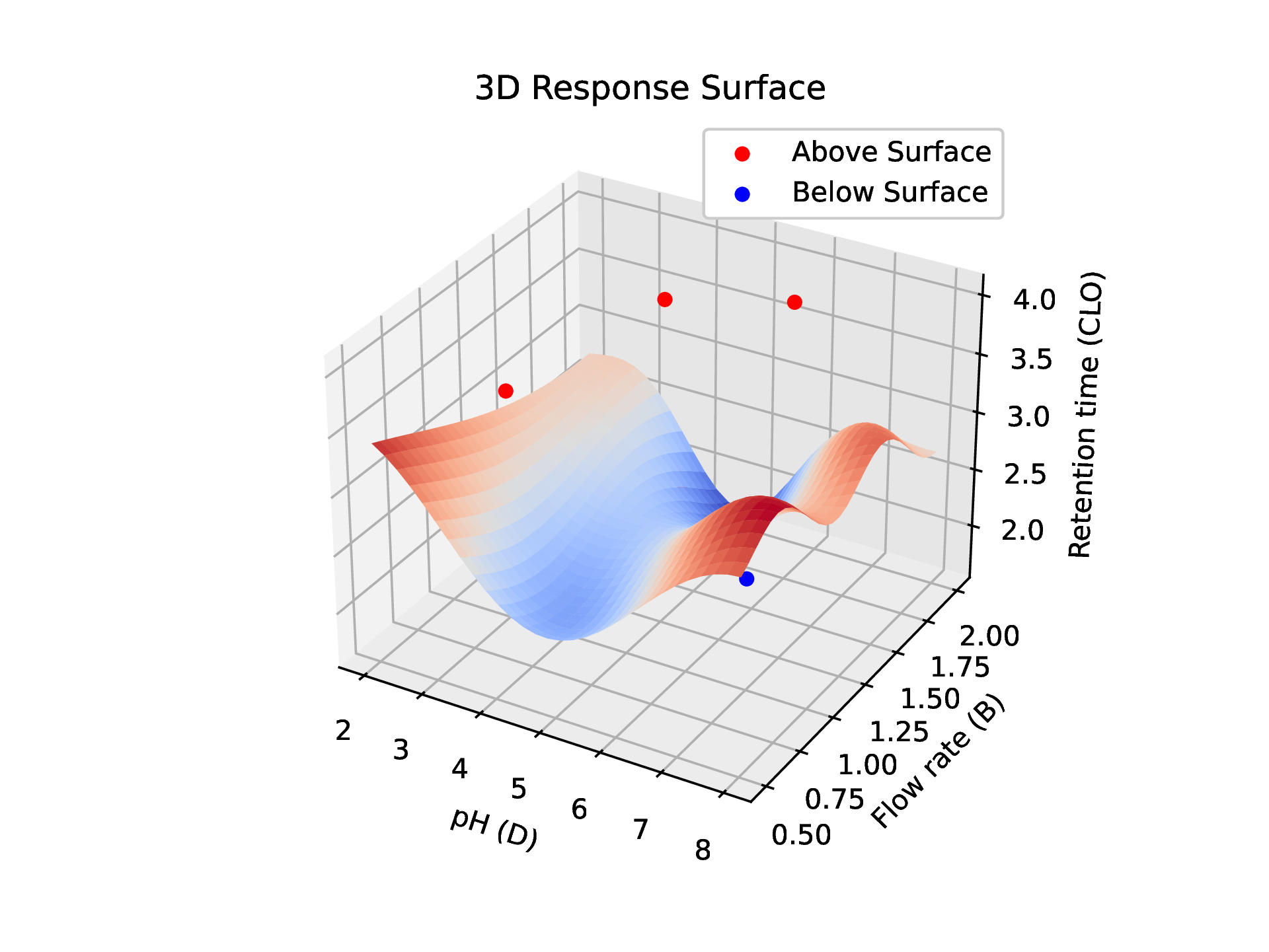

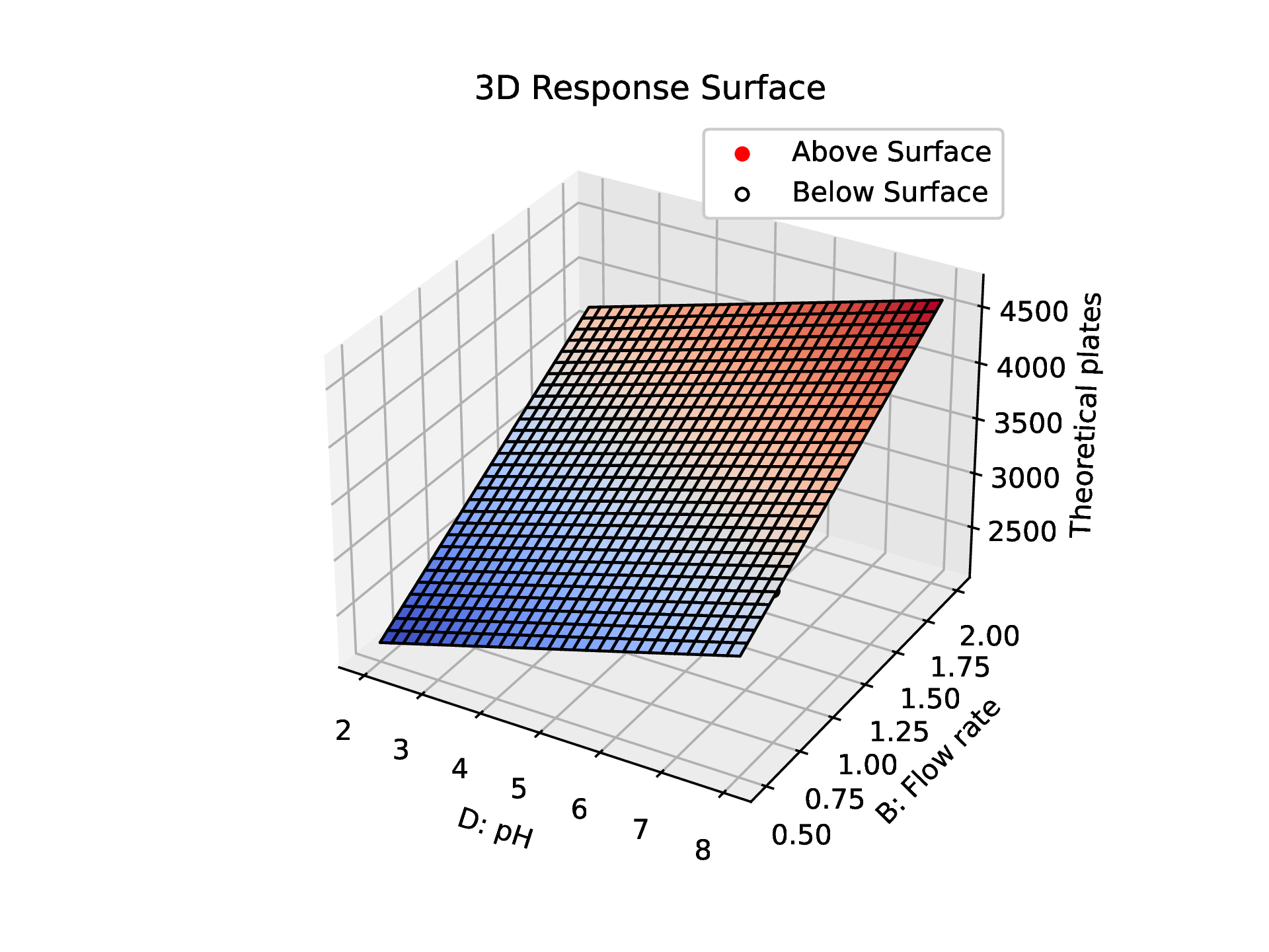

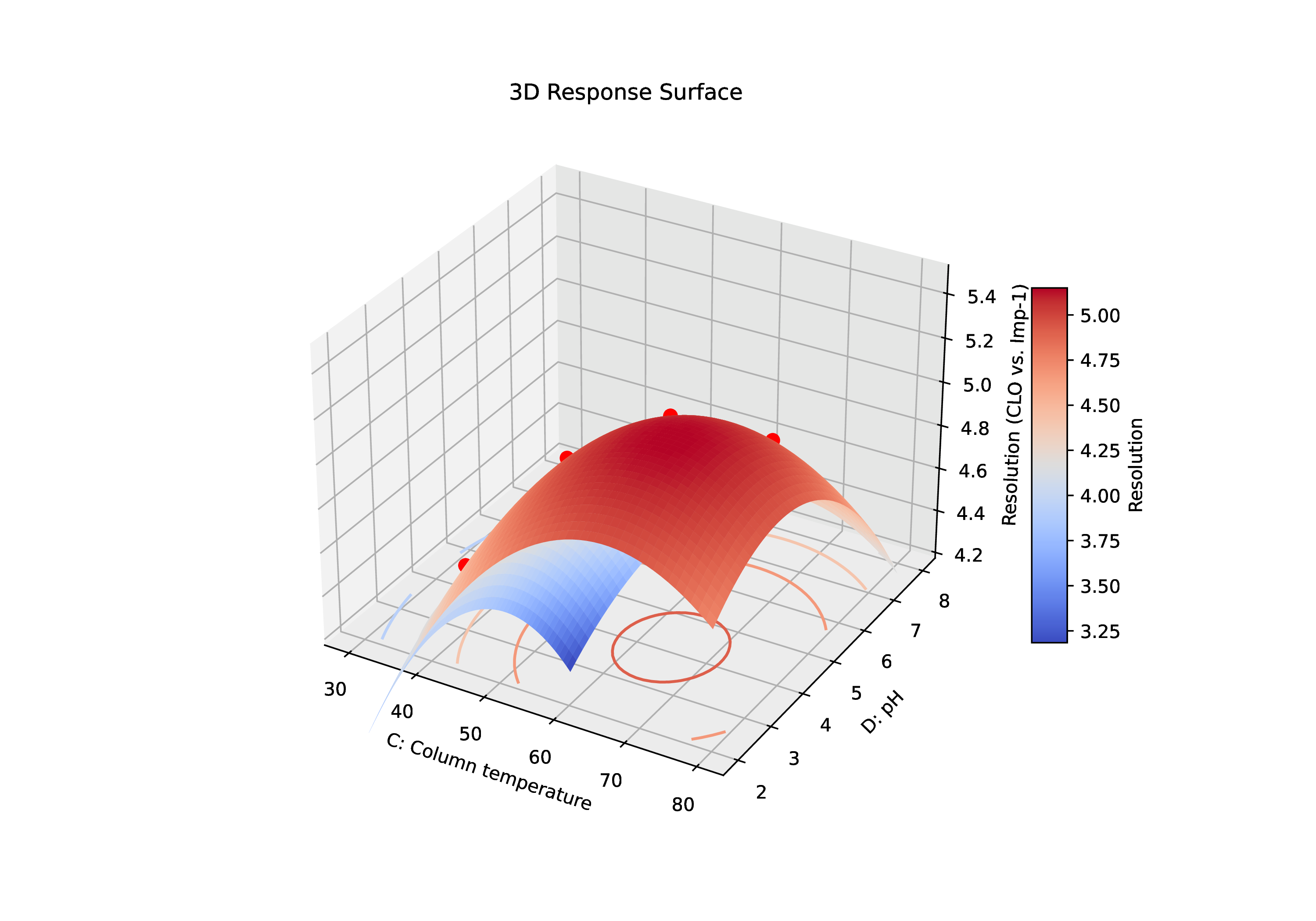

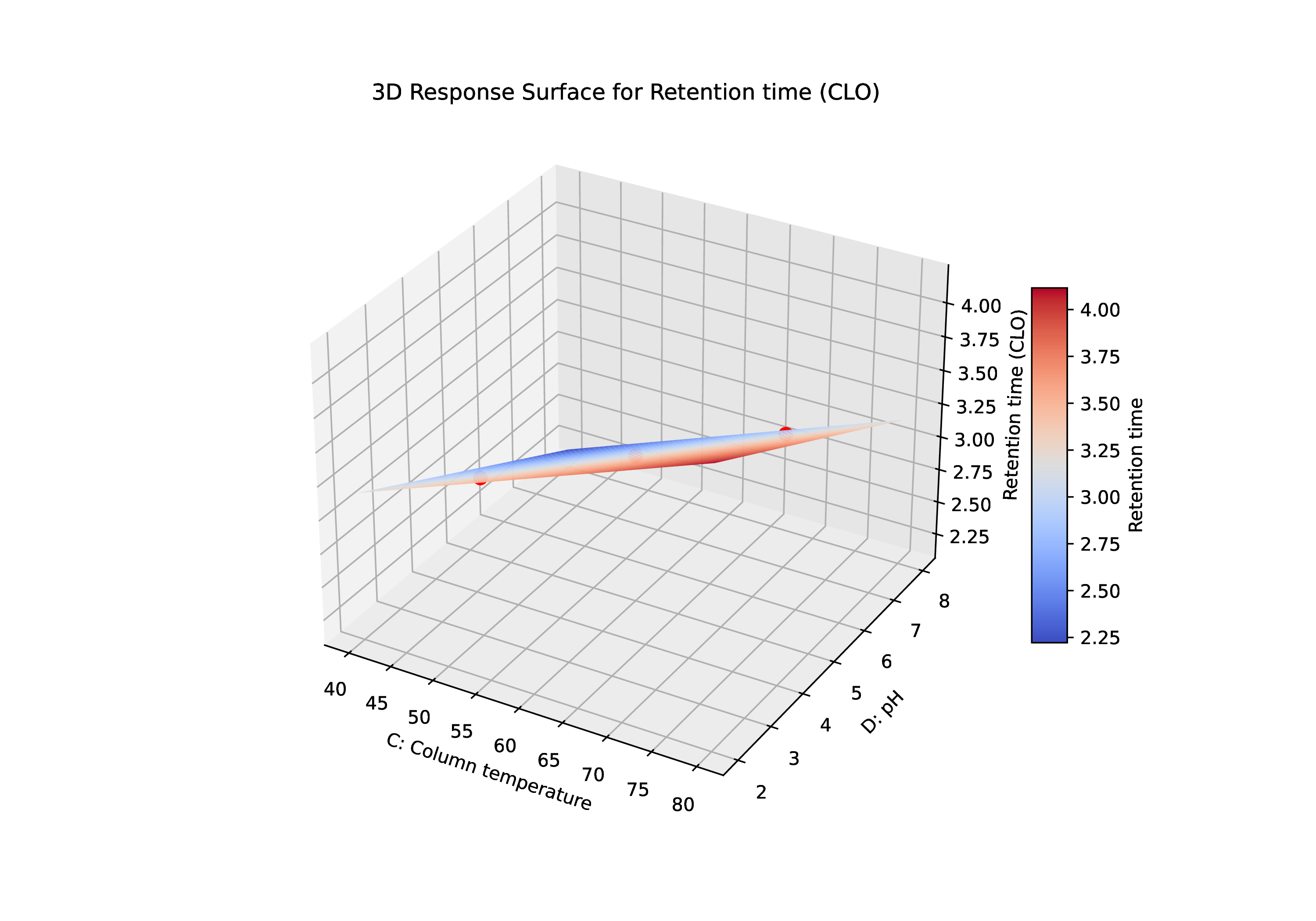

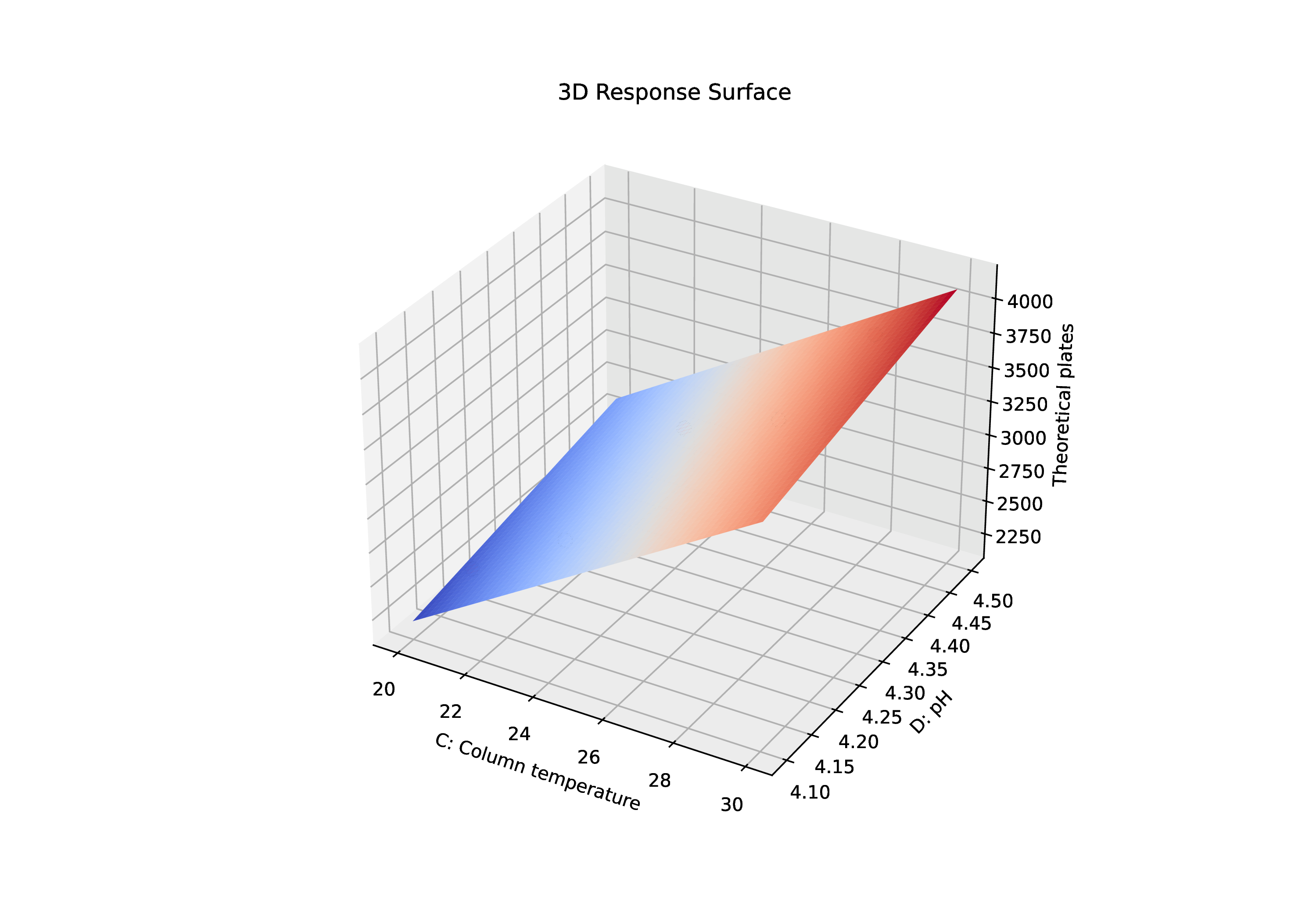

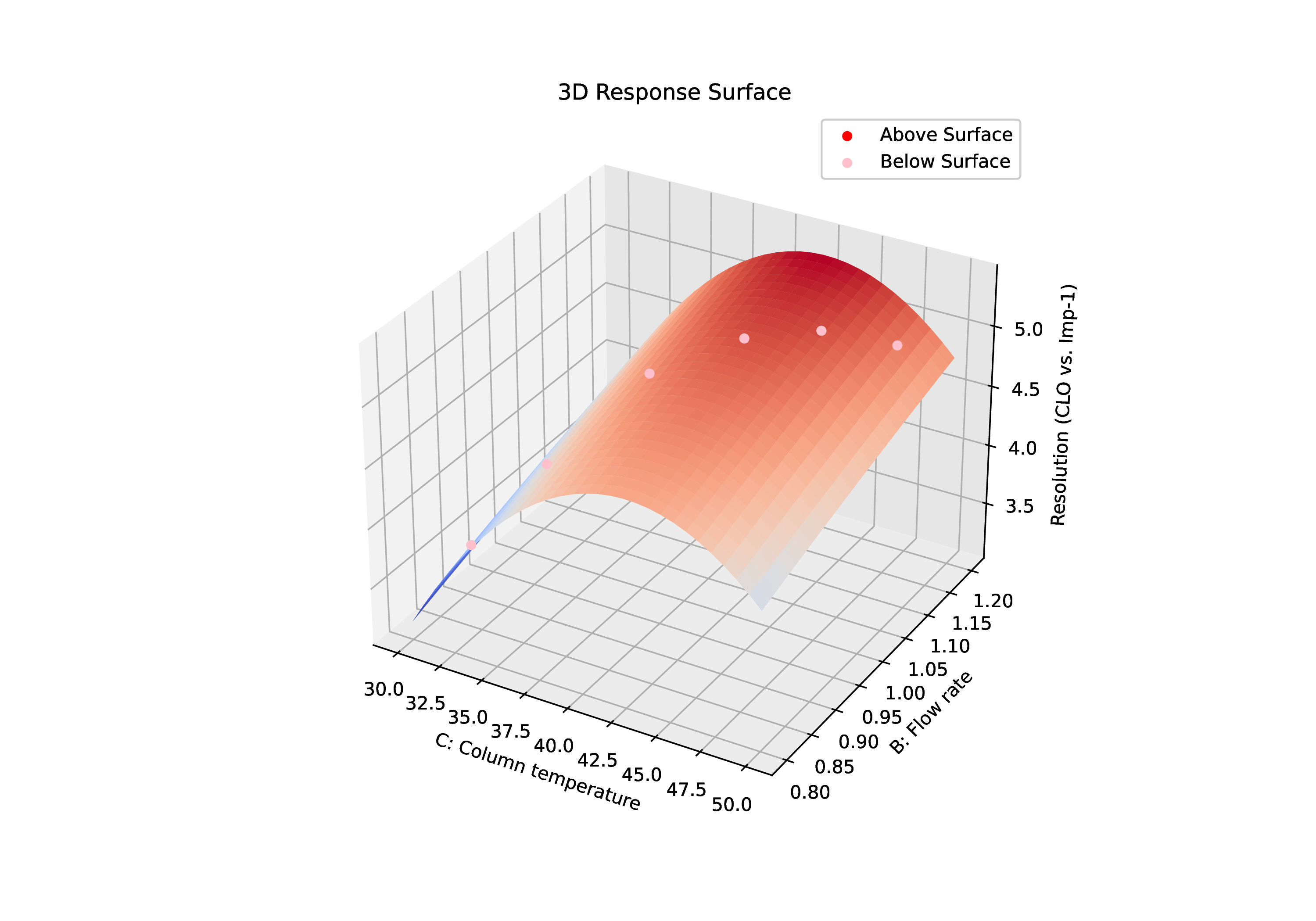

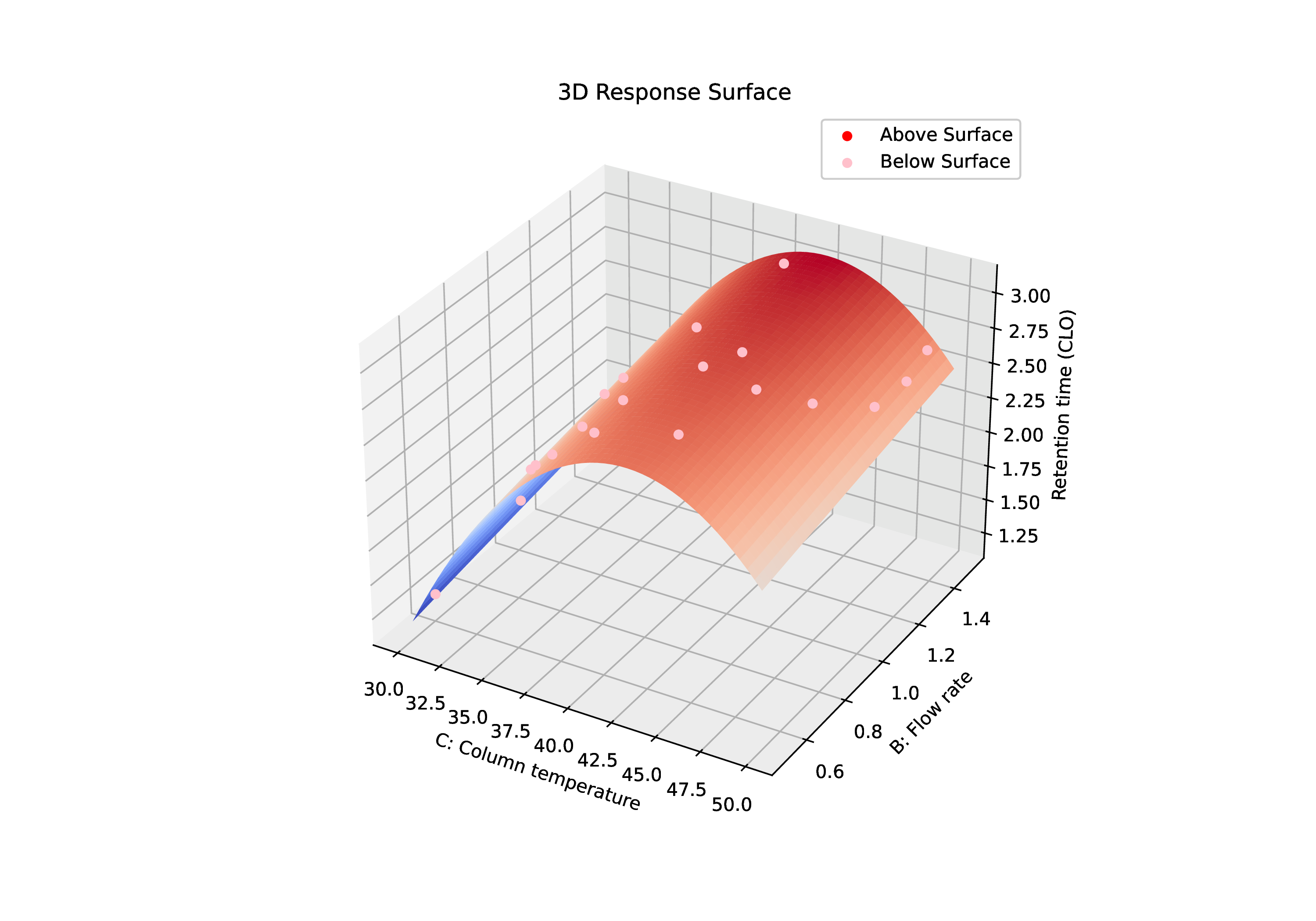

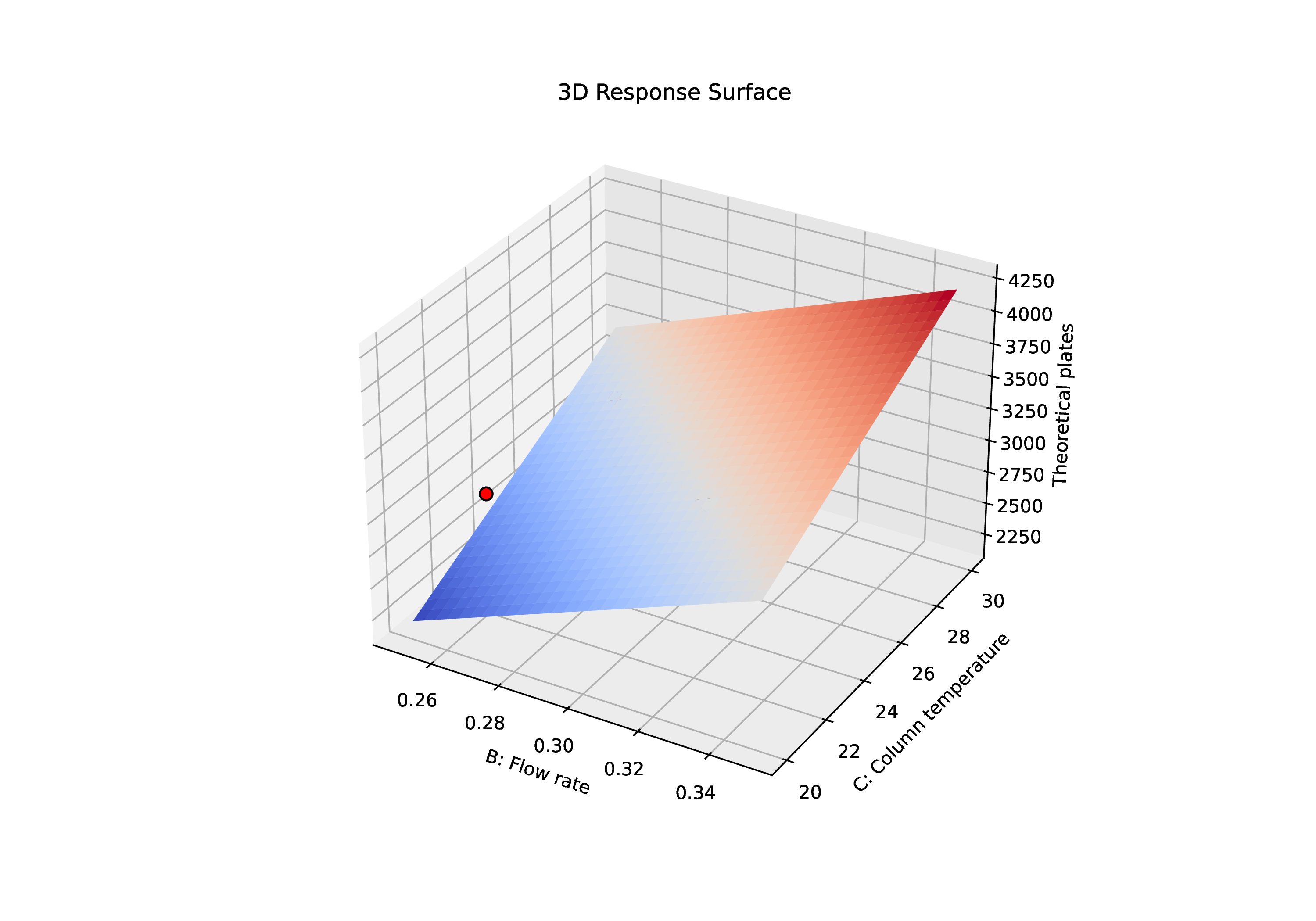


**Figure S3.** A 3D plot with response surfaces annotated with optimal points.


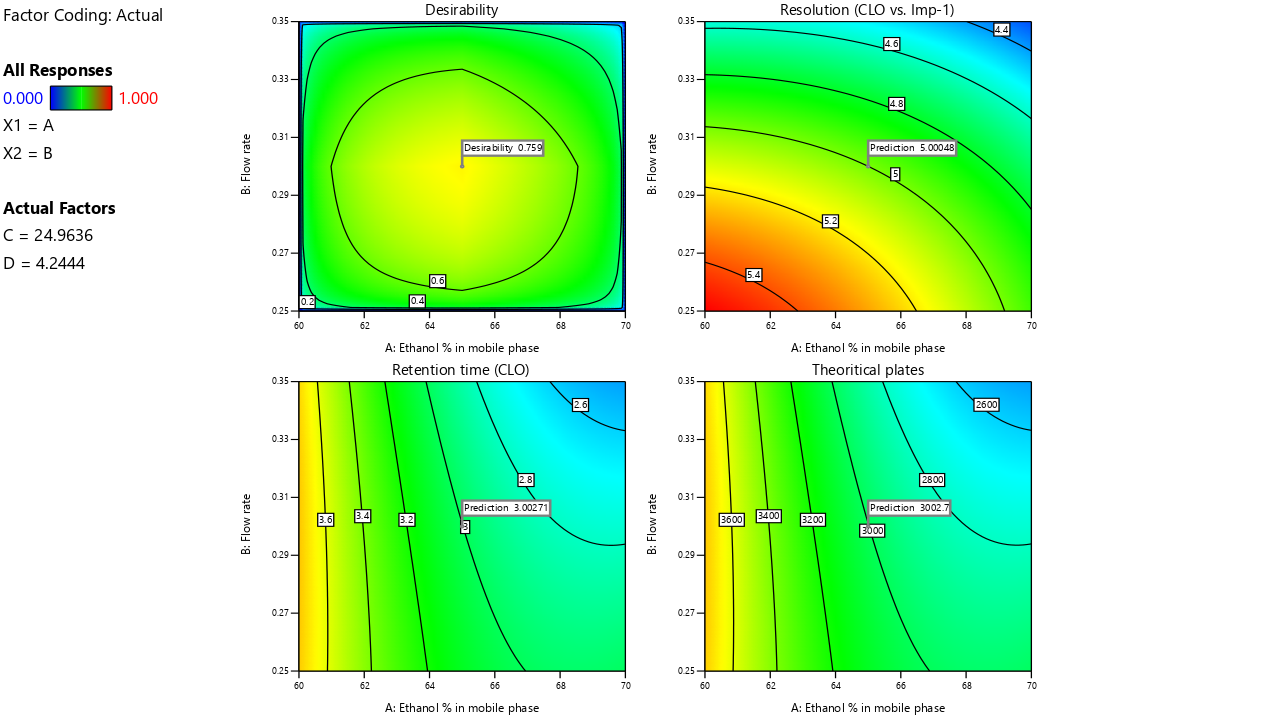


(a)


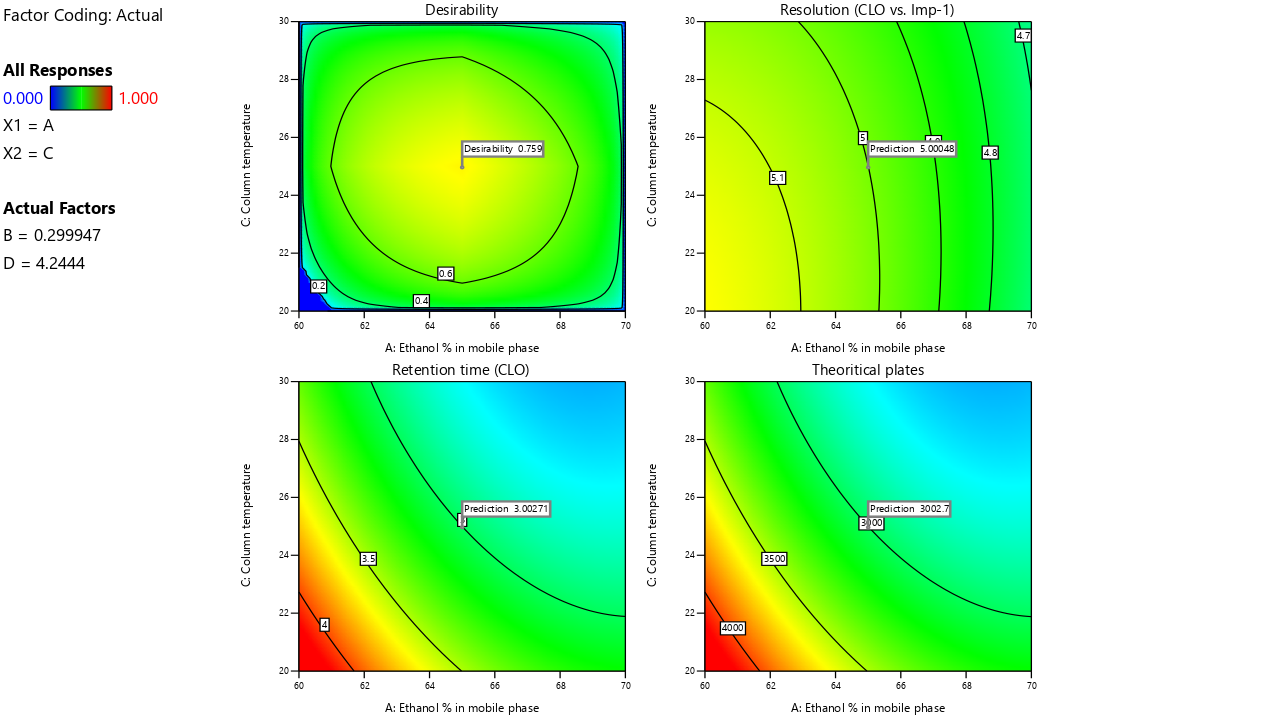


(b)


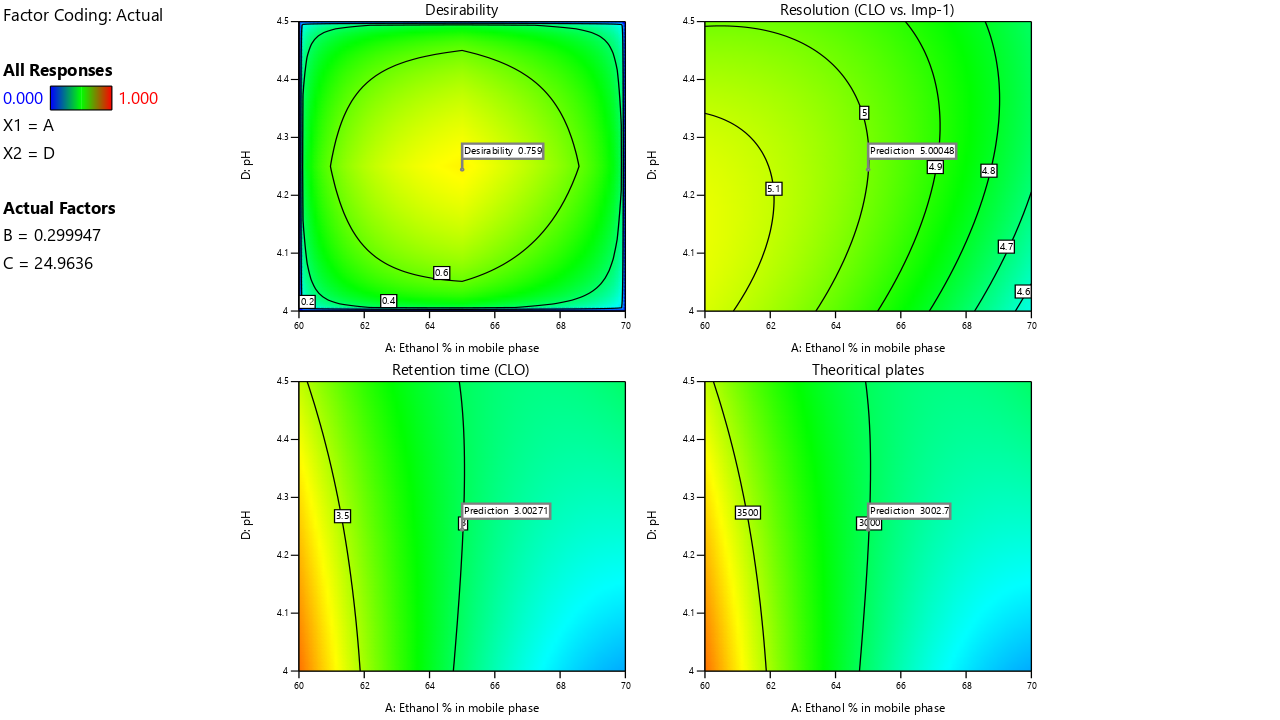


(c)


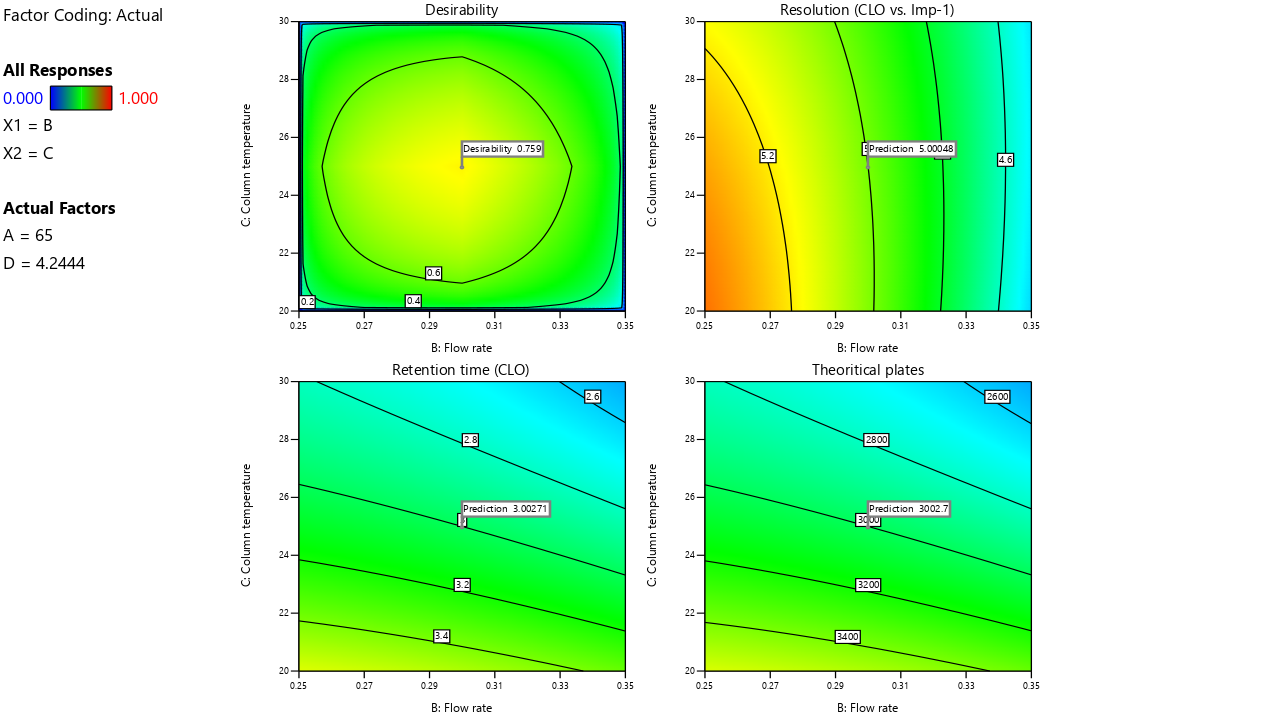


(d)


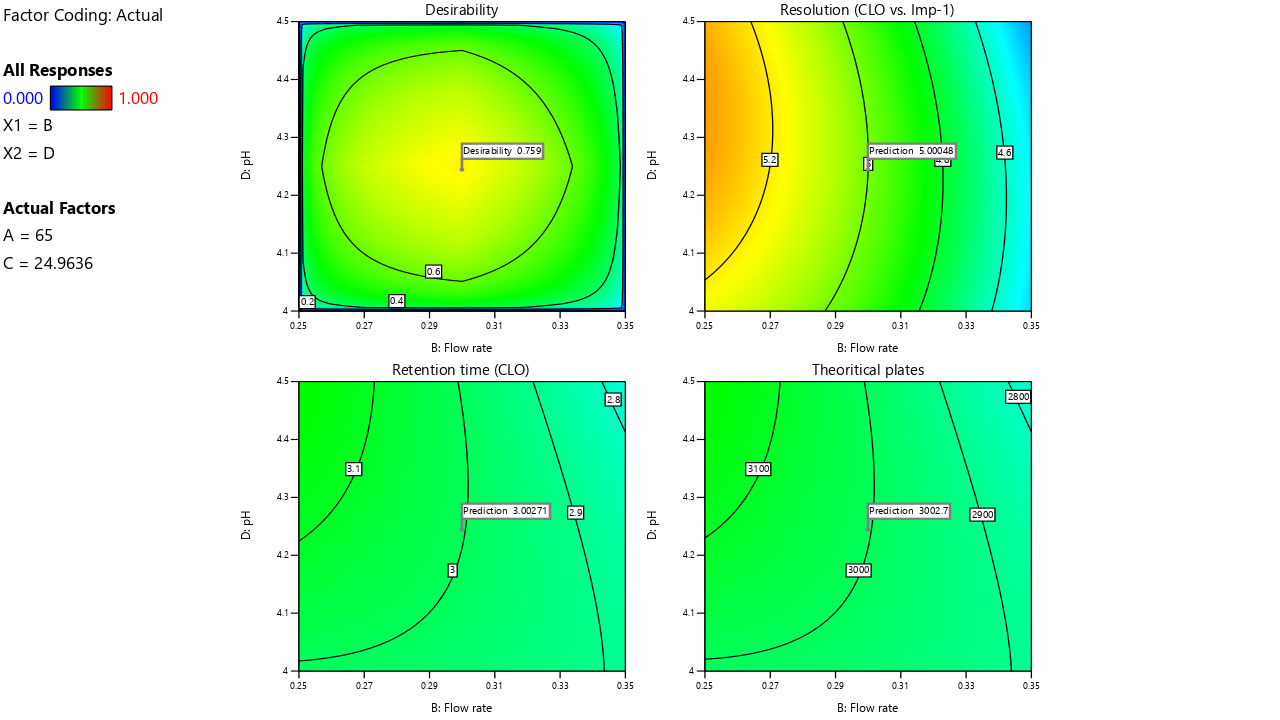


(e)


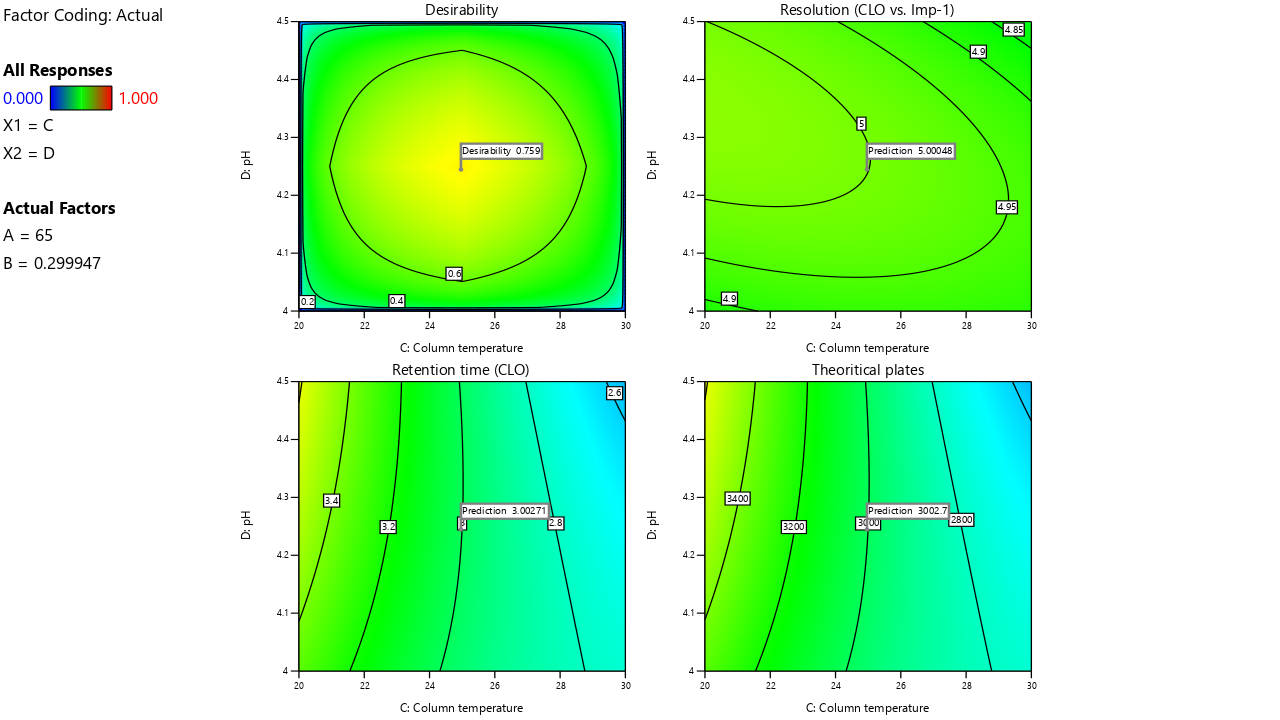


(f)

**Figure S4.** Predicting Optimal Method Conditions Using Desirability Functions (a-f).


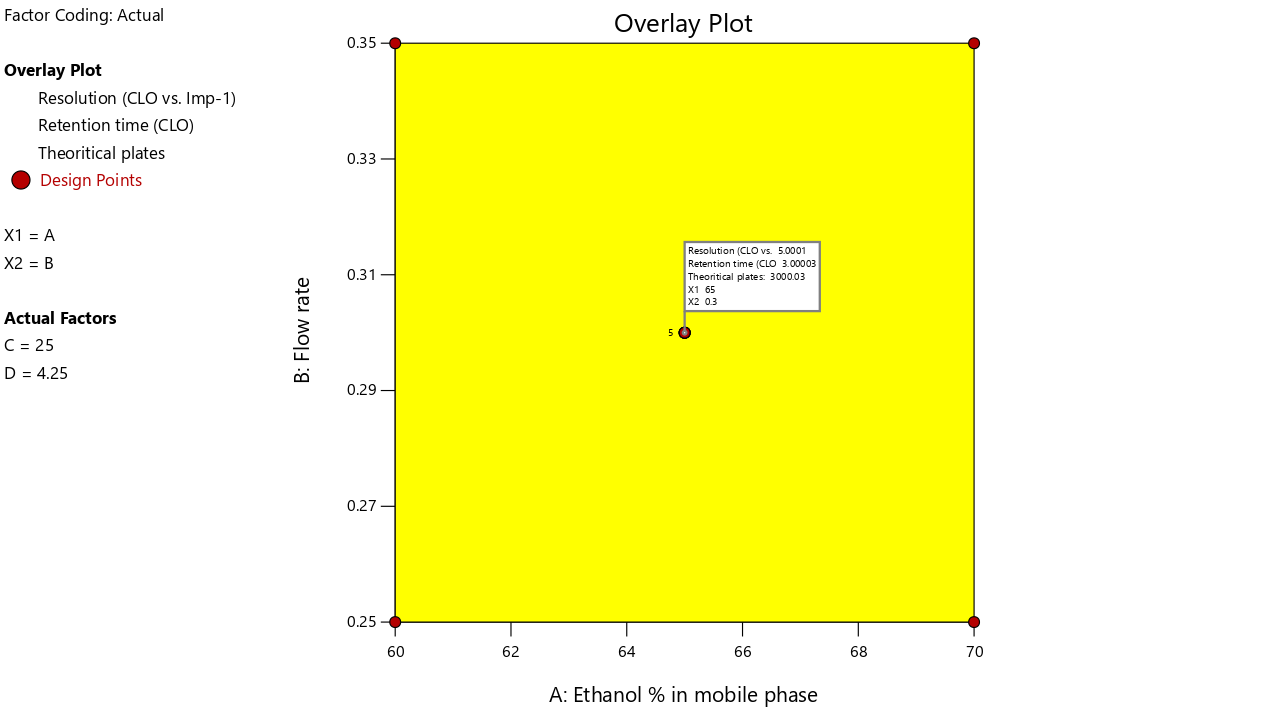


(a)


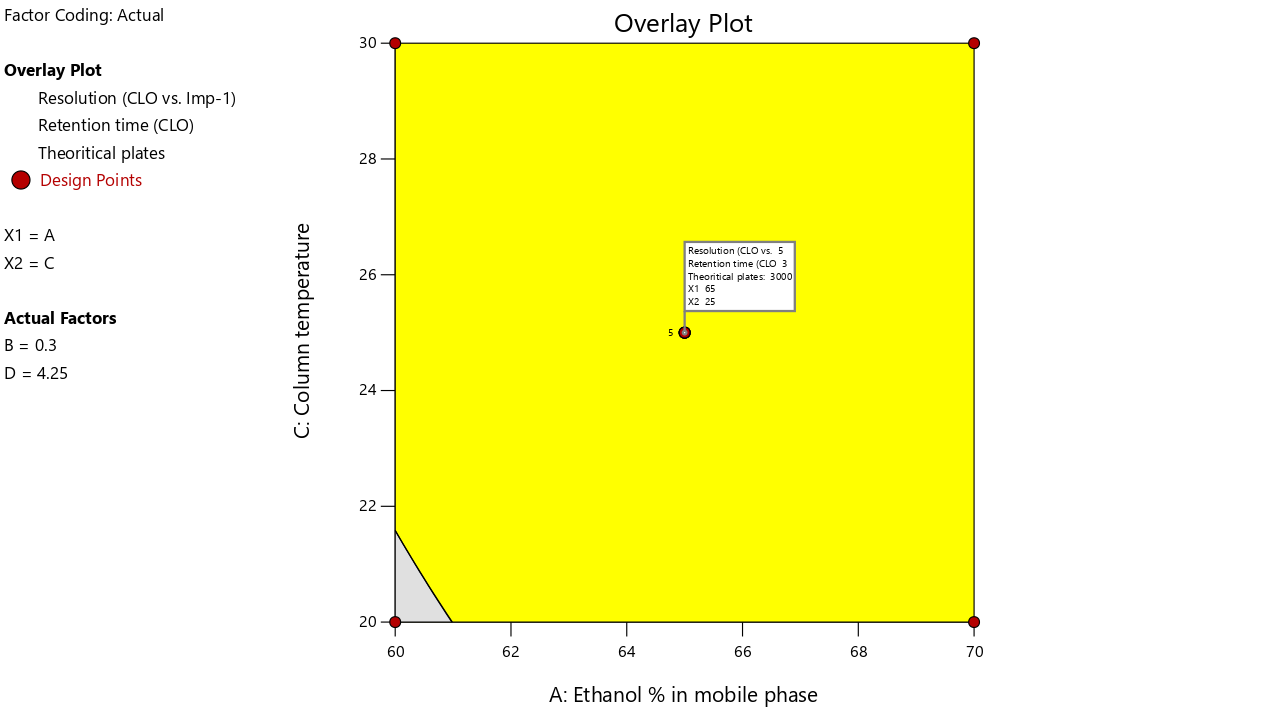


(b)


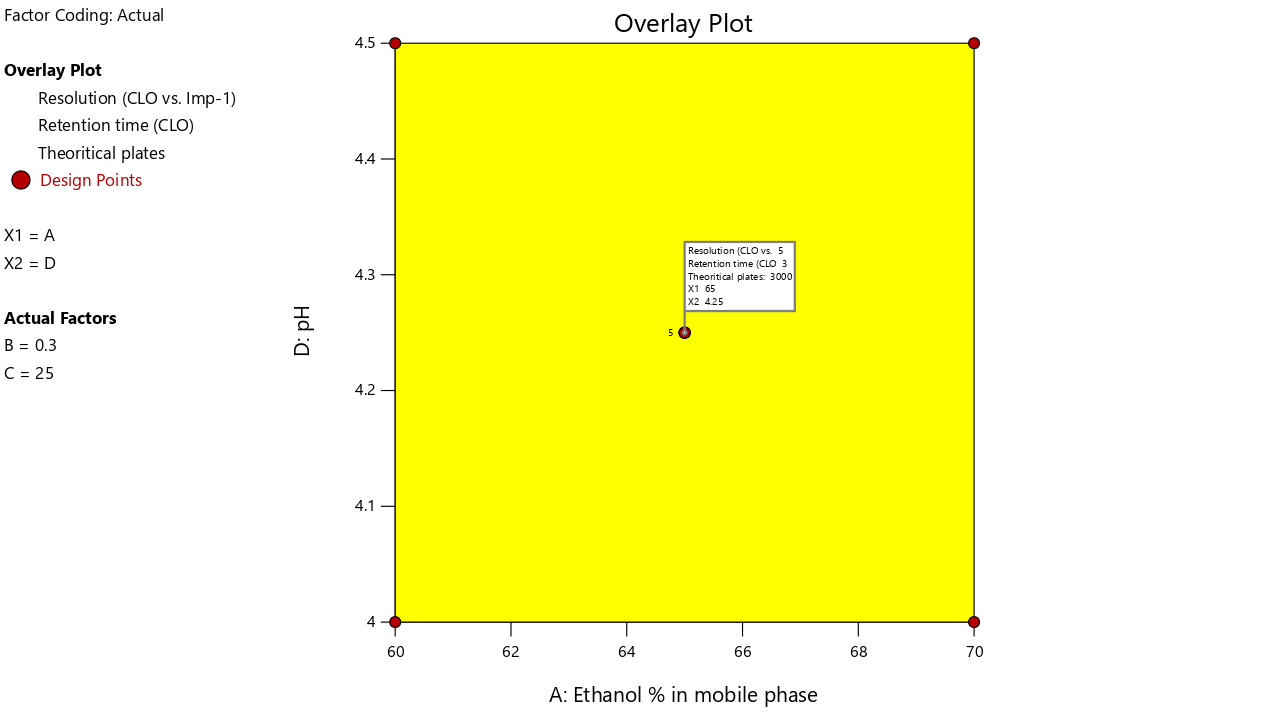


(c)


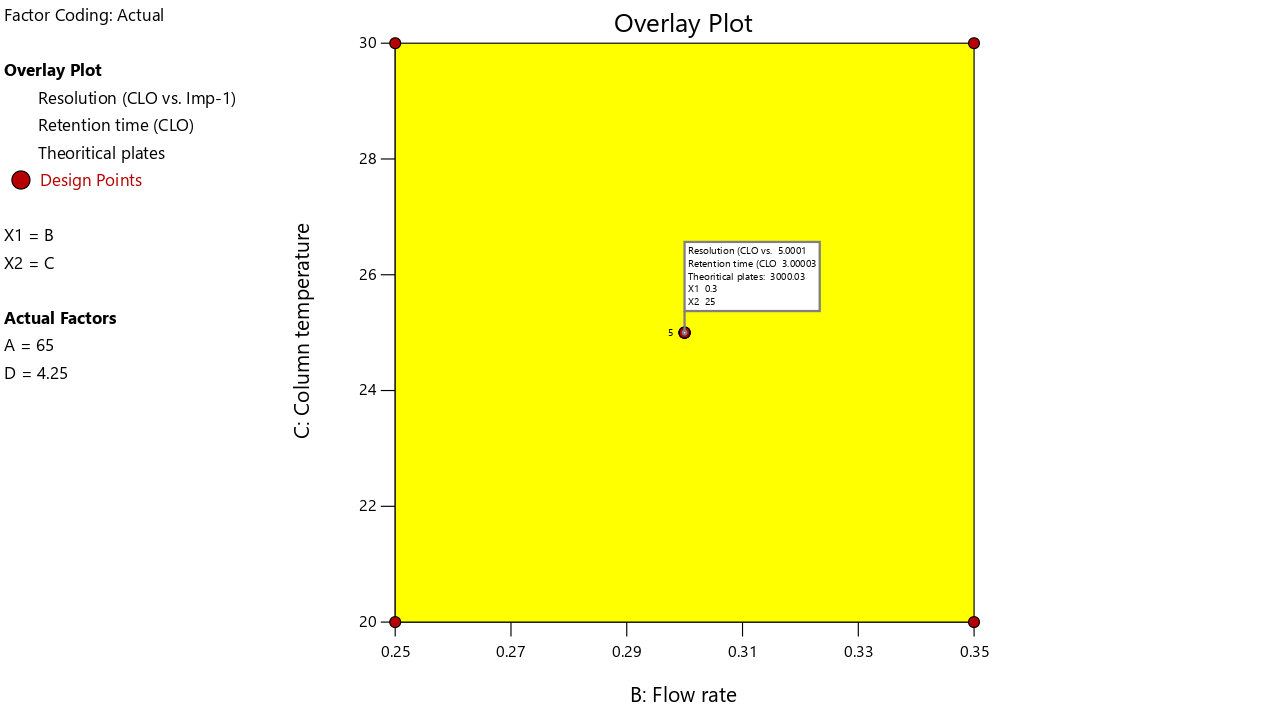


(d)


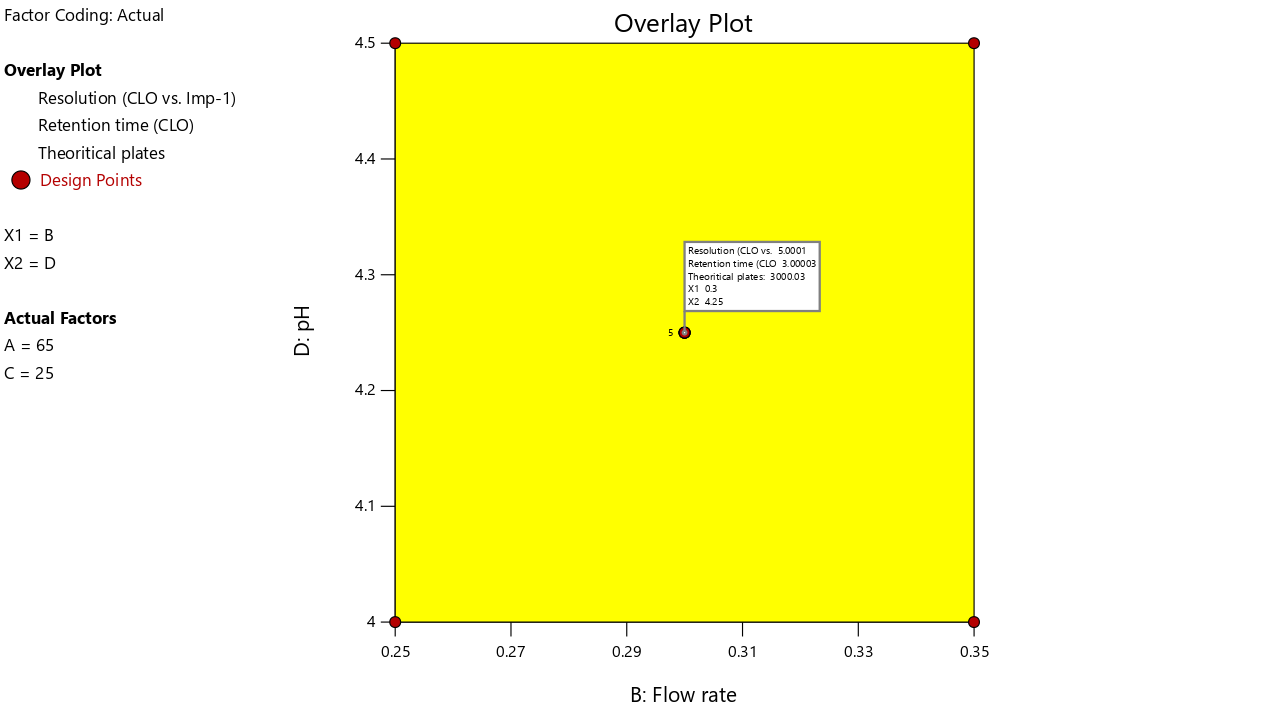


(e)


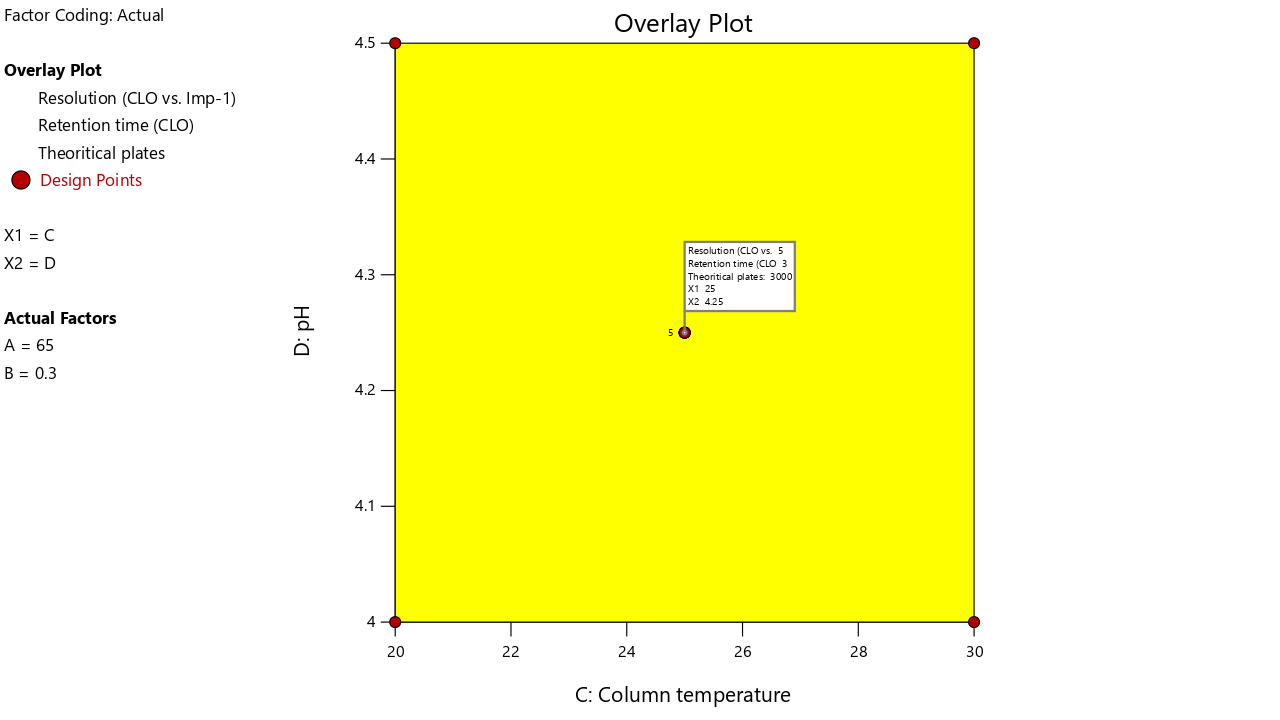


(f)


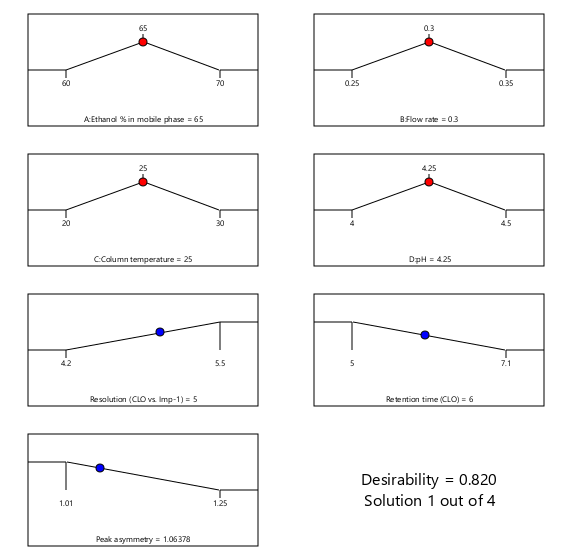


(g)

**Figure S5.** Integrating Overlay Plots and Desirability Functions for Method Performance Optimization (a-g).


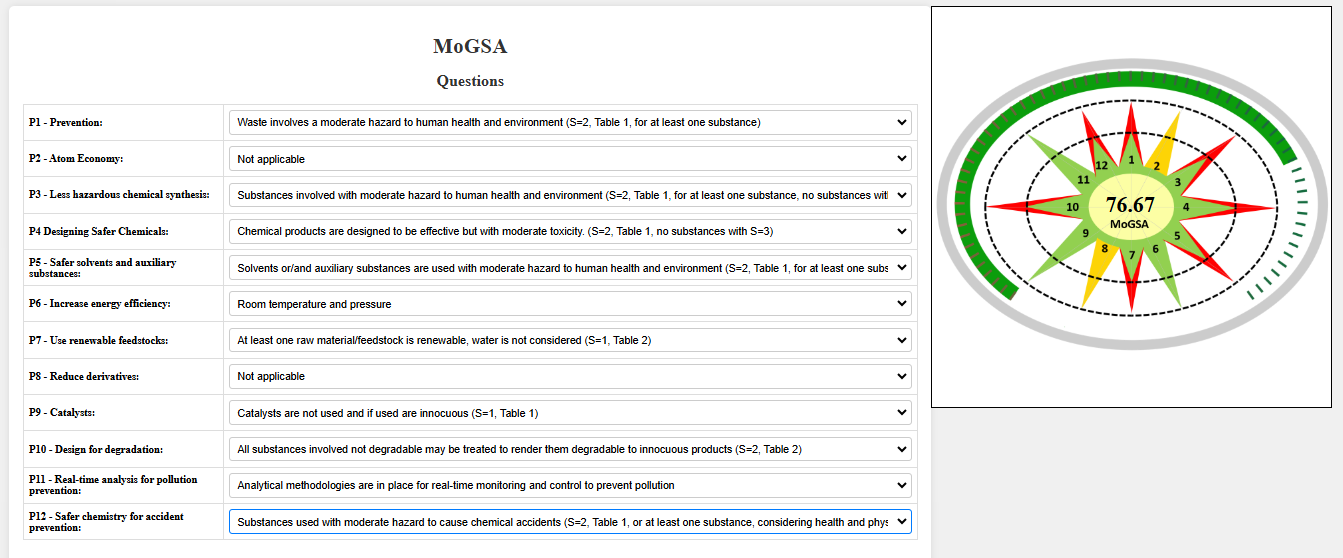


(a)


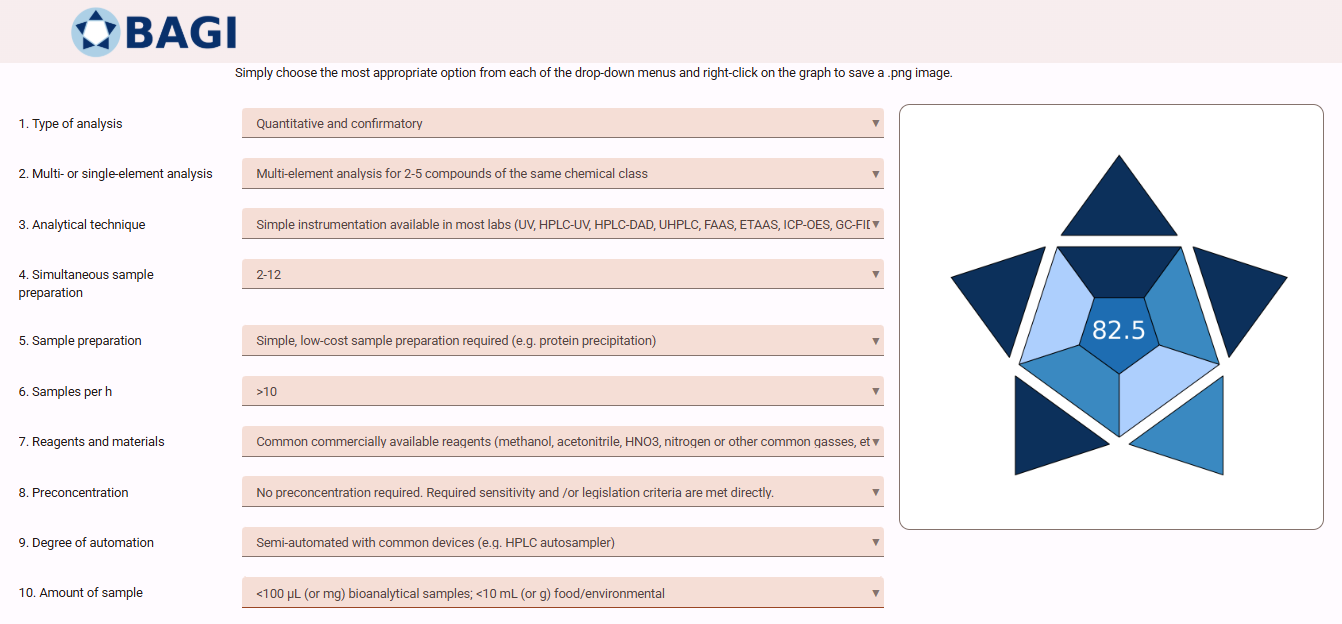


(b)


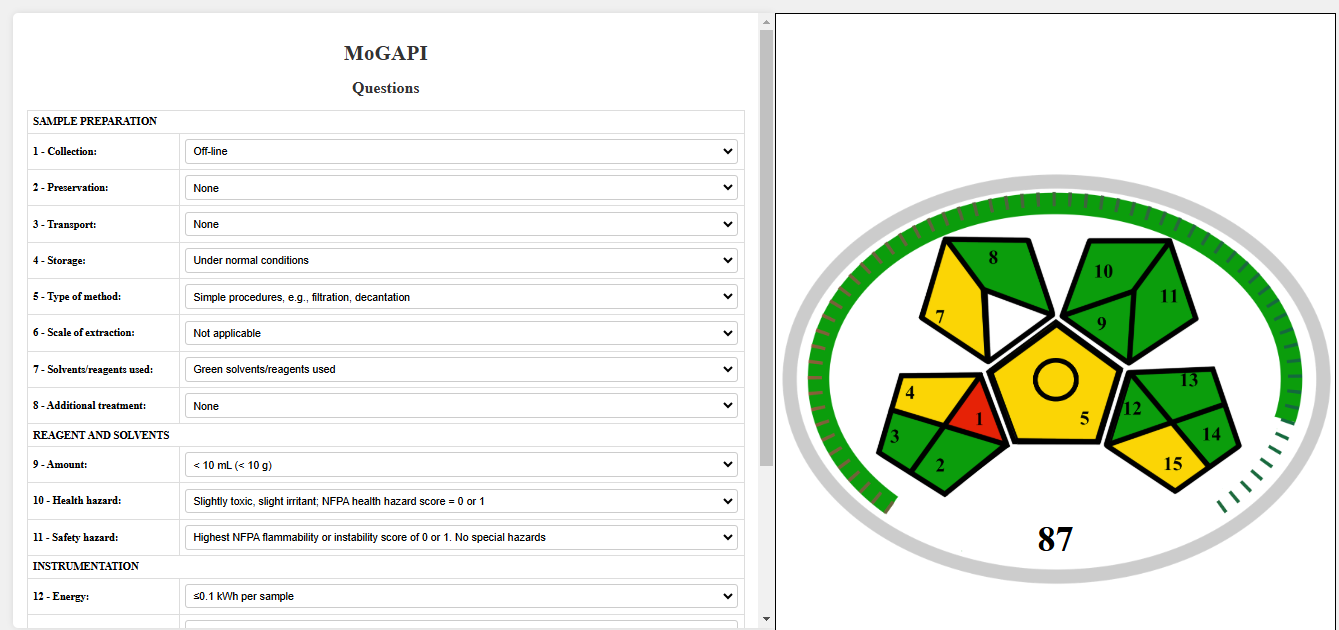


(c)

**Figure S6.** Appraisal tools of (a) MoGSA, (b) BAGI, and (c) MoGAPI for the recommended UPLC method

**Table S1.** Analysis of CLO drugs using the proposed method in comparison with existing methods.

| **Method** | **Proposed Method** | **Reported Method**[18] | **Reported Method  [**19**]** | **Reported Method  [**20**]** |
| --- | --- | --- | --- | --- |
| MoGSA | 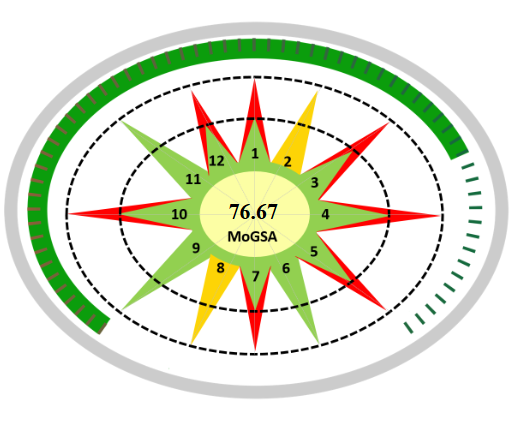 | 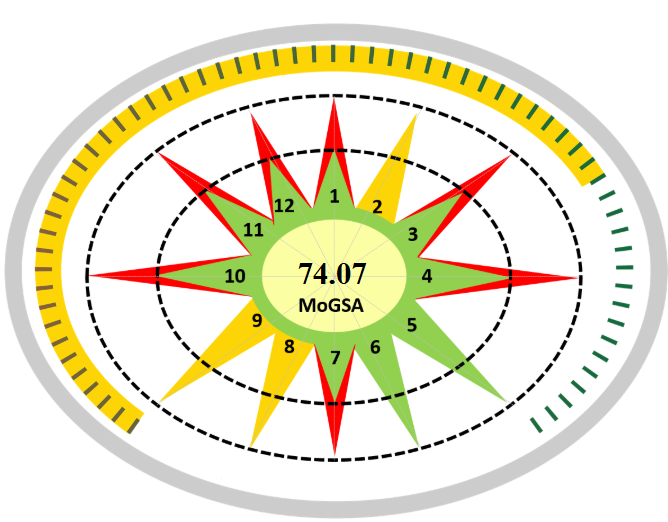 | 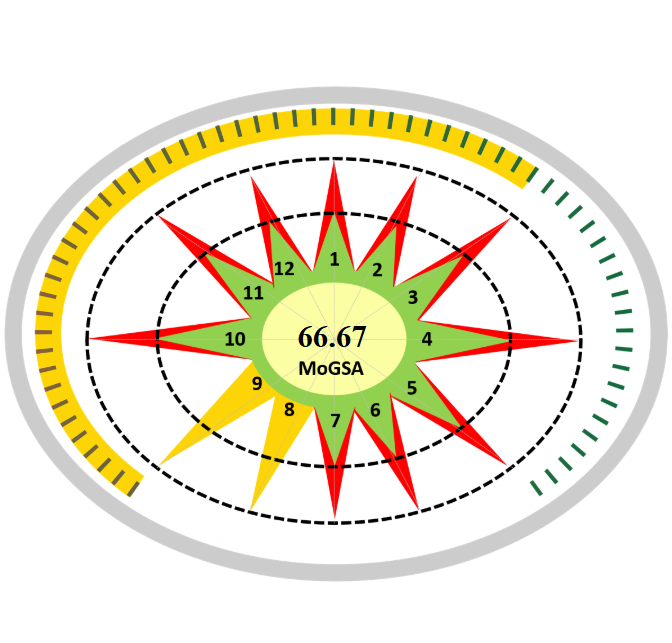 | 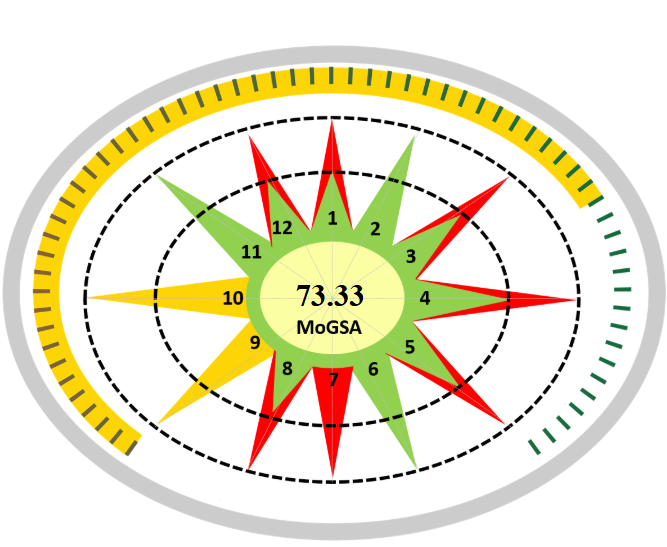 |
| BAGI | 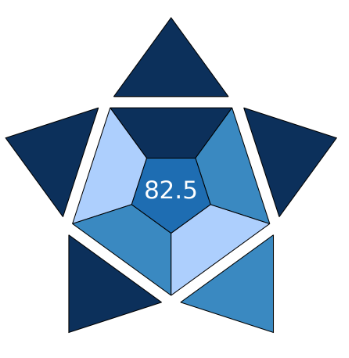 | 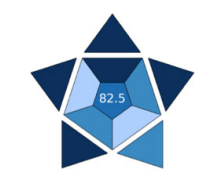 | 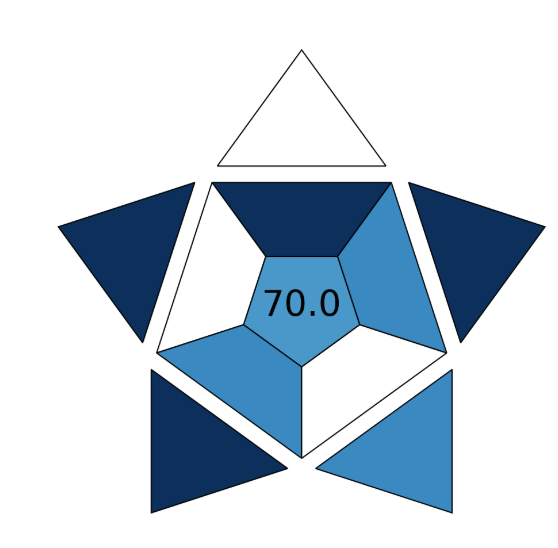 | 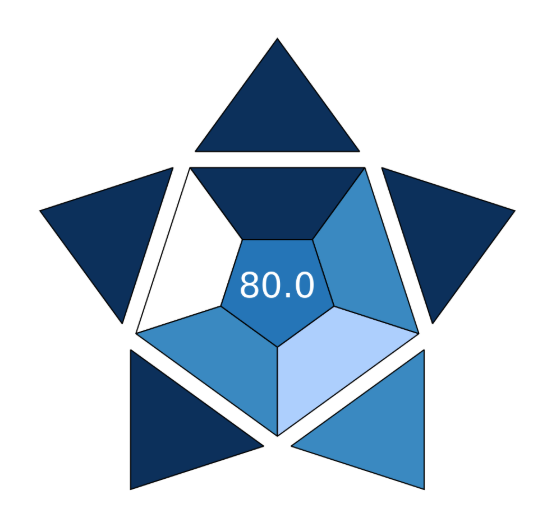 |
| MoGAPI | 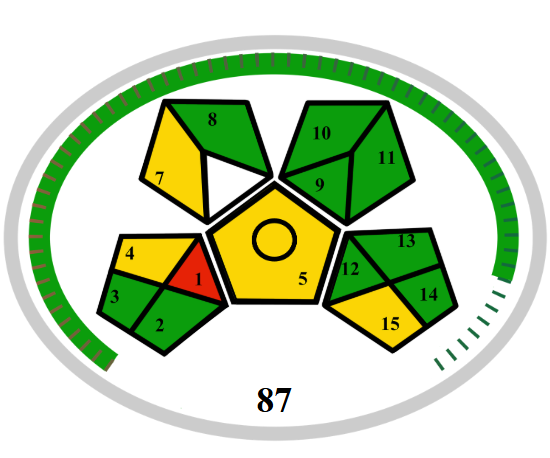 | 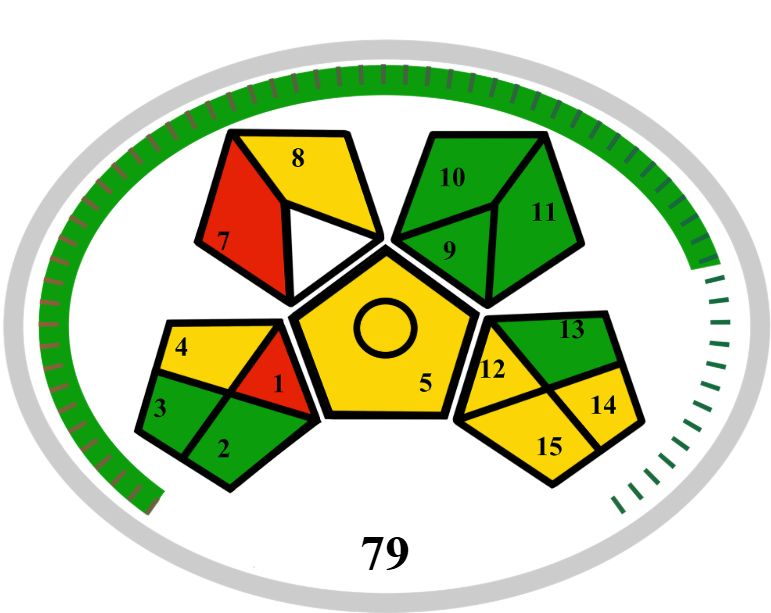 | 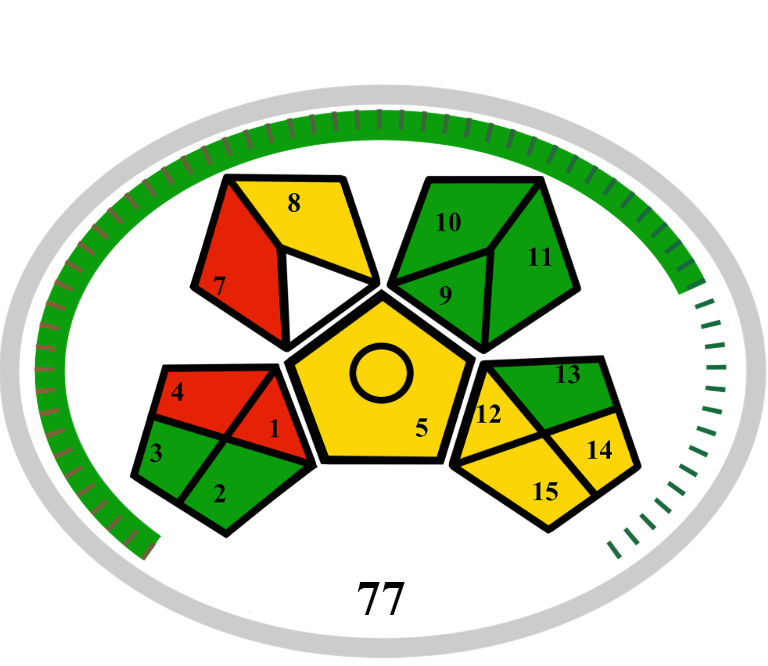 | 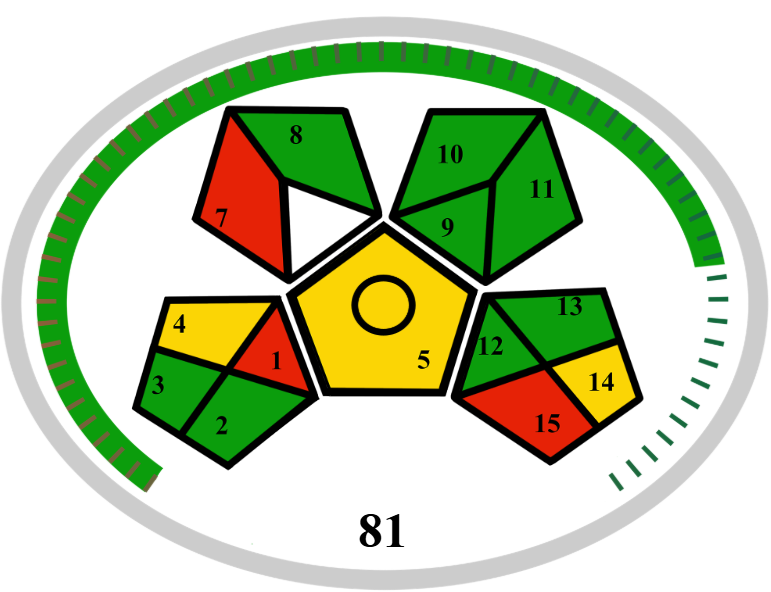 |

# Table S2. Transformation of Raw Data to Coded Levels.

| Factor | Low Level (-1) | Center (0) | High Level (+1) | ΔX (Step Change) | Coding Formula |
| --- | --- | --- | --- | --- | --- |
| A: Ethanol Ratio (%) | 60 | 65 | 70 | 5 | x = (X - 65) / 5 |
| B: pH | 4.0 | 4.25 | 4.5 | 0.25 | x = (X - 4.25) / 0.25 |
| C: Column Temperature (°C) | 20 | 25 | 30 | 5 | x = (X - 25) / 5 |
| D: Flow Rate (mL/min) | 0.25 | 0.30 | 0.35 | 0.05 | x = (X - 0.30) / 0.05 |

**Table S3.** Comparative Overview of Sustainability Assessment Tools.

| **Tool** | **Purpose** | **Scoring System** | **Thresholds** | **Reference** |
| --- | --- | --- | --- | --- |
| MoGAPI (Modified Green Analytical Procedure Index) | Evaluates overall greenness of analytical methods by integrating GAPI and Eco-Scale principles. | 0–100 scale based on penalty points and visual criteria. | ≥75: Excellent 50–74: Acceptable <50: Inadequate | **[**21**]** |
| BAGI (Blue Applicability Grade Index) | Measures method practicality across 10 weighted attributes (e.g., automation, throughput, reagent use). | 0–100 scale based on attribute scoring. | ≥75: High applicability 50–74: Moderate <50: Limited | **[**22**]** |
| MoGSA (Modified Green Star Area) | Assesses compliance with selected green chemistry principles in a flexible, context-sensitive manner. | Percentage score based on fulfilled principles. | >85: High greenness 60–85: Moderate <60: Low | **[**23**]** |

**Table S4.** Results of Intra-day and inter-day precision for CLO in dosage form.

| Test | **CLO** | |
| --- | --- | --- |
|  | **1^st^ analyst (within a day)** | **2^nd^ analyst (Between days)** |
| Test 1 | 100.2 | 99.2 |
| Test 2 | 100.4 | 99.5 |
| Test 3 | 100.1 | 99.7 |
| Test 4 | 100.8 | 100.5 |
| Test 5 | 99.3 | 100.6 |
| Test 6 | 99.8 | 99.7 |
| Average | 100.2 | 99.9 |
| RSD | 0.55 | 0.62 |
| Pooled RSD (12 samples) | 0.59 | |

**Table S5.** Robustness evaluation of the developed RP-UPLC method for CLO under small deliberate variations in analytical parameters .

| Parameter | **UPLC** | **Limit %** |
| --- | --- | --- |
|  | **CLO** | **RSD ≤ 2.0%** |
| Day to Day | 0.9 |  |
| Analyst to Analyst | 1.3 |  |
| Column to Column | 0.8 |  |
| Flow rate change (0.3 ±0.1 mL/min) | 0.6 |  |
| pH changes of mobile phase (4.25 ±0.1) | 0.7 |  |
| Fresh Sample | 0.2 |  |
| Stored Sample in fridge  Stored Sample in autosampler | 0.4  0.7 |  |
| Stored Sample at room temperature | 0.8 |  |

**Table S6.** Statistical analysis of the suggested UPLC and the reported technique for CLO quantification in its dosage form.

| **Reported Method** | **Proposed Method** | **UPLC** |
| --- | --- | --- |
| 100.60 | 101.07 | Mean |
| 0.90 | 1.1 | SD |
| - | 0.55 | *t*-Test (2.23) *^a^* |
| - | 1.49 | *F-Value* (5.05) *^a^* |

*^a^* the values between parentheses correspond to the theoretical values of *t* and *F* (*α* = 0.05)
